# Ligand-based design and synthesis of *N'*-Benzylidene-3,4-dimethoxybenzohydrazide derivatives as potential antimicrobial agents; evaluation by *in vitro*, *in vivo, and in silico* approaches with SAR studies

**DOI:** 10.1080/14756366.2022.2063282

**Published:** 2022-04-18

**Authors:** Rogy R. Ezz Eldin, Marwa A. Saleh, Mohammad Hayal Alotaibi, Reem K. Alsuair, Yahya A. Alzahrani, Feras A. Alshehri, Amany F. Mohamed, Shaimaa M. Hafez, Azza Ali Althoqapy, Seham K. Khirala, Mona M. Amin, Yousuf A. F, Azza H. AbdElwahab, Mohamed S. Alesawy, Ayman Abo Elmaaty, Ahmed A. Al-Karmalawy

**Affiliations:** a Pharmaceutical Organic Chemistry Department, Faculty of Pharmacy, Port Said University, Port Said, Egypt; b Pharmaceutical Organic Chemistry Department, Faculty of Pharmacy (Girls), Al-Azhar University, Cairo, Egypt; c National Center for Chemical Technologies, King Abdulaziz City for Science and Technology, Riyadh, Saudi Arabia; d Department of Anatomy and Embryology, Faculty of Medicine for Girls, Al-Azhar University, Cairo, Egypt; e Department of Microbiology and Immunology, Faculty of Medicine for Girls, Al-Azhar University, Cairo, Egypt; f Department of Pharmacology, Faculty of Medicine for Girls, Al-Azhar University, Cairo, Egypt; g Department of Physiology, Faculty of Medicine for Girls, Al-Azhar University, Cairo, Egypt; h Pharmaceutical Medicinal Chemistry and Drug Design Department, Faculty of Pharmacy (Boys), Al‐Azhar University, Cairo, Egypt; i Department of Medicinal Chemistry, Faculty of Pharmacy, Port Said University, Port Said, Egypt; j Department of Pharmaceutical Medicinal Chemistry, Faculty of Pharmacy, Horus University-Egypt, New Damietta, Egypt

**Keywords:** *N'*-benzylidene-3,4-dimethoxybenzohydrazide, antibacterial, antifungal, *in vitro*, *in vivo*, SAR

## Abstract

Herein, a series of *N'*-benzylidene-3,4-dimethoxybenzohydrazide derivatives were designed and synthesised to target the multidrug efflux pump (MATE). The antibacterial activities were screened against *S. aureus*, *Acinetobacter*, *S. typhi*, *E. coli*, and *P. aeruginosa*, whereas their antifungal activities were screened against *C. albicans*. Compounds **4a**, **4h**, and **4i** showed the most promising antibacterial and antifungal activities. Moreover, compounds **4h** and **4i** being the broader and superior members regarding their antimicrobial effects were selected to be further evaluated via *in vivo* testing using biochemical analysis and liver/kidney histological examination. Additionally, molecular docking was carried out to attain further deep insights into the synthesised compounds' binding modes. Also, ADMET studies were performed to investigate the physicochemical/pharmacokinetics features and toxicity parameters of the synthesised derivatives. Finally, a structure-antimicrobial activity relationship study was established to facilitate further structural modifications in the future.

**Highlights**
- A series of new *N'*-benzylidene-3,4-dimethoxybenzohydrazide derivatives were designed and synthesised targeting the multidrug efflux pump (MATE) guided by the pharmacophoric features of the co-crystallized native inhibitor of the target protein.
- The newly synthesised compounds were assessed through *in vitro*, *in vivo*, and *in silico* approaches.
- Using the agar well diffusion assay, the antibacterial activities of the synthesised compounds were screened against *S. aureus*, *Acinetobacter*, *S. typhi*, *E. coli*, and *P. aeruginosa*, whereas, their antifungal activities were screened against *C. albicans*.
- The minimal inhibitory concentration (MIC) and the minimal bactericidal concentration (MBC) of the synthesised compounds were investigated on variable microbial species.
- Compounds (**4h** and **4i**) -as the broader and superior members regarding their antimicrobial effects- were further evaluated via *in vivo* testing using bio-chemical analysis and liver/kidney histological examination.
- A molecular docking study and ADMET *in silico* studies were performed.
- A structure-antimicrobial activity relationship study was established to facilitate further structural modifications in the future.

## Graphical Abstract

**Figure:**
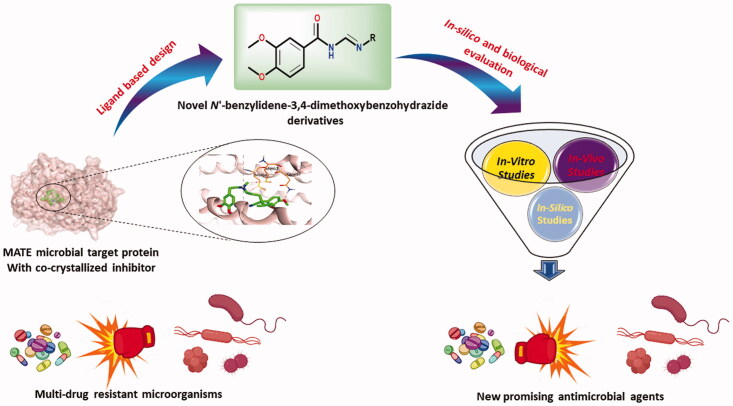

## 1. Introduction

Mostly, humans coexist peacefully with the microorganisms that surround them. But, when the immune system is compromised or pathogen concentrations reach a critically high density, an infection may occur^1^. The considerable efforts devoted to microbial diseases diagnosis and treatment during the past 50 years have driven spectacular gains. Hence, a range of therapeutic intervention strategies was introduced for clinical practice^2^. Besides, many studies have looked over likely correlations between gut microbiota and intestinal diseases such as inflammatory bowel diseases, and Crohn’s disease^3^. Moreover, bacterial pneumonia was possibly the main cause of death among the elderly, until the mid-twenty century. However, mortality rates from bacterial infections have been lowered by ameliorated sanitation, vaccines, and antibiotics^4^. As strategies to curb bacterial infections in humans progressed, fungi became one of the most hazardous pathogens. Yeasts and moulds are now among the top ten pathogens frequently isolated from patients in intensive care units^1^^,^^5^.

**Figure 1.**
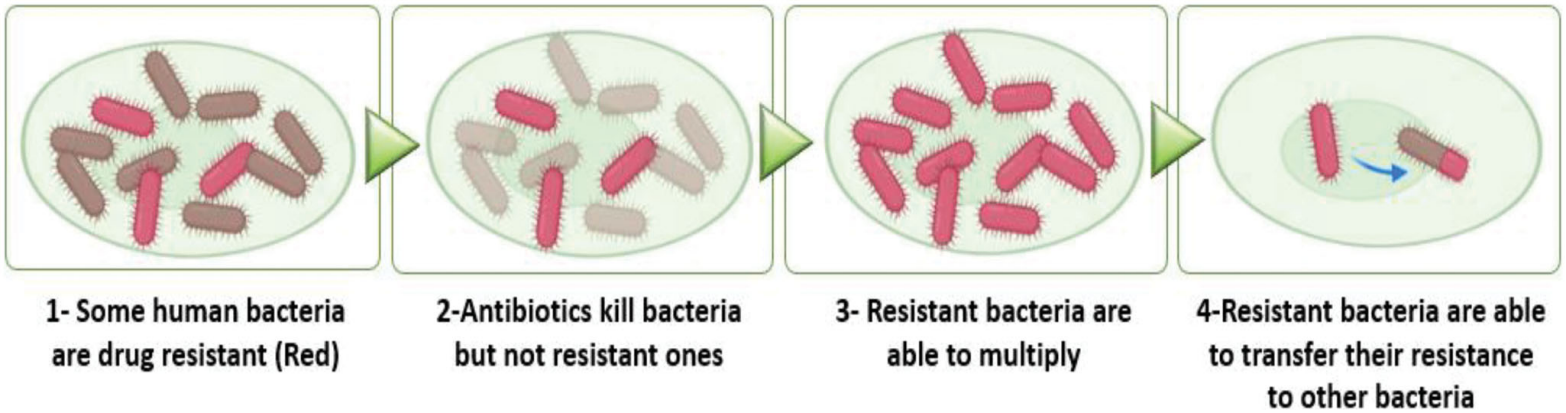
A diagrammatic representation revealing the steps of antibiotic resistance.

On the other hand, the multidrug resistance (MDR) within bacteria and fungi is an alarming microbial threat and induces a major global public health concern^6^. A wide variety of mechanisms may lead to the emergence of bacterial resistance, including resistance genes acquisition via mutations and horizontal gene transfer^7^. Besides, antibiotics overuse and misuse without suitable medical guidance speed up the expansion and emergence of multidrug-resistant bacteria^8–10^. By 2050, it is estimated that the death toll nears 10 million per year by drug-resistant infections if there are no new steps to be taken^11^. Many bacterial strains have developed advanced mechanisms, which permit them to survive and eliminate antibiotic effects. As a result, some isolated strains of *Staphylococcus aureus* (*S. aureus*) have evolved intrinsic resistance to many antibiotics, such as β-lactams, aminoglycosides, glycopeptides, and fluoroquinolones^7^. Resistance revealed by some *Acinetobacter baumannii* species to some currently available antibiotics is may be attributed to their capability to adjust and promote multiple resistance strategies, such as antibiotics β-lactam rings hydrolysis, antibiotics entry reduction into bacteria target sites, and bacterial targets change by mutations^12^^,^^13^. Besides, *Pseudomonas aeruginosa* (*P. aeruginosa*) may show high resistance to some antibiotics through its ability to stay in aggregates forming biofilms^14^. Steps for antibiotic resistance are depicted in Figure 1.

Although bacterial antibiotic resistance is a well-known public health concern, fungal resistance revealed by fungal infections is arising issue as well^11^. Hence, the development of some new molecules with enhanced bioactivity has become an urgent requirement^6^^,^^15^. Thereby, molecular hybridisation has come out as one of the best strategies to synthesise new efficient lead compounds with new action modes improving their biological activity^16^. Molecular hybridisation relies on gathering two or more investigated bioactive pharmacophores into a single hybrid molecule with enhanced efficacy and affinity compared to the parent compounds. So, different targets may be affected by a single molecule^6^^,^^17–19^. Additionally, to overcome microbial resistance, existing drugs modification appears to be one of the preferred approaches so far. Thus, most compounds under clinical investigation are refined versions of already approved drugs^20^^,^^21^.

Moreover, to maintain cellular homeostasis, the exogenous toxic compounds extrusion is primary for all life kinds. This extrusion process is fulfilled via transporters (efflux pumps) that can export xenobiotics^22^. Based on amino acid composition similarity and energy source, six families of these multidrug transporters have been unveiled^22^^,^^23^. Antibiotics and other xenobiotics can be extruded from the cytoplasm or surrounding membranes of cells to the external environment via efflux pumps, which are proteinaceous transporters. These efflux pumps are revealed in all microorganisms, including gram-positive and gram-negative bacteria^24^. Recently, efflux pumps have attained considerable attention and appeared as a pivotal resistance determinant owing to their prevalent distribution among different bacterial species, which is an alarming threat to antibiotics therapy^23^^,^^24^. Out of these transporter families, the multidrug and toxic compound extrusion (MATE) family was identified as one of the important factors responsible for bacterial resistance^22–25^. MATE was recognised at first as the main cause of *Vibrio parahaemolyticus* multidrug resistance. Besides, MATE is reportedly in charge of *S. aureus* multidrug resistance, which is the main cause of hospital infections. Thereby, MATE has acquired significant attention^22^.

**Scheme 1.**
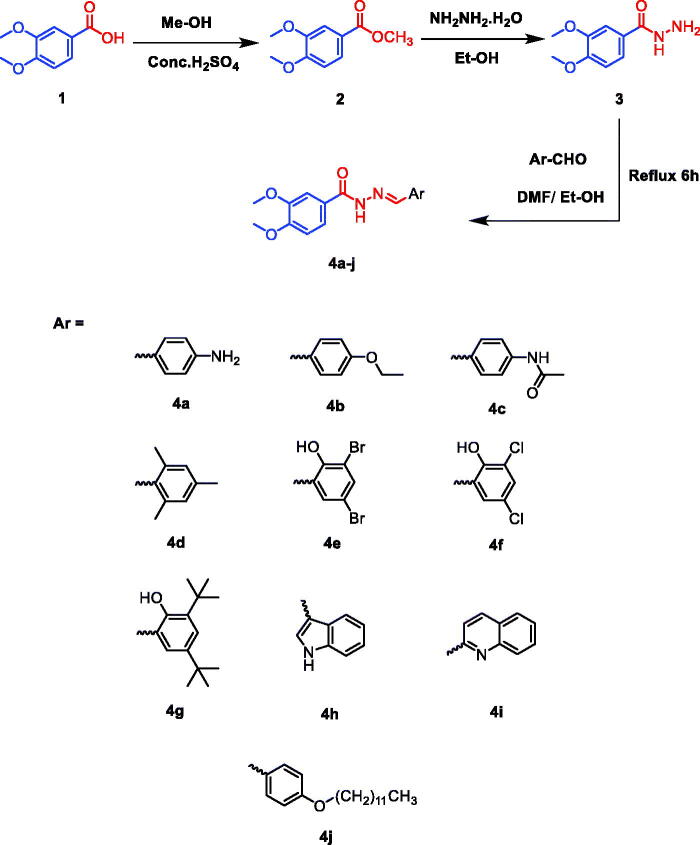
Synthesis of the target compounds (**4a–4j**).

Furthermore, the literature review looking over antimicrobial agents revealed that benzohydrazide derivatives were recently designed and synthesised with promising antibacterial and antifungal activities^26–30^. So, in this regard, we synthesised a series of novel 3,4-dimethoxybenzo- hydrazide derivatives having the same pharmacophoric features of MATE inhibitors as depicted in Scheme 1 revealing their potential as antimicrobial agents with considerable inhibitory effects overcoming possible bacterial resistance. The newly synthesised compounds were looked deeply into their efficacy using *in silico*, *in vitro,* and *in vivo* approaches. On the other hand, the cytotoxicity assay was performed for the tested compounds using a normal mammalian Vero cell line by the MTT assay^31^^,^^32^.

### 1.1. The rationale work design

**Figure 2.**
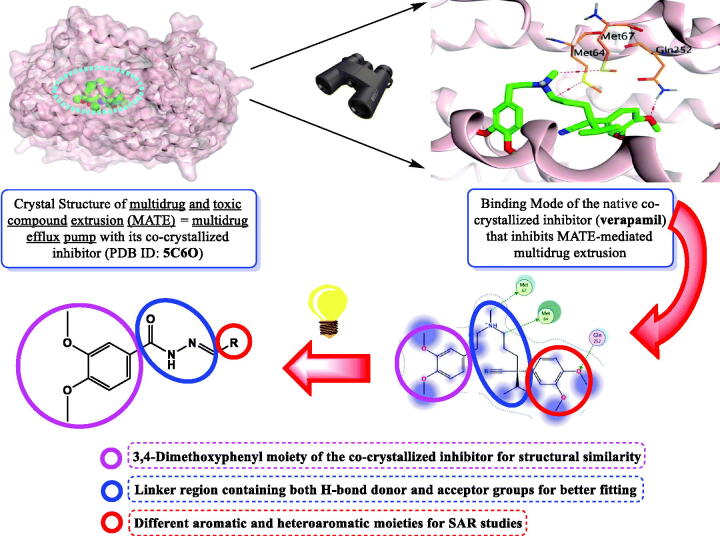
Schematic representation describing the design of *N'*-benzylidene-3,4-dimethoxybenzohydrazide derivatives as promising antimicrobial candidates targeting the multidrug efflux pump (MATE).

Supported by the previously mentioned facts, we designed and synthesised a novel series of *N'*-benzylidene-3,4-dimethoxybenzohydrazide derivatives to act as promising antimicrobial candidates targeting the multidrug efflux pump (MATE) based on the structural similarity between the designed compounds and the co-crystallized native inhibitor (verapamil) of the target protein (Figure 2).

Notably, the co-crystallized inhibitor of the MATE target protein was found to get stabilised inside its binding pocket through three crucial pharmacophoric features;

- A 3,4-dimethoxyphenyl moiety is to be inserted inside the largest hydrophobic region of the receptor pocket.
- A linker region containing both an H-bond donor and acceptor to form H-bonds with Met64 and Met67 amino acids for better fitting.
- A second 3,4-dimethoxyphenyl moiety is to be inserted inside the second hydrophobic region of the receptor.

Herein, the structural modifications based on the above discussed pharmacophoric features depended on the presence of a 3,4-dimethoxyphenyl moiety to be inserted similarly inside the largest hydrophobic pocket of the receptor as previously described. In addition, a linker region was designed as a keto hydrazide moiety to act as an H-bond donor and/or acceptor in the region of the receptor pocket similar to that of the co-crystallized inhibitor. Besides, the second 3,4-dimethoxyphenyl moiety was replaced by different aromatic or heteroaromatic moieties (benzene, indole, or quinoline) substituted with different electron-donating and/or withdrawing groups hoping to get new compounds with better fitting inside the aforementioned second hydrophobic region of the receptor pocket (Figure 2).

Notably, the previously discussed structural modifications were appointed for adequate structure-antimicrobial activity relationship (SAR) studying the effect of such variations on both the spectrum and the degree of antimicrobial activities.

## 2. Results and discussion

### 2.1. Chemistry

Targeted *N*'-benzylidene-3,4-dimethoxybenzohydrazide derivatives were synthesised via a three-step reaction outlined in Scheme 1. The reaction series first involved the esterification of 3,4-dimethoxybenzoic acid **1** with sulphuric acid and methanol, which affords the corresponding methyl ester derivative **2**. Hydrazinolysis of the formed ester **2** with hydrazine hydrate in ethanol afforded the hydrazide derivative **3**. Condensation of hydrazide derivative **3** with different aromatic aldehydes furnished the corresponding hydrazone derivatives (**4a–j**) with a good yield and high purity. This method of preparation was in accordance with similar previously discussed methods^32–35^. The synthesised products were characterised using IR, ^1^H NMR, ^13 ^C NMR, MS, and Elemental analyses. All the spectral data were in accordance with the assumed structures as depicted in the supplementary information (**SI1**). Variation was introduced in the molecules at the hydrazide by varying the aromatic aldehydes used to form the benzylidene moiety. Ten different aldehydes were used in the synthetic methodology.

First regarding (*E*)-*N'*-(4-aminobenzylidene)-3,4-dimethoxy- benzohydrazide (**4a**) the yield of the product is good (83%), after purification by recrystallization. Its IR spectrum displayed a characteristic band for amino groups at 3370 cm^−1^ and amidic carbonyl group at 1660 cm^−1^. Its ^1^H NMR spectrum explained the presence of two methoxy groups at *δ* 3.37, 3.45 ppm integrating for 6 protons. The protons of the amino group were observed at *δ* 5.45 ppm which was exchanged by D_2_O. Additionally, the ^1^H NMR spectrum of **4c** showed one singlet signal at 2.02 ppm due to CH_3_ protons, whereas the singlet signal for the proton of CH = N at *δ* 8.56 ppm. Moreover, the ^13 ^C NMR spectrum of (*E*)-*N'*-(2,4,6-trimethylbenzylidene)-3,4-dimethoxybenzohydrazide (**4d**) showed the presence of 18 different carbons. The signal for one carbonyl at *δ* 162.75 ppm, signals for methoxy carbons at *δ* 56.12, 56.22 ppm, and signals for methyl carbons at *δ* 29.80, 31.73 ppm in addition to aromatic carbons. The mass spectrum of **4f** exhibited a molecular ion peak at m/z = 369 (M^+.^, 18.32%), 371 (M ^+ 2^, 6.13%), and 373 (M ^+ 4^, 18.33%) corresponding to the molecular formula C_16_H_14_Cl_2_N_2_O_4_. Almost similar patterns were noticed in the ^1^H NMR and ^13 ^C NMR spectra of all the newly synthesised compounds of this series.

### 2.2. Biological evaluation


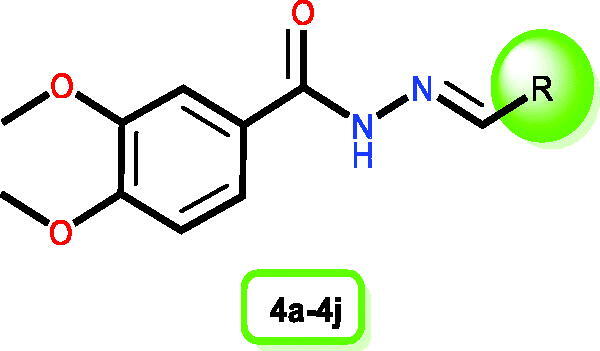


#### 2.2.1. *In vitro* antibacterial and antifungal activity testing

##### 2.2.1.1. Agar well diffusion assay

The agar well diffusion assay was first done to evaluate the antimicrobial activities of the different target compounds (**4a–j**) against gram-positive bacteria, gram-negative bacteria, and fungi (Table 1A). The zones of inhibition were measured in cm and the obtained results indicated that the synthesised compounds exhibited both antibacterial and antifungal activities against the tested microorganisms. First, the antibacterial activities were tested against *S. aureus* ATCC 6538, *Acientobacter* (*A. baumanii*) ATCC 19606, *S. typhi*, *E. coli*, and *P. aeruginosa* as examples of the most common gram-positive and gram-negative bacteria, in comparison to vancomycin and ceftriaxone as two reference standards. In addition, the antifungal activities were screened against *C. albicans*, in comparison to amphotericin as a reference standard. Interestingly, compound **4h** with an indolyl side chain showed the best and broadest inhibitory activities against both the tested bacteria and fungi as well. It achieved the best activities, especially against *S. aureus*, *S. typhi*, and *P. aeruginosa*. On the other hand, compound **4i** with a quinolinyl side-chain achieved the most inhibitory activity against *A. baumanii* exceeding that of ceftriaxone itself with superior inhibitory activity against *E. coli* as well. Moreover, compound **4a** with *p*-amino phenyl side chain exhibited the best antifungal activity against *C. albicans* (Table 1A). The antibacterial activities of some tested compounds (**4a**, **4b**, **4h**, and **4e**) against gram-positive and gram-negative bacteria by agar well diffusion assay are represented in the supplementary data **(**Figure SI1).

**Table 1.** Agar well diffusion assay, MIC, and MBC for the target compounds (**4a–4j**) compared to vancomycin, ceftriaxone, and amphotericin B as reference standards (**1A**, **1B**, and **1C**, respectively).

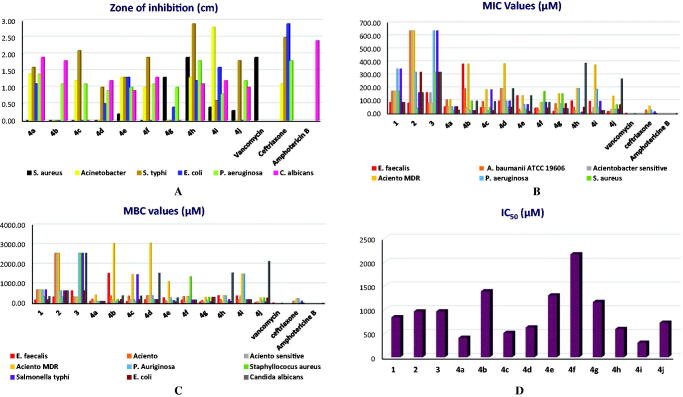

Besides, IC_50_ values of the target compounds (**4a–4j**), **1D**.

##### 2.2.1.2. Minimal inhibitory concentration (MIC)

The minimal inhibitory concentration (MIC) of each tested compound was evaluated to identify the minimum concentration required to inhibit *E. faecalis*, *A. baumanii* ATCC 19606, *Acientobacter* sensitive, *Aciento* MDR, *P. aeruginosa*, *S. aureus*, *S. typhi*, *E. coli*, and *C. albicans* (Table 1B). It revealed that the investigated compounds displayed diverse activities against the different microbial species. Notably, compound **4a** showed better MIC values (26.11 µM) towards *S. aureus* and *C. albicans*. However, compound **4b** attained better MIC values (23.28 µM) against *P. auriginosa*, *S. typhi*, and *E. coli*. Moreover, compound **4c** displayed the best MIC value (22.89 µM) against *P. auriginosa*. Furthermore, compound **4d** revealed better MIC values (47.83 µM) for *S. aureus* and *E. coli*. It is worth mentioning that compounds **4e** and **4f** disclosed promising MIC values (17.13 and 21.22 µM, respectively) against *Acientobacter sensitive* and so close to the MIC value of the control ceftriaxone (14.08 µM). Notably, compound **4g** revealed the best MIC value (18.95 µM) against *E. faecalis*. Interestingly, compound **4h** showed a promising MIC value (12.07 µM) against *S. typhi* and was better than the MIC value of ceftriaxone (14.08 µM). Besides, compound **4h** revealed an outstanding MIC value (5.88 µM) against *S. aureus* and so close to the MIC value of ceftriaxone (3.52 µM). Compound **4i** showed better MIC values against *A. baumanii* ATCC 19606, *E. coli*, and *C. albicans* at concentrations 11.64, 23.30, and 23.30 µM, respectively. Finally, compound **4j** disclosed a better MIC value (16.68 µM) against *E. faecalis* and *A. baumanii* ATCC 19606.

##### 2.2.1.3. Minimal bactericidal concentration (MBC)

After determining the MIC for the target compounds, the minimal bactericidal concentration (MBC) for each compound that is required to kill each one of the above-mentioned microorganisms was assessed. The obtained MBC values for the tested compounds revealed the following interesting results (Table 1C):

Compound **4a** showed the best MBC values (104.60 µM) against *E. faecalis*, *Aciento sensitive*, *P. Auriginosa*, *S. aureus*, *S. typhi*, *E. coli*, and *C. albicans*. Interestingly, compound **4a** disclosed better MBC values against *Aciento sensitive* and *S. typhi* than ceftriaxone. Moreover, compound **4b** revealed the best MBC value (95.39 µM) for *P. Auriginosa* and *S. typhi* better than MBC values of ceftriaxone (225.4 µM). In addition, compound **4c** showed a better MBC value (91.75 µM) against *E. faecalis* and *S. aureus*, whereas, compound **4d** disclosed a better MBC value (191.60 µM) against *E. faecalis*, *S. aureus*, *S. typhi*, and *E. coli*. It is worth mentioning that compound **4e** attained a better MBC value (68.65 µM) towards *Aciento sensitive* and *E. coli*. Interestingly, compound **4e** revealed a better MBC value against *E. coli* than ceftriaxone (112.70 µM). Additionally, compound **4f** displayed the best MBC value (85.05 µM) against *Aciento sensitive* and better than ceftriaxone (112.70 µM), whereas, compound **4g** showed a better MBC value (75.93 µM) against *E. faecalis*, *Aciento sensitive*, and *S. typhi*. Moreover, compound **4g** attained better MBC values against *Aciento sensitive* and *S. typhi* than ceftriaxone. Besides, compound **4h** disclosed a better MBC value (96.87 µM) against *Aciento sensitive*, *S. aureus*, and *E. coli*. Also, compound **4h** attained better MBC values against *Aciento sensitive* than ceftriaxone (112.70 µM). In addition, compound **4i** displayed better MBC values (186.50 µM) against *A. baumanii*, *S. aureus*, *S. typhi*, *E. coli*, and *C. albicans*. Finally, compound **4j** showed the best MBC value (33.31 µM) against *E. faecalis*. It is worth also mentioning that compound **4j** attained a better MBC value (66.84 µM) against *A. baumanii*, *Aciento sensitive*, and *S. typhi* than ceftriaxone (112.70 µM).

##### 2.2.1.4. Determination of sample cytotoxicity on cells using MTT assay

Moreover, the cytotoxicity values of the target compounds were determined to calculate their corresponding IC_50_ values through the MTT assay. The IC_50_ value indicates the compound’s concentration that is required to inhibit 50% of the target cells. It was observed that both compounds **4a** and **4i** showed the lowest IC_50_ values (406.8 and 304.7 µM, respectively). However, compound **4f** exhibited the highest IC_50_ value (2164.4 µM) as depicted in Table 1D.

#### 2.2.2. *In vivo* testing

##### 2.2.2.1. Biochemical analysis

As shown in Table 2A, rats infected with *S. aureus* had severe deterioration in renal and liver functions (significant elevation of urea, creatinine, ALT, and AST) as well as marked oxidative stress (significant disturbance in GSH and MDA) and inflammatory marker changes (significant elevation of TNF-α and depression of IL-10). Fortunately, rats treated with **4h** compound showed significant improvement in all the measured parameters. However, neither the first dose nor the second dose could return any of the parameters to normal.

**Table 2.** **(A)** Effect of compound **4h** on the improvement of both renal and liver parameters in *S. aureus*-infected rats and **(B)** Effect of compounds **4h** and **4i** on the improvement of both renal and liver parameters in *S. typhi* infected rats (*p* values ˂ 0.05).

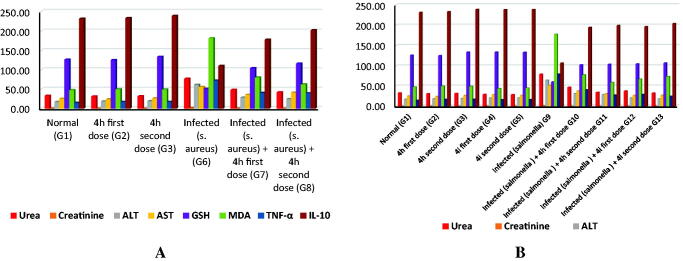

Moreover, as indicated in Table 2B, it was revealed that rats infected with *S. typhi* exhibited a manifested reduction in renal and liver functions (significant elevation of urea, creatinine, ALT, and AST) beside marked oxidative stress (significant decrease in GSH and elevation of MDA) as well as changes in the inflammatory markers (significant elevation of TNF-α and depression of IL-10). However, the first dose of the **4h** drug succeeded to make a significant improvement in all measured parameters, but none of them could be returned to normal. Obviously, rats treated with the second dose of **4h** or **4i** compounds (first and second doses) showed marvellous amelioration in all parameters up to returning of urea, creatinine, AST, and TNF-α to normal levels (if treated with **4h** second dose), normalisation of urea, ALT, and AST (if treated with the **4i** first dose) as well as returning of urea, creatinine, ALT, and AST (if treated with the **4i** second dose) to normal levels.

This study is a trial to confirm the safety and clinical efficacy of *N'*-benzylidene-3,4-dimethoxybenzohydrazide derivatives in a rat model of infection. The current research provides novel findings and sheds light on the possible antioxidant and immunomodulatory effects of the most promising two candidate compounds (**4h** or **4i**) in this experimental model of bacterial infection.

The current work revealed that the rats infected with *S. aureus* or *S. typhi* exhibited a significant increase in lipid peroxidation radicle byproduct (i.e. evaluated by serum malondialdehyde) versus a marked decrement in “master antioxidant”, serum GSH. In addition, altered host immunity was reported by a marked increment in the pro-inflammatory cytokines (TNF-α) versus a significant decrease in IL-10 (i.e. anti-inflammatory cytokine). In addition, there was significant organ deterioration of both liver function (i.e. elevated serum ALT and AST) and kidney function (i.e. increased serum levels of urea and creatinine), which might be because of free-radical-mediated membrane damage. In addition, the chemical analysis of the current study demonstrates that **4i** and the second dose of **4h** (as combination therapy) are more effective in treating *S. typhi* infection, while both doses of **4h** compound produce smaller improvement in *S. aureus*-infected rats.

Interestingly, in comparison to positive control infected groups, we observed that treatment of infected rats with *N'*-benzylidene-3,4-dimethoxybenzohydrazide derivatives (**4h** or **4i**) restored the redox balance, as indicated by a decreased serum level of MDA versus an increase in antioxidant serum GSH. In addition to the immunomodulatory effect of treatment was proved by the reduction of TNF-α versus an increase in serum IL-10. Moreover, restoration of organs (i.e. liver and kidney) function was noticed by a decrease in the level of liver enzymes (ALT and AST) and serum urea and creatinine were also decreased. This could be attributed to the restoration of oxidant/antioxidant balance, which might be because of its free-radical-scavenging ability. These results were consistent with the previous studies by Wang et al.^36^.

The ameliorative impact of the *N'*-benzylidene-3,4-dimethoxybenzohydrazide derivatives (**4h** or **4i**) against the abovementioned pathogenesis of infection could be attributed to the ability of such derivatives to counteract oxidative stress and boost immunity, and therefore a safeguard against organ misfunction can be attained.

##### 2.2.2.2. Histological examination of the liver and kidney

###### 2.2.2.2.1. General histological examination

**Figure 3.**
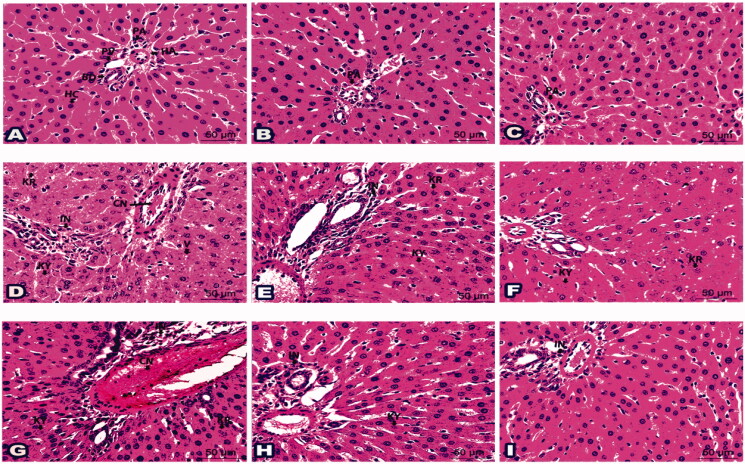
Photomicrographs of liver sections represented the impact of ‘**4h**’ drug in all inspected groups: (A) Histologic liver section from negative G1 displayed normal portal area (PA); portal vein (PV), hepatic artery (HA), and bile duct (BD) plus hepatic cords (HC). Liver sections from G2 (B) and G3 (C) exposed typical hepatic structures like G1. (D) Histological liver section from *S. aureus* infected G6 suffered hepatocytes with karyorrhexis (KR), karyolysis (KY), cytoplasmic vacuolation (V), aggregated inflammatory cells (IN), and congested blood vessels (CN). (E) Sections from G7 established partial parts with karyorrhexis (KR), karyolysis (KY) in addition some inflammatory cells (IN). (F) Sections from G8 were noticed with better tissue and a rare nucleus with karyorrhexis (KR) and karyolysis (KY). (G) Histological liver section from *S. typhi* infected G9 revealed karyorrhexis (KR) and karyolysis (KY) of hepatic cells besides inflammatory cells (IN) and congested blood vessels (CN). (H) Sections from G10 specified some karyolitic cells (KY) along with a few inflammatory cells (IN). (I) Sections from G11 highlighted with few inflammatory cells (IN). (H&E staining, 400x Magnification, Scale bar = 50 μm).

**Figure 4.**
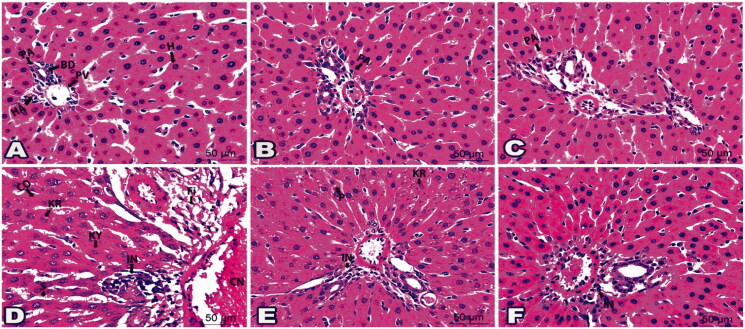
Photomicrographs of histological liver sections showed the validity of drug ‘**4i**’ in all tested groups: (A) Liver section from G1 demonstrated typical portal area (PA) enclosing portal vein (PV), hepatic artery (HA), and bile duct (BD). Notice hepatocyte (H) organised in cords. Histological liver sections from G4 (B) and G5 (C) displayed a picture looking like G1. (D) Liver section from G9 marked disarranged hepatic cords (CO), nuclear changes; karyorrhexis (KR) and karyolysis (KY), dilated sinusoids (S), aggregated inflammatory cells (IN) and fibres (Fi), in addition, congested blood vessel (CN). (E) Sections from liver-treated G12 restored better architecture except few hepatocytes seemed with karyorrhexis (KR) or pyknotic (P) nucleus. Notice inflammatory cells (IN). (F) Liver sections from G13 pointed only to mild inflammatory cells (IN). (H&E staining, 400x Magnification, Scale bar = 50 μm).

Examination of H & E liver sections from control groups (Gs 1, 2, and 3) demonstrated a normal histological structure of the portal area enclosing the portal vein, the hepatic artery, and the bile duct. The hepatocytes were organised in regular cords separated by the hepatic sinusoids, Figures 3 and 4(A).

Sections from **4h** treated groups (Gs 4 and 5) and **4i** treated groups (Gs 6 and 7), Figures 3 and 4(B and C) showed a normal histological structure similar to those of the control groups, Figures 3 and 4(A). However, liver sections from *S. aureus* and *S. typhi* infected groups (Gs 8 and 11, respectively) showed marked pathological changes as cytoplasmic vacuolation and nuclear changes in the form of karyorrhexis and karyolysis of the hepatocytes. Distorted hepatic cords, dilated hepatic sinusoids, marked inflammatory cells aggregation around portal areas, and congested portal vein was also noticed, Figures 3(D and G) and 4D.

Notably, the liver of *S. aureus* and *S. typhi* infected groups treated with **4h** low dose (Gs 9 and 12, respectively), Figure 3(E and H) and *S. typhi* infected group treated with **4i** low dose (G14), Figure 4E showed improved pathological changes. However, some inflammatory cells around the portal areas were still present with nuclear changes in some hepatocytes. The *S. aureus* and *S. typhi* infected groups treated with **4h** high dose (Gs 10 and 13), and *S. typhi* infected group treated with **4i** high dose (G15) showed marked improvement in the pathological changes with few inflammatory cells, Figures 3(F and I) and 4F.

**Figure 5.**
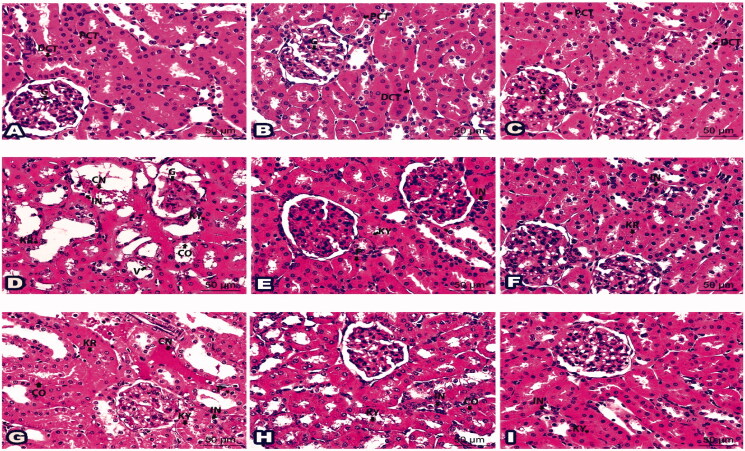
Photomicrographs of kidney sections demonstrated the outcome of ‘**4h**’ drug in all examined groups: (A) Histologic kidney section G1 showed normal glomerulus (G), proximal convoluted tubules (PCT), and distal convoluted tubules (DCT). Kidney sections from G2 (B) and G3 (C) revealed photographs similar to G1. (D) Histological kidney section from infected group G6 displayed cytoplasmic and nuclear changes in the glomerulus (G) and renal tubules constituents (CO); karyorrhexis (KR), karyolysis (KY), cytoplasmic vacuolation (V), aggregated inflammatory cells (IN), and congested blood vessels (CN). (E) Sections from G7 posed limited cells with pyknosis (P) and karyolysis (KY), besides some inflammatory cells (IN). (F) Sections from G8 were marked with few inflammatory cells (IN) and renal tubule cells with karyorrhexis (KR). (G) Histological kidney section from salmonella infected group (G9) exhibited disruption in renal tubule constituents (CO) with pyknosis (P), karyorrhexis (KR), karyolysis (KY), infiltration of inflammatory cells (IN), and congested blood vessels (CN). (H) Sections from G10 developed few disturbances in renal tubule constituents (CO); karyolysis (KY) as well a few inflammatory cells (IN). (I) Sections from G11 highlighted scarce areas of karyolysis (KY) and inflammatory cells (IN). (H&E staining, 400x Magnification, Scale bar = 50 μm).

**Figure 6.**
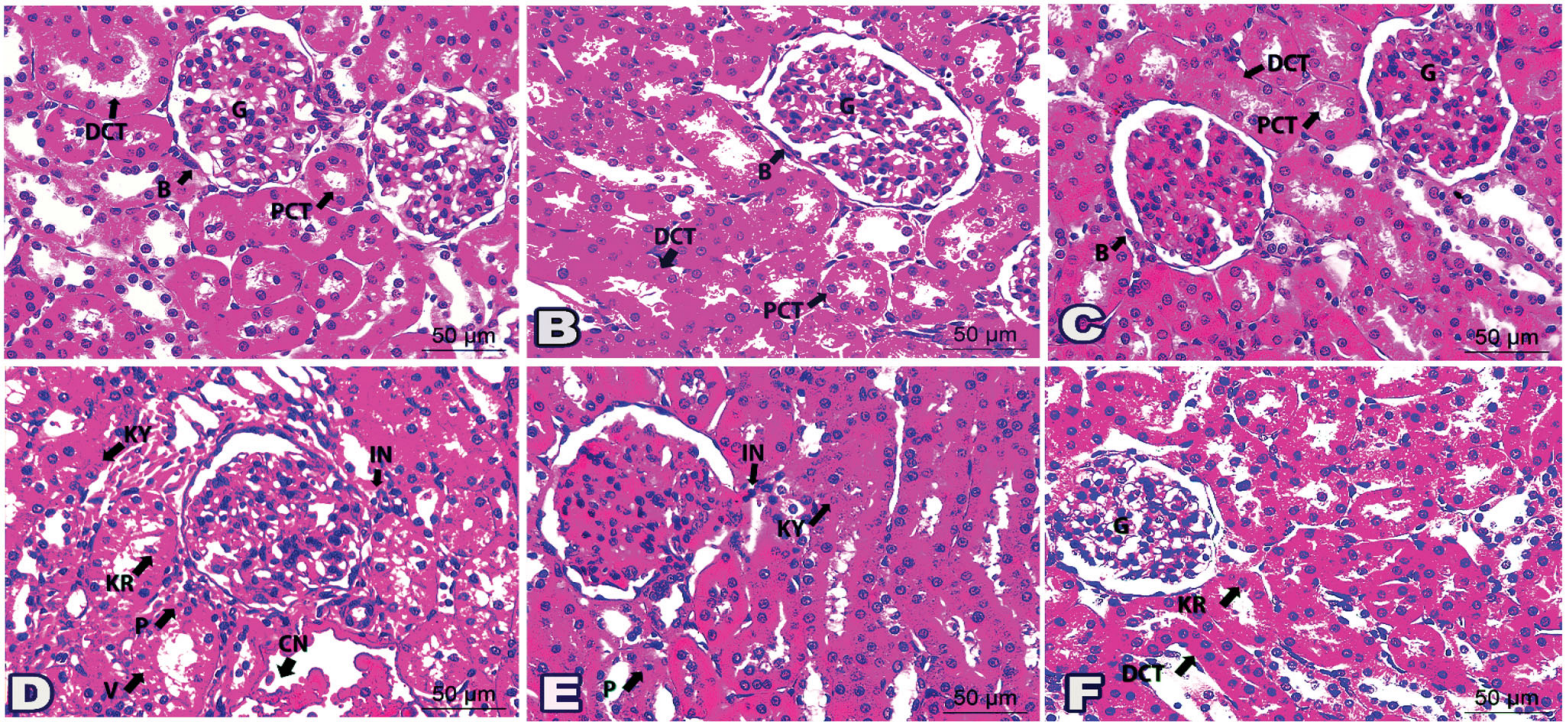
Photomicrographs of histological kidney sections presented the effect of drug ‘**4i**’ in all tested groups: (A) Kidney section from G1 showed normal glomerulus (G), bowman’s capsule (B), proximal convoluted tubules (PCT), and distal convoluted tubules (DCT). Kidney sections from G4 (B) and G5 (C) displayed a picture resembling G1. (D) Histological kidney cells from G9 exhibited a nucleus with pyknosis (P), karyorrhexis (KR), and karyolysis (KY). Notice cytoplasmic vacuolations (V), congestion (CN), and inflammatory cells infiltrations (IN). (E) Sections from G12 presented specified cells of pyknotic (P) and karyolitic (KY) nucleus besides a few inflammatory cells (IN). (F) Sections from G13 existed nearly like the normal glomerulus (G) and distal convoluted tubules (DCT). karyorrhexis (KR) was noticed lining a few cells in proximal convoluted tubules. (H&E staining, 400x Magnification, Scale bar = 50 μm).

On the other hand, the examination of H&E kidney sections of control groups (Gs 1, 2, and 3) demonstrated a normal histological structure of the renal corpuscles, proximal convoluted tubules (PCTs), and distal convoluted tubules (DCTs), Figures 5 and 6(A).

Kidney sections from **4h** low dose treated group (G4), **4h** high dose treated group (G5), **4i** low dose treated group (G6), and **4i** high dose treated group (G7), Figures 5 and 6(B and C) had a histological structure similar to those of the control groups, Figures 5 and 6(A). On the contrary, *S. aureus* and *S. typhi* infected groups (Gs 8 and 11, respectively) displayed pathological changes in the form of shrunken glomeruli with dilated Bowmans′ spaces of the renal corpuscles and thinning of the renal tubules. The epithelial cells lining the renal tubules showed vacuolation and nuclear changes in the form of pyknosis, karyorrhexis, and karyolysis. Congested blood vessels with interstitial haemorrhage and inflammatory cells infiltrations were noticed, Figures 5(D and G) and 6D.

Interestingly, the kidneys of *S. aureus* and *S. typhi* infected groups treated with **4h** low dose (Gs 9 and 12, respectively), Figures 5(E and H) and *S. typhi* infected group treated with **4i** low dose (G14), Figure 6E showed improved pathological changes. However, some renal corpuscles had dilation of the Bowman's spaces and some lining epithelial cells had vacuolated cytoplasm and pyknotic nuclei. The *S. aureus* and *S. typhi* infected groups treated with **4h** high dose (Gs 10 and 13) and *S. typhi* infected group treated with **4i** high dose (G15) showed marked improved renal tissue, Figures 5(F and I) and 6F.

###### 2.2.2.2.2. Immunohistochemical results

In this experiment, the anti-caspase 3 reaction of all groups in the liver and the kidney was represented in Figures SI2, SI3, SI4, and SI5 and Table 3. Negative reactions were detected in the control groups (Gs 1, 2, and 3), **4h** (Gs 4 and 5) and **4i** treated groups (Gs 6 and 7) as shown in Figures SI2, SI3, SI4, and SI5, (A, B, and C, respectively). The infected groups (Gs 8 and 11), Figures SI2, SI3, SI4, and SI5, (D and G) had a high positive reaction in comparison to the control groups (Gs 1, 2, and 3), Figures SI2, SI3, SI4, and SI5, (A). Besides, the infected groups injected with the 1^st^ doses of the **4h** (Gs 9 and 12) and **4i** compounds (G14), Figures SI2, SI3, SI4, and SI5, (E and H) and 2^nd^ doses (Gs 10,13, and 15), Figures SI2, SI3, SI4, and SI5, (F and I) had positive reaction less than the infected groups (Gs 8 and 11), Figures SI2, SI3, SI4, and SI5, (D and G). In addition, the groups administered the 2^nd^ doses (Gs 10, 13, and 15), Figures SI2, SI3, SI4, and SI5, (F and I) had positive reactions lower than the 1^st^ doses (Gs 9, 12, and 14), Figures SI2, SI3, SI4, and SI5, (E and H).

**Table 3.** Quantitative analysis of immune markers (Caspase 3 & NFKB2) within and between groups of tested (**4h** & **4i**) compounds to target organs (liver & kidney).

	Mean ± SE							
	Parameter							
	Caspase 3				NFKB2			
	Organ							
Groups	Liver		Kidney		Liver		Kidney	
G1	M0.6083 ± 0.19128	R0.4137 ± 0.2927	M0.3243 ± 0.10958	R0.1963 ± 0.01317	M0.246 ± 0.043097	R0.30067 ± 0.084936	M0.13467 ± 0.059038	R0.49867 ± 0.133225
G2	M0.4033 ± 0.11661	R0.656 ± 0.27084	M0.4367 ± 0.17362	R0.39 ± 0.03785	M0.39367 ± 0.070063	R0.54467 ± 0.128183	M0.75433 ± 0.043028	R0.6 ± 0.292347
G3	M0.2257 ± 0.02742	R0.4 ± 0.27945	M0.7133 ± 0.39872	R0.216 ± 0.02179	M0.59033 ± 0.101604	R0.73167 ± 0.102485	M0.71233 ± 0.135116	R0.23433 ± 0.06331
G4	0.524 ± 0.31563		0.5973 ± 0.17472		0.246 ± 0.050507		0.246 ± 0.050507	
G5	1.127 ± 0.37599		0.3913 ± 0.10638		0.565 ± 0.213979		0.565 ± 0.213979	
G6	1.1853 ± 0.29641		0.518 ± 0.13321		0.75133 ± 0.067095		0.75133 ± 0.067095	
G7	0.2507 ± 0.03722		0.6737 ± 0.40374		0.40867 ± 0.088439		0.40867 ± 0.088439	
G8	78.0853 ± 1.99721**		80.3337 ± 5.69027**		94.52167 ± 0.618371**		94.52167 ± 0.618371**	
G9	14.3947 ± 0.54683**		26.1463 ± 2.3492**		22.854 ± 1.416826**		22.854 ± 1.416826**	
G10	9.0763 ± 1.05777**		13.6977 ± 1.19706**		8.09533 ± 1.025218**		8.09533 ± 1.025218**	
G11	M86.9087 ± 2.02258**	R89.9253 ± 1.76942**	M62.9507 ± 8.22085**	R78.0033 ± 1.35467**	M75.06 ± 3.585558**	R84.70367 ± 3.156419**	M73.30167 ± 1.11604**	R62.049 ± 4.678375**
G12	17.3627 ± 0.70493**		22.466 ± 0.64655**		18.565 ± 1.529873**		18.565 ± 1.529873**	
G13	11.147 ± 1.13543**		13.4183 ± 1.3374**		7.586 ± 0.937986*		7.586 ± 0.937986*	
G14	20.525 ± 0.87398**		28.5767 ± 0.8236**		28.693 ± 0.449418**		28.693 ± 0.449418**	
G15	7.0243 ± 1.41501*		18.3543 ± 0.78163*		11.90633 ± 1.039877**		11.90633 ± 1.039877**	

*R = 4h Compound Group, M = 4j Compound Group.

* *P*- Values between all groups = 0.000**.

*Scoring values are expressed as Mean ± Standard error; *P- Values ≤ 0.05 are significant; **P- Values ≤ 0.001 are highly significant.

The immunohistochemical results of the NFKB2 reaction of all groups in the liver and kidney were represented by Figures SI6, SI7, SI8, and SI9 and Table 3. The control groups (Gs 1, 2, and 3), **4h** (Gs 4 and 5), and **4i** treated groups (Gs 6 and 7), Figures SI6, SI7, SI8, and SI9, (A, B, and C, respectively) showed negative reactions. A strong reaction was noticed in the infected groups (Gs 8 and 11), Figures SI6, SI7, SI8, and SI9, (E and H). The reaction decreased in (Gs 9, 10, 12 to 15) but in (Gs 10, 13, and 15), Figures SI6, SI7, SI8, and SI9, (F and I) it was less than the others.

###### 2.2.2.2.3. Evaluation of immunohistochemical results "Area Percentage" (Specific area/Antibody)

Caspase 3 and NFKB2 immunostaining were measured as area % in a standard measuring frame in representative five fields for each subject (liver and kidneys) in all groups using 100x magnification via light microscopy transferred to the screen. Graphs represent the area percentage of caspase 3 and NFKB2 in liver and kidney of **4h** and **4i** treated groups, respectively, were supplied in the supplementary information (**SI2**).

Based on the above, we can conclude that *N'*-benzylidene-3,4-dimethoxybenzohydrazide candidates (**4h** and **4i**) are safe for the liver and kidney of adult male albino rats and had a promising efficacy as antimicrobial and anti-inflammatory compounds in specific doses. Further studies are needed to find out the exact mechanism of action of *N'*-benzylidene-3,4-dimethoxybenzohydrazide derivatives (**4h** and **4i**) as antimicrobial and/or anti-inflammatory as well.

### 2.3. *In silico* studies results

#### 2.3.1. Docking studies

Docking studies of the newly designed and synthesised *N'*-benzylidene-3,4-dimethoxybenzohydrazide derivatives were performed using the MOE 2019.0102 drug design software^37–39^ to propose their mechanism of action as promising antimicrobial candidates targeting the multidrug efflux pump (MATE). Targeting the MATE based on the ligand-based design of the target compounds relative to the co-crystallized native inhibitor (verapamil) of the target protein. Besides, the co-crystallized inhibitor (verapamil, 4YH) of the target protein (MATE) was inserted into the same database as a reference standard.

**Figure 7.**
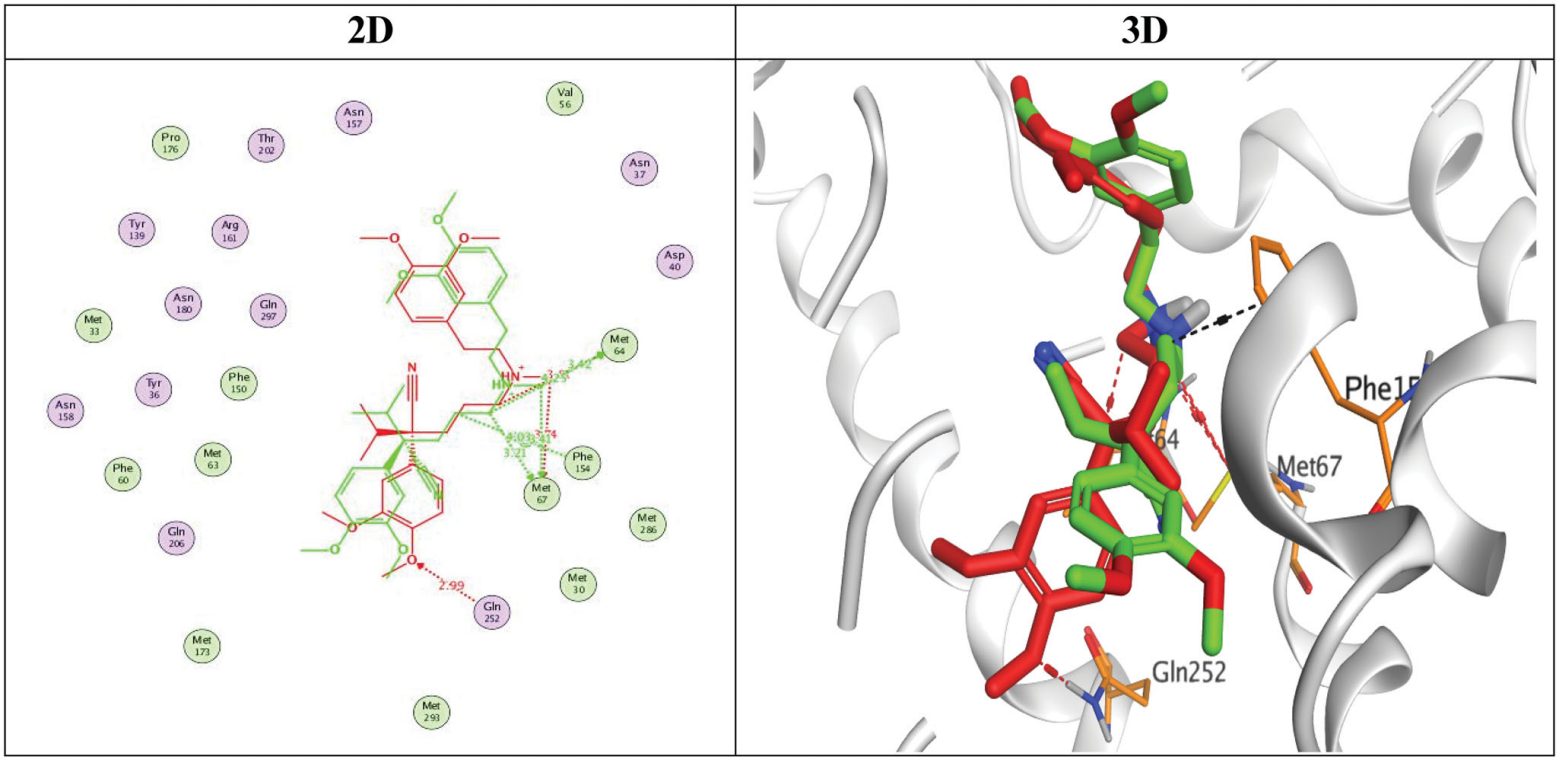
2D and 3D representations for the redocked co-crystallized 4YH antagonist inside the MATE receptor pocket.

It is worth mentioning that a validation step was carried out at the beginning of the applied MOE program by redocking the co-crystallized ligand within its binding pocket. The valid performance was approved by the low RMSD value (RMSD = 1.35) and the symmetrical superimposition in orientation between both the native (red) and redocked (green) co-crystallized poses^40–42^, Figure 7. The binding modes for compounds (**4a**, **4h**, and **4i**) as the most promising members according to their biological findings, besides the docked 4YH inhibitor were studied further as depicted in Table 4.

**Table 4.** 2D and 3D interactions for the most promising compounds (**4a**, **4h**, and **4i**) together with the docked 4YH inhibitor inside the binding pocket of the MATE receptor.

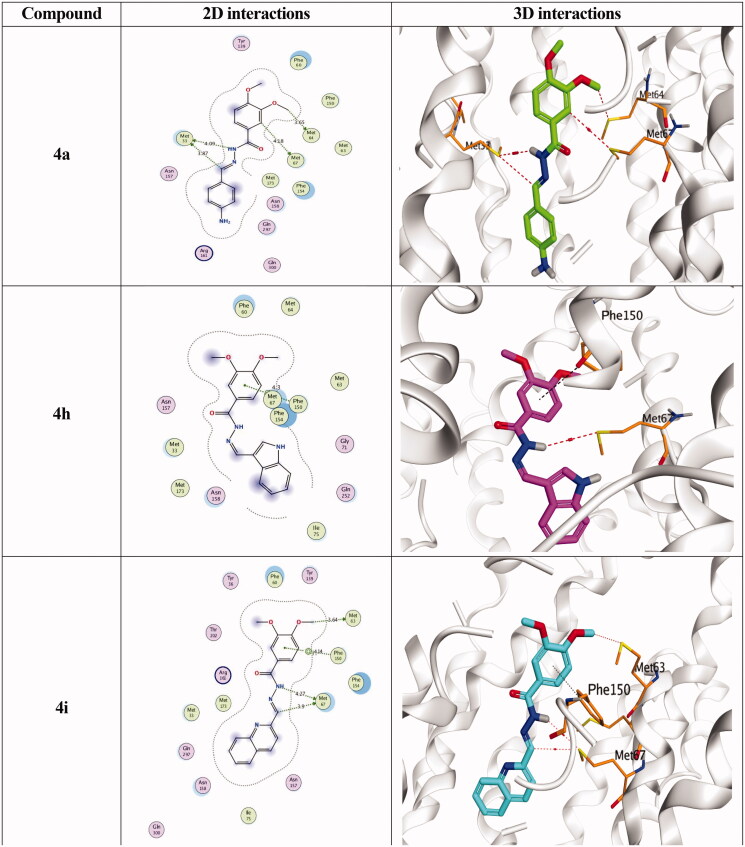
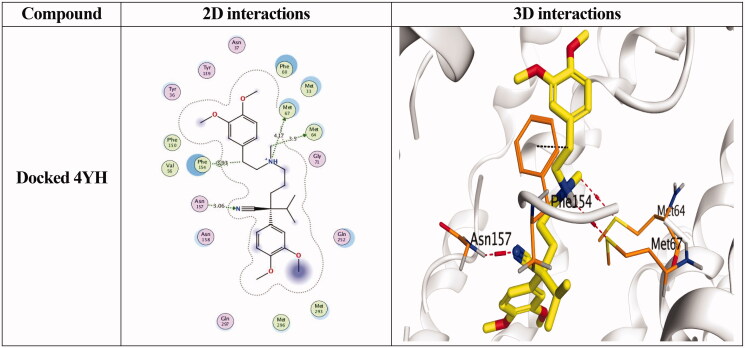

Notably, the docked 4YH inhibitor occupied the deep tube-like longitudinal pocket of the MATE receptor with approximately a similar binding mode compared to that of the co-crystallized native one, where its trimethoxyphenyl moiety occupied the largest hydrophobic region of the receptor pocket as discussed above. Besides its linker region which formed three H-bonds with Asn157, Met64, and Met67 amino acids at 3.06, 3.50, and 4.17 Å, respectively, and another H-pi bond with Phe154 amino acid at 3.93 Å as well (Table 4). Moreover, its binding score and RMSD values were found to be −9.00 kcal/mol and 1.63, respectively.

On the other hand, the binding score of compound **4a** was recorded as −6.67 kcal/mol with an RMSD value of 0.88. Its dimethoxyphenyl moiety occupied the largest hydrophobic region with the formation of two H-bonds with Met64, and Met67 amino acids at 3.65 and 4.18 Å, respectively. In addition, its linker part formed two H-bonds with Met33 amino acid at 3.87 and 4.09 Å, respectively. However, the compound **4h** binding score was −6.65 kcal/mol, and it's RMSD = 1.48. Its dimethoxyphenyl moiety formed a pi-H bond with Phe150 amino acid at 4.30 Å and its linker region formed an H-bond with Met67 amino acid at 3.81 Å. Furthermore, compound **4i** achieved a binding score of −7.07 Å and an RMSD value of 1.89. Its great stability inside the MATE binding pocket was indicated by the superior binding score noticed besides the formation of three H-bonds, one with Met63 and two with Met67 at 3.64, 3.90, and 4.27 Å, respectively. Also, an extra pi bond was formed between its dimethoxyphenyl moiety and Phe150 at 4.14 Å, Table 4.

Based on the aforementioned results we can conclude the promising binding affinities of the newly designed compounds as MATE inhibitors, which may further propose it as an expected mechanism of action for their antimicrobial activities.

#### 2.3.2. Physicochemical, ADMET, and pharmacokinetic properties prediction

The physicochemical characteristics calculations and pharmacokinetic properties for the synthesised *N'*-benzylidene-3,4-dimethoxybenzohydrazide derivatives were anticipated using the SwissADME online web tool as depicted in Table 5. Regarding their physicochemical properties, except for compound **4j**, all of the synthesised derivatives have the advantage of being either soluble or moderately soluble in water, and thus fewer issues may be experienced during drug formulations and it is recommended that any drug be absorbed has to be found in solution form at the absorption site^43^. Besides, concerning their ADME properties, except for compound **4j**, all of the synthesised derivatives attain high GIT absorption. This advantage may be assigned to their eligible lipophilicity. Therefore, they may probably have elegant bioavailabilities upon oral administration. Although compound **4j** may encounter high lipophilicity, it reveals poor GIT absorption, and that is likely owing to its high molecular weight^44^^,^^45^. Obviously, the synthesised compounds **4b**,**d**,**h**,**i** may pass the blood-brain barrier, thereby these synthesised compounds may be employed for the management of CNS microbial infections^46^. Fortunately, not all the synthesised compounds are a substrate for *P*-glycoprotein (Pgp-), so they are not amenable to the efflux mechanism used by this transporter. Besides, it is worth mentioning that compounds **4a**,**c**,**j** do not exhibit any inhibition for the common hepatic metabolising enzymes (CYP 1A2, CYP2C19, CYP2C9, CYP2D6, and CYP3A4). Furthermore, all of the synthesised derivatives do not contravene Lipinski’s rule^47^, hence their utility as good drug candidates get assurance. Besides, compounds **4a,h,i** may be utilised as lead compounds for further optimizations afterward. Furthermore, the toxicity of the newly synthesised compounds has been anticipated using the pkCSM descriptors algorithm protocol. It was revealed that, except for compound **4i**, all other synthesised compounds do not manifest Ames toxicity, thus they are not mutagenic^48^. Additionally, all of the synthesised compounds have the same advantage of being non-inhibitors of *h*ERG I, so, they do not show the cardiotoxic effect on the electrical activity of the human heart^49^. However, compounds **4e**,**f,h,i**,**j** may be considered as *h*ERG II inhibitors, hence the threat of cardiac arrhythmia may be encountered^50^. Obviously, compounds **4a**,**b**,**e**,**f** are non-hepatotoxic. Last but not the least, compounds **4e**,**f** show eligible tolerability owing to their *in silico* oral rat chronic toxicity lower values.

**Table 5.** The predicted physicochemical, and ADMET properties of synthesised compounds **4a–4j**.

	Property	Investigated compounds									
		Comp **4a**	Comp **4b**	Comp **4c**	Comp **4d**	Comp **4e**	Comp **4f**	Comp **4g**	Comp **4h**	Comp **4i**	Comp **4j**
Molecular properties	Molar Refractivity	85.00	91.89	94.91	95.49	98.02	92.64	121.16	92.45	95.90	139.96
	TPSA (A^z^ )	85.94	69.15	89.02	59.92	80.15	80.15	80.15	75.71	72.81	69.15
	Log *P* o/w (WLOGP)	2.06	2.87	2.24	3.39	3.70	3.48	4.77	2.95	3.02	6.77
	Consensus Log *P* o/w	2.07	3.00	2.18	3.56	3.46	3.27	4.66	2.65	2.78	6.51
	Water solubility	S	S	S	MS	MS	MS	MS	S	MS	PS
Pharmacokinetics parameters	GI absorption	High	High	High	High	High	High	High	High	High	Low
	BBB permeant	No	Yes	No	Yes	No	No	No	Yes	Yes	No
	*P*-gp substrate	No	No	No	No	No	No	No	No	No	No
	CYP1A2 inhibitor	No	Yes	No	Yes	Yes	Yes	No	Yes	Yes	No
	CYP2C19 inhibitor	No	Yes	No	Yes	Yes	Yes	Yes	Yes	Yes	No
	CYP2C9 inhibitor	No	Yes	No	No	Yes	Yes	No	Yes	Yes	No
	CYP2D6 inhibitor	No	Yes	No	No	No	No	No	Yes	Yes	No
	CYP3A4 inhibitor	No	No	No	Yes	Yes	No	No	Yes	Yes	No
Drug/Lead likeness	Drug likeness (Lipiniski)	Yes	Yes	Yes	Yes	Yes	Yes	Yes	Yes	Yes	Yes
	Lead likeness	Yes	No	No	No	No	No	No	Yes	Yes	No
Toxicity Parameters	Ames toxicity	No	No	No	No	No	No	No	No	Yes	No
	Max. tolerated dose (log mg/kg/day)	0.453	0.742	0.301	0.856	0.527	0.552	−0.119	0.418	0.37	0.043
	*h*ERG I inhibitor	No	No	No	No	No	No	No	No	No	No
	*h*ERG II inhibitor	No	No	No	No	Yes	Yes	No	Yes	Yes	Yes
	Oral rat acute toxicity (LD50) (mol/kg)	2.667	2.502	2.588	2.491	2.473	2.461	2.798	2.519	2.551	2.943
	Oral rat chronic toxicity (LOAEL) (log mg/kg_bw/day)	1.478	2.525	1.137	1.661	0.909	0.941	1.649	1.378	1.939	1.343
	Hepatotoxicity	No	No	Yes	Yes	No	No	Yes	Yes	Yes	Yes
	Minnow toxicity (log mM)	1.468	−0.216	1.77	0.079	0.079	0.371	−0.465	0.443	−0.112	−2.865

S: Soluble; MS: Moderately Soluble; PS: Poorly Soluble.

### 2.4. Structure- antimicrobial activity relationship (SAR) studies

Herein, we aimed to study the structure-antimicrobial activity relationship of the newly synthesised *N'*-benzylidene-3,4-dimethoxybenzohydrazide derivatives based on MIC average values^51^^,^^52^ as represented in the supplementary data (Table SI1). Thereby, the changes in their antimicrobial potential and spectrum upon structural modifications could be unveiled, thus offering clues for structural changes that are capable of increasing the antimicrobial potential.

**Figure 8.**
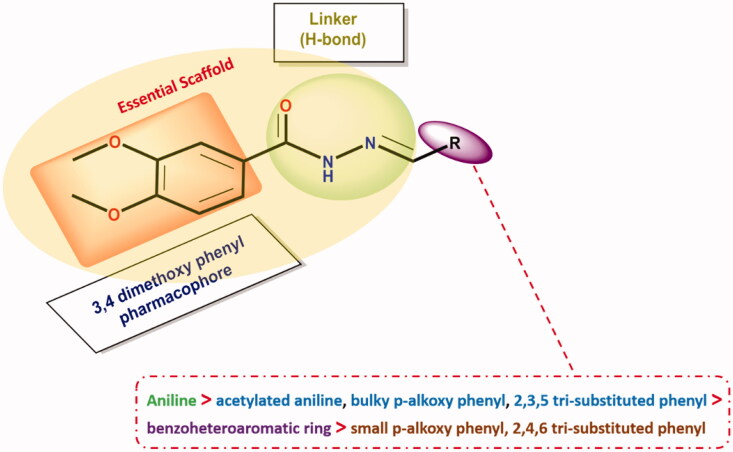
SAR of the newly investigated *N'*-benzylidene-3,4-dimethoxybenzohydrazide derivatives as potential antimicrobial inhibitors.

So, as depicted in Figure 8, analysing the structure-antimicrobial activity relationship of the newly designed and synthesised *N'*-benzylidene-3,4-dimethoxybenzohydrazide derivatives regarding their antimicrobial potential gives us the following interesting outcomes:

First, 3,4-dimethoxybenzohydrazide scaffold was revealed to be essential for exerting reliable antimicrobial activity. It is worth mentioning that the substitution of 3,4-dimethoxybenzohydrazide by aniline derivative (compound **4a**) attained high antimicrobial activity. However, it was revealed that with acylation of the –NH_2_ of aniline moiety (compound **4a**) by acetyl moiety (compound **4c**), a notable decrease in the activity with moderate antimicrobial potential was attained. Besides, it was revealed that substitution of 3,4-dimethoxybenzohydrazide by a bulky *p*-alkoxy phenyl moiety (compound **4j**), disclosed moderate antimicrobial activity. Furthermore, it was revealed that substitution by 2,3,5 tri-substituted phenyl ring (compounds **4e**, **4f**, and **4g**) displayed a moderate antimicrobial activity. Surprisingly, it was shown that with substitution of 3,4-dimethoxybenzohydrazide by benzoheteroaromatic ring (indole or quinolone) (compounds **4h** and **4i**), a remarkable decrease in the activity with weaker antimicrobial potential was attained. Finally, it was shown that the substitution of 3,4-dimethoxybenzohydrazide by a small *p*-alkoxy phenyl moiety (compound **4b**) or 2,4,6 tri-substituted phenyl ring (compound **4d**), displayed the weakest antimicrobial activity. These interesting findings can afford useful information for future work.

Moreover, based on the fact that a multiple linear regression model can be used to estimate the relationship between two or more independent variables (such as docking score and predicted Log P) and one dependent variable (average MIC values) ^53^. So, a correlation between predicted log *p* values and docking scores with MIC values (expressed in micro-molar units) using a simple multiple linear regression model was constructed as depicted in the supplementary data (Table SI2). It was estimated that *R*^2^ was 0.063. So, we can say that approximately 6.3% of the variability of MIC values can be explained by the entire set of the independent predicted Log *P* and docking scores.

## 3. Conclusion

The multidrug resistance (MDR) within bacteria and fungi arouses researchers and scientists to take leaps toward developing new efficient antimicrobial agents that are capable of conquering such microbial resistance. Herein, ten novel *N'*-benzylidene-3,4-dimethoxybenzohydrazide derivatives (**4a–j**) were designed and synthesised targeting the multidrug efflux pump (MATE). Targeting the MATE based on the ligand-based design of the target candidates relative to the co-crystallized native inhibitor (verapamil) of the target protein. The antimicrobial activity of the newly synthesised compounds in addition to reference controls was evaluated against variable microbial species. Interestingly, Compounds **4a**,**h**,**i** showed the most promising antimicrobial activities among tested compounds using the agar well diffusion assay. In addition, Compounds **4a** and **4i** showed the most relevant IC_50_ among tested compounds on *Vero* cell lines using the MTT assay. Besides, most synthesised compounds exhibited definitely feasible MIC and MBC values against the investigated microbial species. Furthermore, both compounds **4h** and **4i** containing 1*H*-indol-3-yl and quinolin-2-yl side chains, respectively, exhibited very promising antimicrobial and anti-inflammatory *in vivo* results using bio-chemical analysis and the liver and kidney histological examination. Besides, the molecular docking established assured the desirable interactions with the target protein (MATE). Additionally, the synthesised compounds can be structurally modified to attain optimised antimicrobial activity in the future. That was accomplished after shedding light on the structure-antimicrobial activity relationships (SAR). Last but not least, the ADMET *in silico* studies emphasised the practical physicochemical and pharmacokinetic properties, drug/lead likeness, and feasible toxicity parameters of the synthesised compounds.

## 4. Materials and methods

### 4.1. Chemistry

#### 4.1.1. General

All reagents and chemicals used were used without more purification. The reported yields indicate purified products. Moreover, thin-layer chromatography (TLC) Merck Silica Gel 60 F254 was used to follow all reactions, and also it was visualised using a UV lamp. Melting points were determined on a BUCHI melting point M-565 apparatus. An FT-IT PerkinElmer Spectrum GX was used to record the IR spectrum. A JOEL-ECA 600 MHz was utilised to record the NMR spectra in DMSO-*d6* relative to the tetramethylsilane at 600 MHz for 1H and 150 MHz for 13 C measurements and Elemental analysis was performed by Microanalytical Department at the National Centre for Chemical Technologies at King Abdulaziz City for Science and Technology (Riyadh, Saudi Arabia). The chemical shifts were expressed in *δ*-values (ppm) relative to TMS. The coupling constants (*J*) were expressed in Hz. D_2_O was added to confirm the exchangeable protons. The mass spectrum was determined on a GCMS JEOL JMS-Q1050GC Ultra Quad GC/MS (70 eV).

Methyl 3,4-dimethoxybenzoate (**2**) (50 mmol) was refluxed after mixing with hydrazine hydrate (40 ml) in ethanol for 8 h to give us 3,4-dimethoxybenzohydrazide **(3)** as an intermediate step. Then, compound (**3**) (1 mmol) was further refluxed (6 h) with different substituted aldehydes (1 mmol) in ethanol/DMF to give us the desired 3,4-dimethoxybenzohydrazones **(4a–4j)**. The reaction proceeding was followed by TLC. Structures of all the newly synthesised candidates were confirmed using different spectroscopic techniques including IR, ^1^H NMR, ^13 ^C NMR, and MS.

##### N'-(4-aminobenzylidene)-3,4-dimethoxybenzohydrazide (4a)

**Off-white** powder; **Yield**: (83%); **m.p.**: 180–182 °C. **IR**: υ/cm^−1^= 3370, 3279 (NH_2_, NH), 3014 (Ar-H), 2955, 2857 (aliph-H), 1660 (C = O amide), 1612 (C = N). **^1^H NMR** (600 MHz) δ/ppm= 3.37,3.45 (2 s, 6H, OCH_3_), 5.45 (s, 2H, NH_2_ exchangeable by D_2_O), 6.54 (d, 1H, Ar-H, *J* = 6.5 Hz), 7.03 (d, 2H, Ar-H), 7.36–7.50 (m, 2H, Ar-H), 8.20 (d, 2H, Ar-H), 8.36 (s, 1H, CH = N), 11.31 (s, 1H, NH exchangeable by D_2_O). **^13 ^C NMR**: 56.12, 56.22 (2 OCH_3_), 111.29, 114.09, 121.25, 122.00, 126.34, 129.11, 130.24, 148.55, 151.04, 152.43 (Ar-C + C=N), 160.28 (C = O). **Mass spectrum** demonstrated a molecular ion peak at *m/z*: 299 (M**^+^**^.^, 12.08%) with a base peak at *m/z*: 98. **Anal. Calc**. for C_16_H_17_N_3_O_3_ (299.33): C, 64.20; H, 5.72; N, 14.04. Found: C, 64.19; H, 5.73; N, 13.99.

##### N'-(4-ethoxybenzylidene)-3,4-dimethoxybenzohydrazide (4 b)

**Grey** powder; **Yield**: (53%); **m.p.**: 171–173 °C. **IR**: υ/cm^−1^= 3294 (NH), 3009 (Ar-H), 2990, 2946 (aliph-H), 1660 (C = O amide), 1619 (C = N). **^1^H NMR** (600 MHz) δ/ppm= 1.30 (t, 3H, CH_3_), 3.79 (s, 6H, OCH_3_), 4.05 (q, 2H, CH_2_), 6.96 (d, 1H, Ar-H, *J* = 7.2 Hz), 7.03 (d, 2H, Ar-H), 7.44–7.61 (m, 2H, Ar-H), 8.20 (d, 2H, Ar-H), 8.35 (s, 1H, CH = N), 11.53 (s, 1H, NH exchangeable by D_2_O). **^13 ^C NMR**: 15.08 (CH_3_), 56.16 (2 OCH_3_), 63.80 (CH_2_), 111.18, 111.68, 115.26, 121.44, 127.25, 118.14, 129.26, 131.39, 147.90, 148.83, 152.18 (Ar-C + C=N), 160.60 (C = O). **Mass spectrum** demonstrated a molecular ion peak at *m/z*: 328 (M**^+^**^.^, 15.08%) with a base peak at *m/z*: 56. **Anal. Calc**. for C_18_H_20_N_2_O_4_ (328.37): C, 65.84; H, 6.14; N, 8.53. Found: C, 65.72; H, 6.36; N, 8.21.

##### N-(4-((2–(3,4-dimethoxybenzoyl)hydrazono)methyl)phenyl)acetamide (4c)

**Creamy** powder; **Yield**: (68%); **m.p.**: 155–157 °C. **IR**: υ/cm^−1^= 3369,3257 (2NH), 3013 (Ar-H), 2919, 2872 (aliph-H), 1658 (br-2 C = O amide), 1612 (C = N). **^1^H NMR** (600 MHz) δ/ppm= 2.02 (s, 3H, CH_3_), 3.78 (s, 6H, OCH_3_), 6.99 (d, 1H, Ar-H, *J* = 6.9 Hz), 7.04 (d, 2H, Ar-H), 7.43–7.62 (m, 2H, Ar-H), 8.33 (d, 2H, Ar-H), 8.56 (s, 1H, CH = N), 10.52, 10.93 (2 s, 2H, 2NH exchangeable by D_2_O).**^13^C NMR**: 24.50 (CH_3_), 56.16 (2 OCH_3_), 111.22, 119.42, 121.48, 125.81, 128.32, 129.54, 141.23, 147.68, 148.84, 149.10, 152.22 (Ar-C + C=N), 163.10, 169.26 (2 C = O). **Mass spectrum** demonstrated a molecular ion peak at *m/z*: 341 (M**^+^**^.^, 10.08%) with a base peak at *m/z*: 224. **Anal. Calc**. for C_18_H_19_N_3_O_4_ (341.37): C, 63.33; H, 5.61; N, 12.31. Found: C, 63.34; H, 5.42; N, 12.52.

##### N'-(2,4,6-trimethylbenzylidene)-3,4-dimethoxybenzohydrazide (4d)

**Off White** solid; **Yield**: (89%); **m.p.**: 191–193 °C. **IR**: υ/cm^−1^= 3220 (NH), 3047 (Ar-H), 2919, 2850 (aliph-H), 1652 (C = O amide). **^1^H NMR** (600 MHz) δ/ppm = 2.48, 2.56 (2 s, 9H, 3CH_3_), 3.80 (s, 6H, 2OCH_3_), 7.07 (s, 2H, Ar-H), 7.17 (d, 1H, Ar-H-5, *J* = 7.1 Hz), 7.38 (s, 1H, Ar-H), 7.46 (d, 1H, Ar-H-6, *J* = 7.8 Hz), 8.52 (s, 1H, CH = N), 11.50 (s, 1H, NH exchangeable by D_2_O). **^13 ^C NMR**: 29.80, 31.73 (3 CH_3_), 56.12, 56.22 (2 OCH_3_), 111.25, 111.62, 117.54, 121.64, 125.04, 126.02, 126.22, 136.09, 141.02, 148.95, 151.23, 152.52, 154.89 (Ar-C + C=N), 162.75 (C = O). **Mass spectrum** demonstrated a molecular ion peak at *m/z*: 326 (M**^+^**^.^, 27.06%) with a base peak at *m/z*: 44. **Anal. Calc**. for C_19_H_22_N_2_O_3_ (326.40) C, 69.92; H, 6.79; N, 8.58. Found: C, 70.11; H, 6.80; N, 8.73.

##### N'-(3,5-dibromo-2-hydroxybenzylidene)-3,4-dimethoxybenzohydrazide (4e)

**Yellow crystal**; **Yield**: (88%); **m.p.**: 206–208 °C. **IR**: υ/cm^−1^= 3403–3164 (br-OH&NH), 3083 (Ar-H), 2919, 2850 (aliph-H), 1662 (C = O amide), 1624 (C = N). **^1^H NMR** (600 MHz) δ/ppm= 3.79 (s, 6H, 2OCH_3_), 7.05, 7.20 (2 s, 2H, Ar-H), 7.45 (d, 1H, Ar-H-5, *J* = 7.1 Hz), 7.54 (s, 1H, Ar-H),7.69 (d, 1H, Ar-H, *J* = 7.5 Hz), 8.45 (s, 1H, CH = N), 10.02, 10.50 (2 s, 2H, NH & OH exchangeable by D_2_O). **^13 ^C NMR**: 56.11, 56.21 (2 OCH_3_), 111.24, 111.58, 121.89, 121.50, 121.87, 121.89, 132.50, 136.01, 142.37, 147.05, 148.95, 152.74, 153.23, 156.68 (Ar-C + C=N), 162.92 (C = O). **Mass spectrum** demonstrated a molecular ion peak at *m/z*: 458 (M**^+^**^.^, 30.15%) 460 (M **^+^**^2^, 27.08%), 462 (M ^+ 4^, 27.22%) with a base peak at *m/z*: 44. **Anal. Calc**. for C_16_H_14_Br_2_N_2_O_4_ (458.11): C, 41.95; H, 3.08; N, 6.12. Found: C, 42.01; H, 3.09; N, 6.34.

##### N'-(3,5-dichloro-2-hydroxybenzylidene)-3,4-dimethoxybenzohydrazide (4f)

**Bright brown powder; Yield:** (72%); **m.p.:** 180–182 °C. **IR:** υ/cm^−1^= 3375, 3219 (OH&NH), 3027 (Ar-H), 2958, 2853 (aliph-H), 1654 (C = O amide), 1608 (C = N). **^1^H NMR** (600 MHz) δ/ppm= 3.95, 3.97 (2 s, 6H, 2OCH_3_), 7.15,7.16 (2 s, 2H, Ar-H), 7.58–7.61 (m, 3H, Ar-H), 8.41 (s, 1H, CH = N), 9.79, 10.50 (2 s, 2H, NH&OH exchangeable by D_2_O). **^13 ^C NMR**: 55.97, 56.04 (2 OCH_3_), 110.63, 114.81, 115.67, 119.91, 120.70, 124.39, 125.28, 125.80, 148.67, 148.77, 151.60 (Ar-C + C=N), 166.29 (C = O). **Mass spectrum** demonstrated a molecular ion peak at *m/z*: 369 (M**^+^**^.^, 18.32%), 371 (M ^+ 2^, 6.13%) and 373 (M ^+ 4^, 18.33%) with a base peak at *m/z*: 42. **Anal. Calc**. for C_16_H_14_Cl_2_N_2_O_4_ (369.20): C, 52.05; H, 3.82; N, 7.59. Found: C, 52.03; H, 3.59; N, 7.73.

##### N'-(3,5-di-tert-butyl-2-hydroxybenzylidene)-3,4-dimethoxybenzohydrazide (4g)

**Shiny yellow crystals; Yield:** (85%); **m.p.:** 235–237 °C**. IR**: υ/cm^−1^= 3248, 3127 (OH&NH), 3022 (Ar-H), 2918, 2885 (aliph-H), 1655 (C = O amide), 1604 (C = N). **^1^H NMR** (600 MHz) δ/ppm= 1.47, 1.59 (2 s,18H,6CH_3_), 3.99, 4.0 (2 s, 6H, 2OCH_3_), 7.30 (s, 1H, Ar-H), 7.39 (s, 1H, Ar-H), 7.49 (s, 1H, Ar-H), 7.74 (d, 1H, Ar-H, *J* = 6.5 Hz), 7.66 (d, 1H, Ar-H, *J* = 6.5 Hz), 8.75 (s, 1H, CH = N), 9.27, 12.22 (2 s, 2H, NH&OH exchangeable by D_2_O). **^13 ^C NMR**: 30.01, 31.64 (6CH_3_), 34.23, 35.03(C-C), 56.03, 56.36 (2 OCH_3_), 111.44, 117.68, 121.63, 124.84, 125.97, 136.15, 140.74, 148.66, 150.92, 152.66, 155.18 (Ar-C + C=N), 162.83 (C = O). **Mass spectrum** demonstrated a molecular ion peak at *m/z*: 412 (M**^+^**^.^, 19.53%) with a base peak at *m/z*: 260. **Anal. Calc**. for C_24_H_32_N_2_O_4_ (412.53): C, 69.88; H, 7.82; N, 6.79. Found: C, 69.81; H, 7.93; N, 7.09.

##### N'-((3a,7a-dihydro-1H-indol-3-yl)methylene)-3,4-dimethoxybenzohydrazide (4h)

**Deep violet crystals; Yield:** (92%); **m.p.:** 210–212 °C**. IR**: υ/cm^−1^= 3305 (NH), 3033 (Ar-H), 2978, 2851 (aliph-H), 1686 (C = O amide), 1617 (C = N). **^1^H NMR** (600 MHz) δ/ppm= 3.97, 3.99 (2 s, 6H, 2OCH_3_), 7.30 (d, 1H, Ar-H, *J* = 6.8 Hz), 7.66–7.97 (m, 9H, Ar-H), 8.45 (s, 1H, CH = N), 11.54, 11.74 (2 s, 2H, 2NH exchangeable by D_2_O). **^13 ^C NMR**: 56.12, 56.16 (2 OCH3), 111.50, 112.32, 120.86, 121.06, 122.54, 123.12, 123.49, 124.88, 126.65, 132.39, 137.54, 145.04, 146.99, 148.82, 155.63 (Ar-C + C=N), 162.51 (C = O). **Mass spectrum** demonstrated a molecular ion peak at *m/z*: 325 (M**^+^**^.^, 21.33%) with a base peak at *m/z*: 67. **Anal. Calc**. for C_18_H_19_N_3_O_3_ (325.37): C, 66.45; H, 5.89; N, 12.91. Found: C, 66.55; H, 6.01; N, 13.21.

##### N'-(quinolin-2-ylmethylene)-3,4-dimethoxybenzohydrazide (4i)

**Yellowish white powder**; **Yield**: (69%); **m.p.**: 280–282 °C. **IR**: υ/cm^−1^= 3167(NH), 3023 (Ar-H), 2913, 2851 (aliph-H), 1651 (C = O amide), 1614 (C = N). **^1^H NMR** (600 MHz,) δ/ppm= 3.97 (s, 6H, 2OCH_3_), 7.27 (s, 1H, CH = N), 7.68 (s, 1H, Ar-H), 7.76–7.96 (m, 4H, Ar-H), 8.17 (d, 1H, Ar-H, *J* = 7.5 Hz), 8.20 (d, 1H, Ar-H, *J* = 6.8 Hz), 8.56 (d, 1H, Ar-H, *J* = 6.5 Hz), 8.78 (s, 1H, Ar-H), 12.21 (s, 1H, NH exchangeable by D_2_O). **^13 ^C NMR**: 56.19, 56.25 (2 OCH_3_), 111.56, 118.03, 121.73, 125.69, 127.83, 128.42, 128.57, 129.44, 130.63, 137.29, 147.91, 148.86, 149.73, 151.94, 152.21, 154.45 (Ar-C + C=N), 163.43 (C = O). **Mass spectrum** demonstrated a molecular ion peak at *m/z*: 335 (M**^+^**^.^, 8.12%) with a base peak at *m/z*: 148. **Anal. Calc**. for C_19_H_17_N_3_O_3_ (335.36): C, 68.05; H, 5.11; N, 12.53. Found: C, 68.31; H, 4.91; N, 12.21.

##### N'-(4-(dodecyloxy)benzylidene)-3,4-dimethoxybenzohydrazide (4j)

**Greyish white powder; Yield:** (77%); **m.p.:** 188–190 °C**. IR**: υ/cm^−1^= 3277 (NH), 3054 (Ar-H), 2945, 2865 (aliph-H), 1661 (C = O amide), 1616 (C = N). **^1^H NMR** (600 MHz) δ/ppm= 0.85(t, 3H, CH_3_), 1.49 (m,20H,10CH_2_), 3.95, 3.97 (2 s, 6H, 2OCH_3_), 4.23(t, 2H, OCH_2_), 7.16 (d, 2H, Ar-H, *J* = 6.5 Hz), 7.64 − 7.81 (m, 5H, Ar-H), 8.55 (s, 1H, CH = N), 11.73 (s, 1H, NH exchangeable by D_2_O). **^13 ^C NMR**: 14.41 (CH_3_),22.63, 25.63, 29.52, 31.86 (CH_2_), 56.83 (2 OCH_3_), 68.28(OCH_2_), 111.29, 115.09, 121.00, 125.79, 126.04, 127.26, 128.76, 147.41, 152.15 (Ar-C + C=N),160.73 (C-O), 162.82 (C = O). **Mass spectrum** demonstrated a molecular ion peak at *m/z*: 468 (M**^+^**^.^, 15.32%) with a base peak at *m/z*: 352. **Anal. Calc**. for C_28_H_40_N_2_O_4_ (468.64): C, 71.76; H, 8.60; N, 5.98. **Found:** C, 71.77; H, 8.62; N, 6.23.

### 4.2. Biological evaluation

#### 4.2.1. *In vitro* antibacterial and antifungal activity testing

All compounds were tested for their antimicrobial activity on different bacterial groups (gram-positive and gram-negative bacteria) and fungi (*C. albicans*). All compounds were tested for their safety on tissue culture cells by cytotoxicity assay. All experiments were done according to Clinical and Laboratory Standards Institute guidelines^54^.

**a.  Antibacterial activity**

*In vitro* evaluation of the target compounds (**4a–j**) in addition to reference, antibiotics were performed regarding their antibacterial activities using zone inhibition technique (well agar diffusion method). Besides, minimal inhibitory concentration (MIC) utilising gam-positive bacteria such as *S. aureus* ATCC 6538, *E. faecalis* ATCC 29212, gram-negative bacteria such as *P. aeruginosa* ATCC10145, *E. coli* ATCC 25922, *A. baumanii* ATCC® 19606, *A. baumanii* clinical isolate, multidrug-resistant *A. baumanii* clinical isolate, and *S. typhi* clinical isolate.

**b.  Antifungal activity**

*In vitro* evaluation for antifungal activities regarding target compounds (**4a–j**) as well as reference drug, was done against *C. albicans MTCC183* through the zone inhibition technique using well diffusion agar assay in addition to MIC.

##### 4.2.1.1. Well diffusion assay

A modification of the agar well diffusion method^55^ was done to detect the antimicrobial activities of different compounds (**4a–j**) against gram-positive bacteria, gram-negative bacteria, and fungi. The work was carried out at the Microbiology Department Faculty of Medicine for Girls Al-Azhar University. By visual comparison to 0.5 McFarland turbidity standards, a suspension of each bacterial strain was prepared^56^. The zones of inhibition were measured in cm. The results indicated that the compounds exhibited antibacterial activities against some of the tested microorganisms.

##### 4.2.1.2. Minimal inhibitory concentration (MIC)

In the broth dilution method, different concentrations of an antimicrobial agent are added to the microorganisms that are inoculated into a liquid growth medium. After overnight incubation, the microbial growth is assessed through spectrophotometric cell counts. The MIC value is the lowest concentration of the tested compound which suppresses the growth of the microorganism^57^^,^^58^.

Two folds serial dilutions of the tested compounds were prepared and quality control (QC) antibiotics/antifungal (vancomycin, ceftriaxone, and amphotericin B) were prepared in a 96 wells microdilution plate started with 1000 µg/ml, 500 µg/ml, 250 µg/ml, 125 µg/ml, 62.5 µg/ml, and 31.25 µg/ml until reach 15.6 µg/ml, 7.81 µg/ml, 3.9 µg/ml, 1.95 µg/ml, and 0.97 µg/ml, 0.49 µg/ml.

Suspensions from the tested bacteria/fungus were prepared by making overnight broth culture of the tested organism then adjusted to half McFarland, (With Optical Density 0.1 at wavelength 580 nm). The tested bacteria were inoculated into a microdilution plate with the serially diluted test chemical compounds and incubated overnight. The microdilution plate is read after 24 h to determine the MIC value.

##### 4.2.1.3. Minimal bactericidal concentration determination (MBC)

After determining the MIC for the compounds, the suspension from the upper four concentrations higher than the MIC level for each compound was inoculated onto an appropriate agar media, then incubated for 24 h at 37 °C and each agar plate was checked for bacterial growth to determine the MBC.

##### 4.2.1.4. Determination of sample cytotoxicity on cells using MTT assay

The MTT (3–(4,5-dimethylthiazol-2-yl)-2,5-diphenyltetrazolium bromide) tetrazolium reduction assay protocol ^59^ was applied to determine the cytotoxicity of the target compounds (**4a–j**) on cells.

#### 4.2.2. *In vivo* testing

##### 4.2.2.1. Materials and methods

###### 4.2.2.1.1. Drugs

The compounds **4h** and **4j** were dissolved in DMSO (dimethyl sulfoxide) then further dilutions were made by using sterile distilled water. The doses of both candidates were calculated according to the method given by Paget and Barnes^60^. Sterile distilled water, DEMSO, and investigated compounds (**4h** (1.9 and 3.8 mg/kg) and **4j** (1.9 and 3.8 mg/kg)) were injected in a volume of 4 ml/kg^61^. The experiment lasts two weeks, the 1^st^ week for induction of infection in the infected groups and the 2^nd^ week for the administration of the compounds in all groups.

###### 4.2.2.1.2. Animals

One hundred thirty-five adult male 8-week-old Westar albino rats (RRID: RGD_13508588) (200 ± 20 g) were purchased from National Research Centre (NRC Cairo, Egypt). They were kept for a week before any intervention to accommodate the experimental conditions at the animal house in the NRC, in standard polypropylene cages (three rats per cage). Also, they were allowed to access freshwater *ad libitum* and standard rodent food pellets freely (El-Nasr Company, Abou- Zaabal -Egypt). The pellets consisted of 5% fibres, 3.5% fat, 6.5% ash, and 20% proteins all over the experimental duration. The rats were kept under standard temperature (22 ± 2 °C) and relative humidity (55 ± 5%) conditions with a 12-light/12-dark cycle.

###### 4.2.2.1.3. Ethics statement

All experimental procedures were performed following the ARRIVE guidelines^62^ and according to the U.K. Animals (Scientific Procedures) Act, 1986, the Guide for the Care and Use of Laboratory Animals^63^, and the ethical procedures and policies approved by the Animal Care and Use Committee of the National Research Centre, Cairo, Egypt (1077/3–11-2021). All experiments were performed under blinded conditions. All measures were taken to decrease the number of used animals and lessen their suffering.

###### 4.2.2.1.4. Experimental design

Rats were divided randomly into equal fifteen groups, each containing 9 rats as follows:

- **Group 1**: Rats were kept on standard laboratory chow and water ad libitum to serve as a control.
- **Group 2:** Rats have injected IP with sterile distilled water (4ml/kg) daily for one week to serve as a control.
- **Group 3:** Rats have injected IP with DEMSO (dimethyl sulfoxide) (4 ml/kg) daily for one week to serve as a control.
- **Group 4:** Rats have injected IP with **4h** (1.9 mg/kg**)** daily for one week.
- **Group 5:** Rats have injected IP with **4h** (3.8 mg/kg) daily for one week.
- **Group 6:** Rats have injected IP with **4i** (1.9 mg/kg**)** daily for one week.
- **Group 7:** Rats have injected IP with **4i** (3.8 mg/kg**)** daily for one week.
- **Group 8:** Rats were infected by a single IM injection of *S. aureus* strain [1.0 ml) 1 × 10^7^) colony-forming units].
- **Group 9:** Rats have injected IP with **4h** (1.9 mg/kg**)** daily for one week on the 7^th^ day after induction of infection by a single IM injection of *S. aureus* strain [1.0 ml (1 × 10^7^) colony-forming units.
- **Group 10:** Rats have injected IP with **4h** (3.8 mg/kg**)** daily for one week on the 7^th^ day after induction of infection by a single IM injection of *S. aureus* strain [1.0 ml (1 × 10^7^) colony-forming units].
- **Group 11:** Rats were infected by a single IM injection of *S. typhi* strain [1.0 ml (1 × 10^7^) colony forming units).
- **Group 12:** Rats have injected IP with **4h** (1.9 mg/kg**)** daily for one week. on the 7^th^ day after induction of infection by a single IM injection of *S. typhi* strain [1.0 ml (1 × 10^7^) colony-forming units].
- **Group 13:** Rats have injected IP with **4h** (3.8 mg/kg**)** daily for one week on the 7^th^ day after infection by a single IM injection of *S. typhi* strain [1.0 ml (1 × 10^7^) colony-forming units].
- **Group 14**: Rats have injected IP with **4i** (1.9 mg/kg**)** daily for one week. on the 7^th^ day after induction of infection by a single IM injection of *S. typhi* strain [1.0 ml (1 × 10^7^) colony-forming units].
- **Group 15:** Rats have injected IP with **4i** (3.8 mg/kg**)** daily for one week. on the 7^th^ day after induction of infection by a single IM injection of Salmonella strain [1.0 ml (1 × 10^7^) colony-forming units].

The induced infection in the rats injected by *S. aureus* and *S. typhi* strains was confirmed by an increased serum level of C reactive protein (a surrogate marker of infection) on the 8^th^ day of induction.

In the end, blood was collected from the retro-orbital venous plexus of rats in all groups by heparinised capillary tubes under ether anaesthesia^64^. The blood samples were collected in centrifuge tubes and allowed to clot at room temperature for an hour, and then centrifuged for 15 min at 3000 rpm. Sera were sent for lab analysis immediately for detection of biochemical markers of inflammation, assess host immunity, oxidative stress markers, in addition to body system assessment (liver function test and kidney function test). Finally, anaesthetised rats were sacrificed through cervical dislocation^65^ then liver and kidney were dissected for histological study.

##### 4.2.2.2. Biochemical analysis

###### 4.2.2.2.1. Assessment of liver function

ALT and AST were determined^66^ using commercial kits obtained from Diamond Diagnostics, Egypt.

###### 4.2.2.2.2. Assessment of kidney function

Serum urea was determined by using the modified Berthelot–Searcy method^67^. Serum creatinine activity was also assessed by using the application of Jaffe’s reaction^68^.

###### 4.2.2.2.3. Assessment of host immunity

Serum levels of the pro-inflammatory cytokine, TNF-α, were determined by the quantitative enzyme immunoassay (EIA) kit purchased from R&D Systems (USA) according to the method of Howard and Harada^69^, while that of anti-inflammatory cytokine IL-10 was according to Croft *et al.* ^70^.

###### 4.2.2.2.4. Assessment of redox state

The method of Sedlak and Lindsay ^71^ was adopted for the colorimetric assessment of serum malondialdehyde (MDA) using free-SH groups for measuring the peroxidation of fatty acids as an oxidative stress marker. While “master antioxidant” serum glutathione (GSH) was assessed using Cayman's GSH assay kit^72^.

###### 4.2.2.2.5. Statistical Analysis

Statistical Analysis of results was done using the statistic package for social science version 12 (SPSS, 12) for windows. Results were expressed as Mean ± Standard deviation (SD) and statistically analysed using one-way analysis of variance (ANOVA) for a completely.

##### 4.2.2.3. Histological examination of the liver and kidney

###### 4.2.2.3.1. General histological examination

At the end of the experiment, the animals were euthanized through cervical dislocation. After that, the liver and kidney were dissected for histological study. The procedure for histological preparations was described by Bancroft and Stevens^73^. Briefly, liver and kidney tissues were sliced to 3–4 mm thick, fixed in 10% neutral buffered formalin (10% NBF), dehydrated in graded concentrations of ethanol, cleared in xylene, and embedded in paraffin. The paraffin blocks were sectioned with a microtome at (4–6 μm) thickness and dyed with H & E stain to study the general tissue structure.

###### 4.2.2.3.2. Immunohistochemistry staining protocol

Immunohistochemistry (IHC) was performed on paraffin sections and mounted on positively charged slides to detect caspase-3 as an indicator for apoptosis and nuclear factor kappa-light-chain-enhancer of activated B cells (NFKB2) for inflammation by using the avidin-biotin-peroxidase complex (ABC) method^74^. The exact methodology was carried out as previously described in detail^75^.

###### 4.2.2.3.3. Evaluation of immunohistochemical results "Area Percentage" (Specific area/Antibody)

Caspase-3 and NFKB2 immunostaining were measured as area % in a standard measuring frame in representative five fields for each subject (liver and kidneys) in all groups using 100x magnification via light microscopy transferred to the screen^75^.

###### 4.2.2.3.4. Statistical analysis of area percentage of caspase 3 and NFKB2

Values related to area percentage were given as Mean and Standard Error "SE". Data were tested for normality via Kolmogorov-Smirnov test of normality. The outcomes of the Kolmogorov-Smirnov test directed that the greatest of data were normally distributed (parametric data), thus one way ANOVA test was applied for intergroup comparison^76^ followed by the Post-hoc Tukey’s test. *P* values less than 0.05 were interpreted as statistically significant^77^.

### 4.3. *In silico* studies

#### 4.3.1. Docking studies

Molecular docking studies of the newly designed *N'*-benzylidene-3,4-dimethoxybenzohydrazide derivatives towards the multidrug efflux pump (MATE) protein receptor (PDB ID: 5C6O)^25^ were carried out using the MOE 2019.0102 drug design software^78–80^. Targeting the MATE based on the ligand-based design approach followed to obtain the target compounds relative to the co-crystallized native inhibitor (verapamil) of the target MATE protein. Also, the co-crystallized inhibitor (verapamil, 4YH) of MATE was inserted into the docked database as a reference standard.

First, all of the newly designed *N'*-benzylidene-3,4-dimethoxybenzohydrazide derivatives were prepared and inserted into one database with the previously mentioned co-crystallized inhibitor (4YH) as previously mentioned^81–85^. Then, the target protein (MATE) was prepared for the docking process following the default steps described earlier^86–90^. Moreover, a validation process for the docking program was performed by applying a separate docking process for the co-crystallized inhibitor alone (redocking)^91–95^. Finally, a general docking process was applied according to the default methodology^96–100^ and the best poses -regarding their binding scores and modes- were selected for further studies^99^^,^^101–103^.

#### 4.3.2. Physicochemical, ADMET, and pharmacokinetic properties prediction

The physicochemical and pharmacokinetic characteristics investigation is a critical step in the synthesis of novel molecular entities from a hit to a drug^18^^,^^104–107^. Thereby, the Swiss Institute of Bioinformatics (SIB) supplies the free Swiss ADME web tool which can be used to evaluate physicochemical characteristics, and anticipate the ADME parameters and pharmacokinetic features of the newly synthesised compounds as well. Structures of the chemically synthesised compounds were transformed to SMILES notations, then submitted to the online server for further calculations running^108^. Moreover, the synthesised compounds' toxicity parameters were investigated using the pkCSM descriptors algorithm protocol^109^.

## Supplementary Material

Supplemental Material
