# Supplementary material for: Ligand-based design and synthesis of N'-Benzylidene-3,4-dimethoxybenzohydrazide derivatives as potential antimicrobial agents; evaluation by in vitro, in vivo, and in silico approaches with SAR studies
Source: J Enzyme Inhib Med Chem. 2022 Apr 18;37(1):1098–119. doi: 10.1080/14756366.2022.2063282 (PMC9037180; doi:10.1080/14756366.2022.2063282)

Ligand-Based Design and Synthesis of *N'*-Benzylidene-3,4-dimethoxybenzohydrazide Derivatives as Potential Antimicrobial Agents; Evaluation by *In Vitro, In Vivo, and In Silico* Approaches with SAR Studies

Rogy R. Ezz Eldin[1,Ψ], Marwa A. Saleh[2,Ψ,*], Mohammad Hayal Alotaibi[3], Reem K. Alsuair[3], Yahya A. Alzahrani[3], Feras A. Alshehri[3], Amany F. Mohamed[4], Shaimaa M. Hafez[4], Azza Ali Althoqapy[5], Seham K. Khirala[5], Mona M. Amin[6], Yousuf A. F.[7], Azza H.AbdElwahab[7], Mohamed S. Alesawy[8], Ayman Abo Elmaaty[9], and Ahmed A. Al-karmalawy[10,*]

[1] Pharmaceutical Organic Chemistry Department, Faculty of Pharmacy, Port Said University, Port Said, Egypt.

[2] Pharmaceutical Organic Chemistry Department, Faculty of Pharmacy (Girls), Al-Azhar University, Cairo, Egypt.

[3] National Center for Chemical Technologies, King Abdulaziz City for Science and Technology, P. O. Box 6086, Riyadh 11442, Saudi Arabia.

[4] Department of Anatomy, Faculty of Medicine for Girls, Al-Azhar University, Cairo, Egypt.

[5] Department of Microbiology and Immunology, Faculty of Medicine for Girls, Al-Azhar University, Cairo, Egypt.

[6] Department of Pharmacology, Faculty of Medicine for Girls, Al-Azhar University, Cairo, Egypt.

[7] Department of Physiology, Faculty of Medicine for Girls, Al-Azhar University, Cairo, Egypt.

[8] Pharmaceutical Medicinal Chemistry and Drug Design Department, Faculty of Pharmacy (Boys), Al-Azhar University, Cairo, Egypt.

[9] Department of Medicinal Chemistry, Faculty of Pharmacy, Port Said University, Port Said 42526, Egypt.

[10] Department of Pharmaceutical Medicinal Chemistry, Faculty of Pharmacy, Horus University-Egypt, New Damietta 34518, Egypt.

* Corresponding authors:

Ahmed A. Al-Karmalawy: Email: akarmalawy@horus.edu.eg

ORCID: 0000-0002-8173-6073

Marwa A. Saleh: Email: marwasaleh577@yahoo.com

ORCID: 0000-0003-0767-9864

Ψ These authors equally contributed to this work.

Supplementary Information

Figure SI1. Antibacterial activities of some tested compounds (**4a**, **4b**, **4h**, and **4e**) against gram-positive and gram-negative bacteria by agar well diffusion assay.

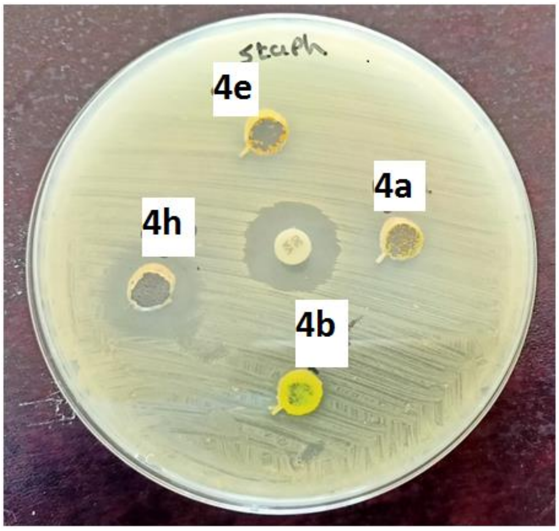	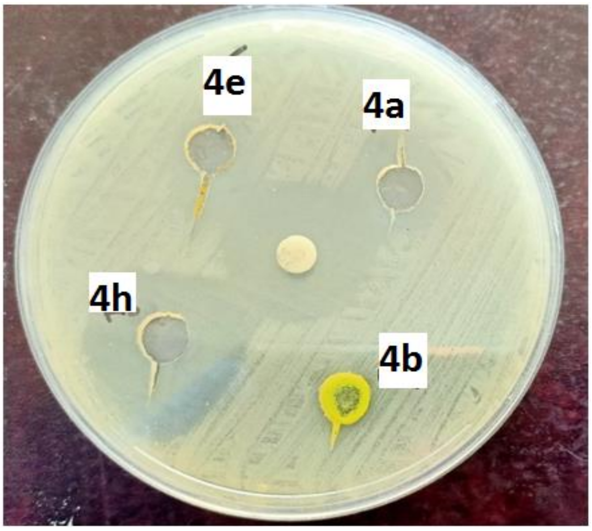
(**A**) Gram-positive bacteria	(**B**) Gram-negative bacteria

Figure SI2. Photomicrographs of liver sections stained immunohistochemically with anti-caspase 3 antibody in tested '**4h**' compound groups: Negative expression of anti-caspase 3 was detected in negative control G1 **(A)**, G4 **(B),** and G5 **(C).** Strong positive expression in all hepatocytes marked sections from G8 **(D)** and G11 **(G).** Notice the moderate reactivity in hepatic tissue from G9 **(E)** and G12 **(H),** as well as the low ones highlighting G10 **(F)** and G13 **(I)** sections. (Anti-Caspase 3, 100x Magnification, Scale bar = 200µm).

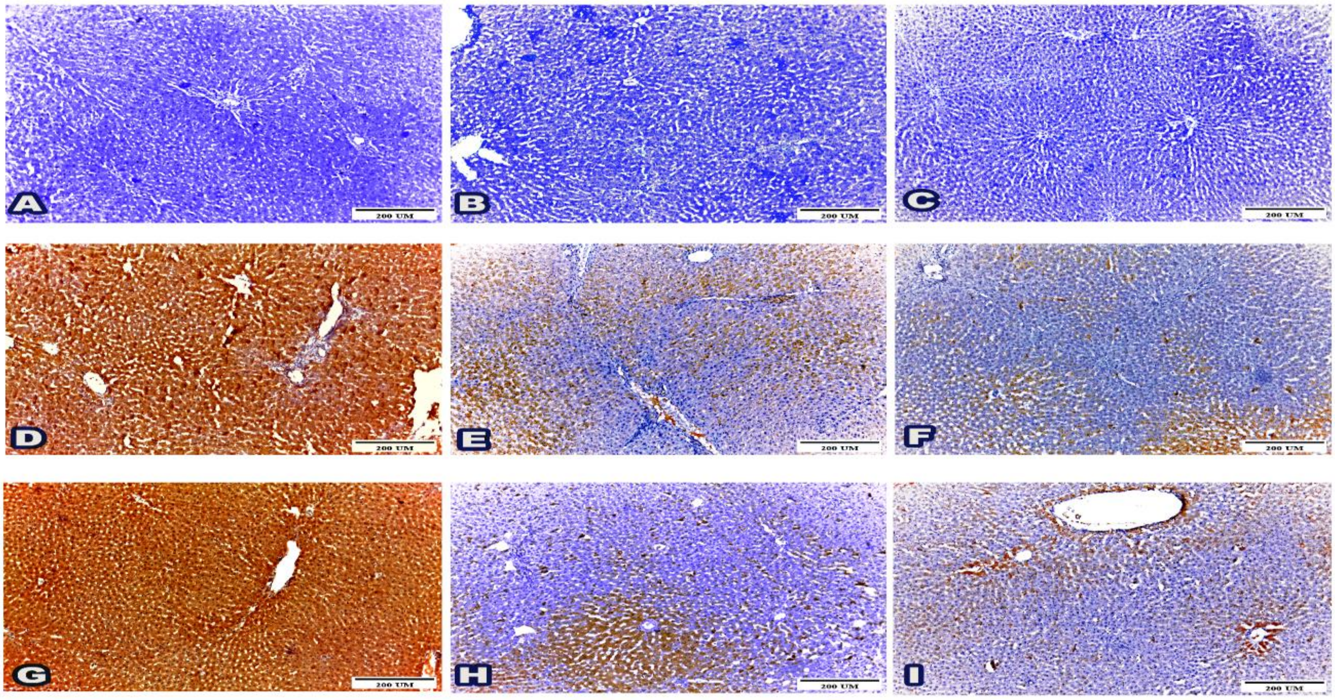

Figure SI3. Photomicrographs of liver sections marked immunohistochemically with anti-caspase 3 antibody in tried '**4i**' compound groups: Negative expression of anti-caspase 3 was distinguished in sections from negative control G1**(A)**, G6 **(B),** and G7 **(C).** Intense positive expression of all hepatocytes highlighted in G11 **(D).** Observe the moderate and low reactivity in hepatic tissue from G14 **(E)** and G15 **(F),** respectively. (Anti-Caspase 3, 100x Magnification, Scale bar = 200μm).

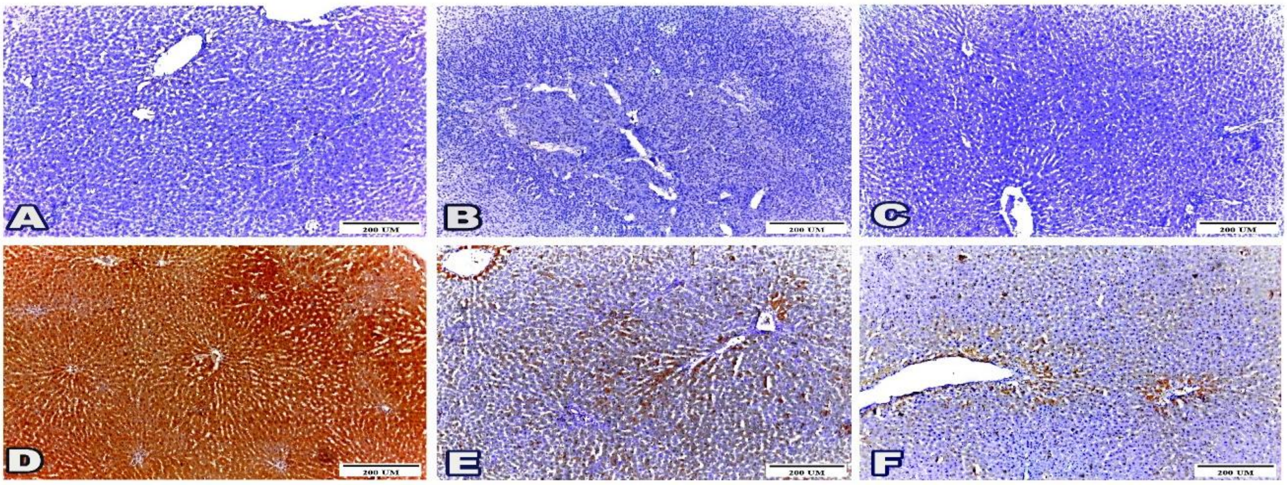

Figure SI4. Photomicrographs of kidney sections stained immunohistochemically with anti-caspase 3 antibody in examined '**4h**' compound groups: No expression of anti-caspase 3 was visualized in G1**(A)**, G4 **(B),** and G5 **(C).** Great positive reactivity populated in all renal tissue from G8 **(D)** and G11**(G).** Notice the moderate reactivity in renal tissue from G9 **(E)** and G12 **(H),** besides the low expression homing G10 **(F)** and G13 **(I)** sections. (Anti-Caspase 3, 100x Magnification, Scale bar = 200μm).

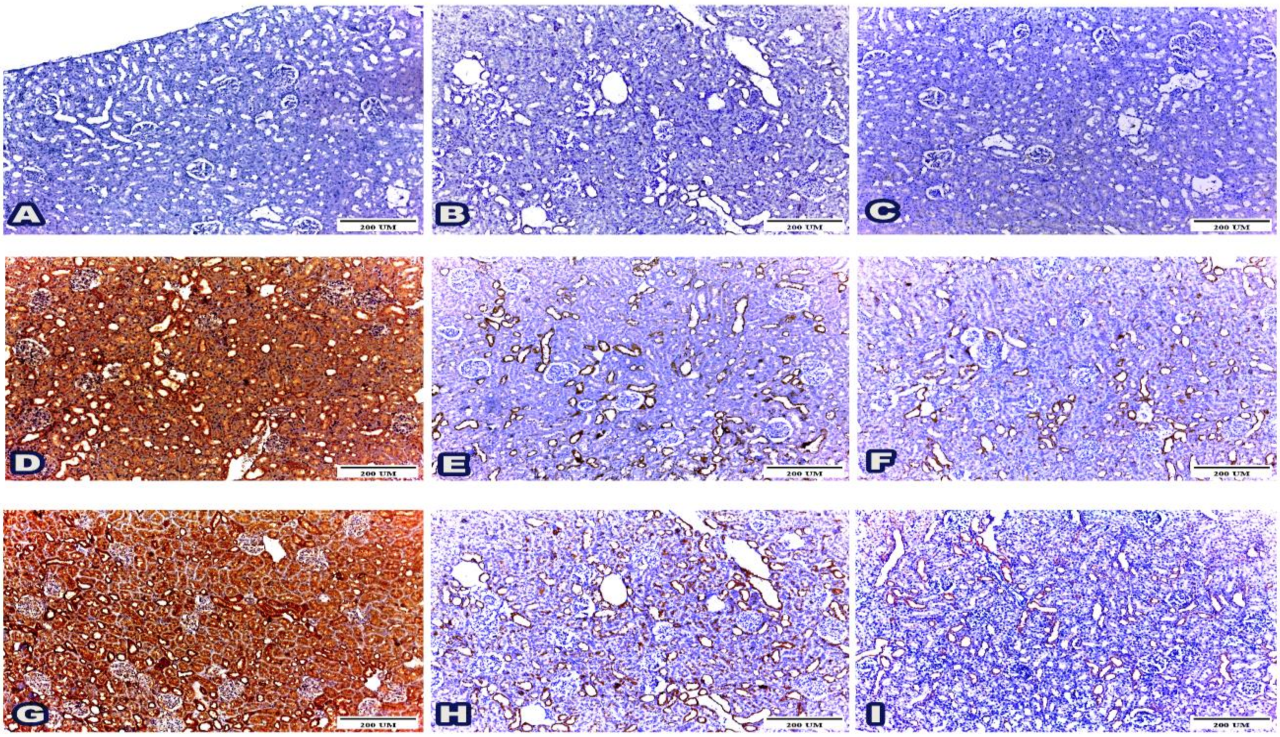

Figure SI5. Photomicrographs of kidney sections presented the immunohistochemical reactivity of anti-caspase 3 antibody in experimented '**4i**' compound groups: No expression of anti-caspase 3 was observed in G1**(A)**, G6 **(B),** and G7 **(C)** sections. Strong positive expression highlighted renal tissue in G11 **(D).** Notice the moderate and low reactivity in renal tissue from G14 **(E)** and G15 **(F),** respectively. (Anti-Caspase 3, 100x Magnification, Scale bar = 200μm).

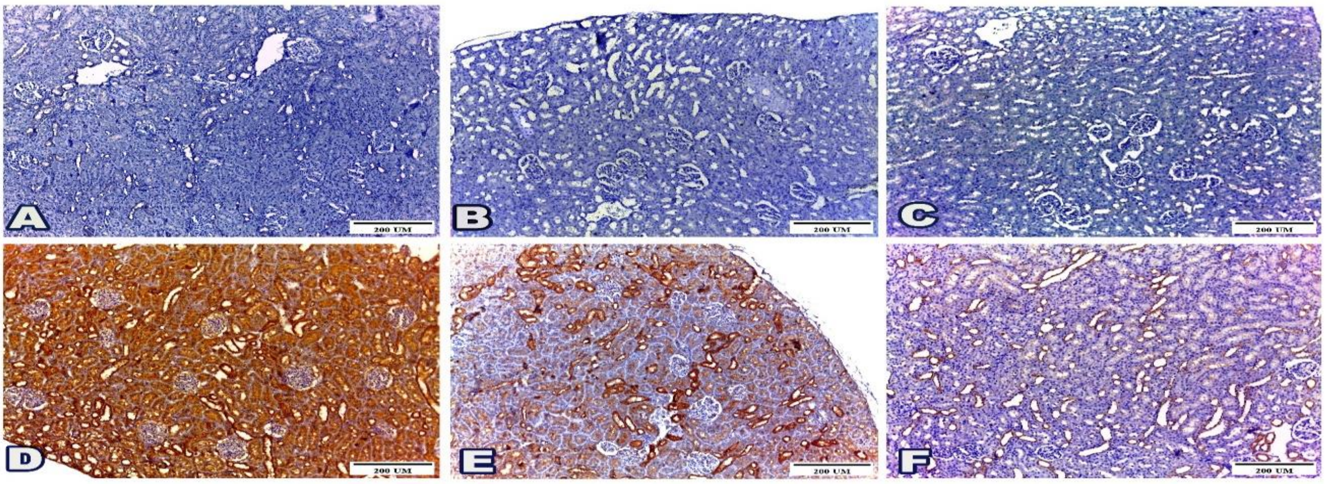

Figure SI6. Photomicrographs of liver sections marked immunohistochemically with anti-Nuclear Factor Kappa-B antibody in tested '**4h**' compound groups: Negative expression of anti-NF-kB was recorded in negative control G1 **(A)**, G4 **(B),** and G5 **(C).** Intense positive expression populated in all hepatocytes from G8 **(D)** and G11 **(G).** Observe the moderate reactivity in hepatic tissue from G9 **(E)** and G12 **(H),** in addition to the little ones prominent in G10 **(F)** and G13 **(I)** sections. (Anti-NF-kB, 100x Magnification, Scale bar = 200μm).

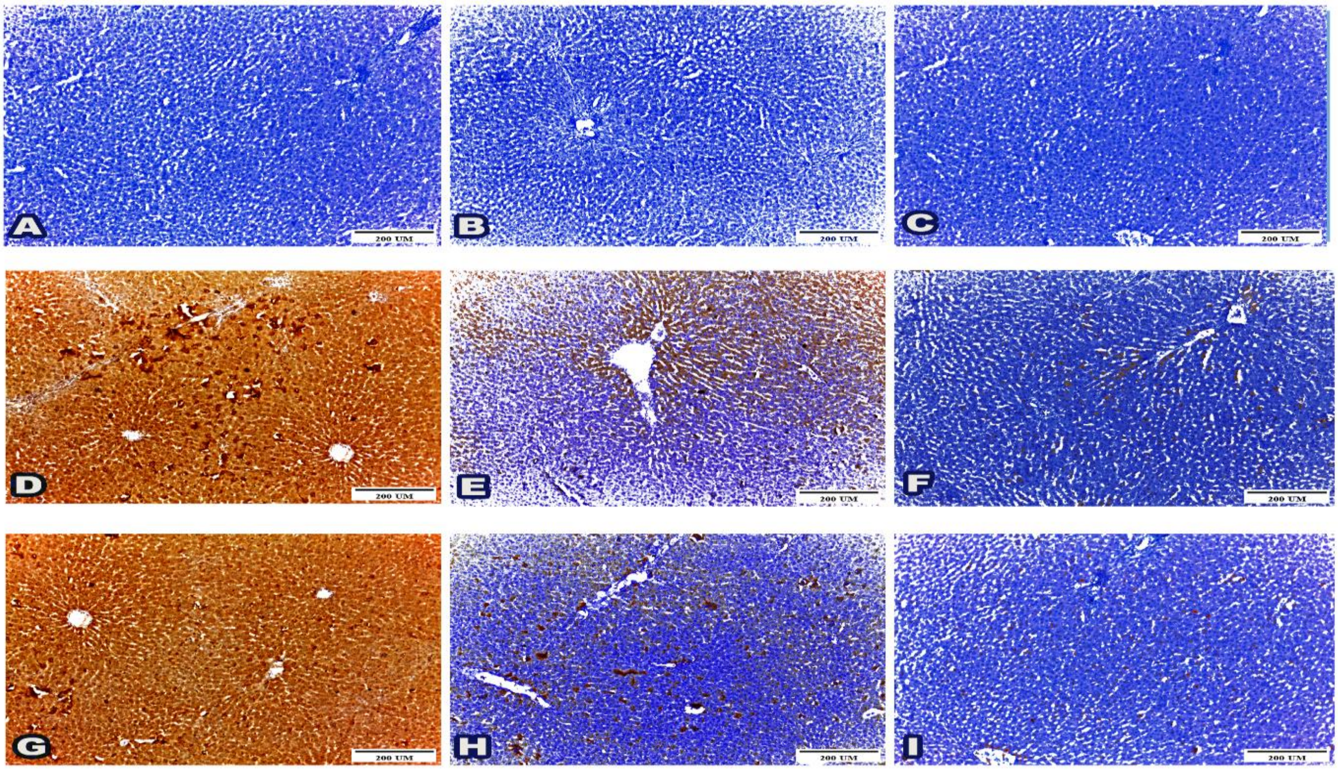

Figure SI7. Photomicrographs of liver sections indicated immunohistochemically with anti-NF-kB antibody in tested '**4i**' compound groups: Negative expression of anti- NF-kB was eminent in sections from negative control G1 **(A)**, G6 **(B),** and G7 **(C).** Concentrated positive expression of all hepatocytes emphasized in G11 **(D).** Notice the moderate and few reactivities in hepatic tissue from G14 **(E)** and G15 **(F),** respectively. (Anti- NF-kB, 100x Magnification, Scale bar = 200μm).

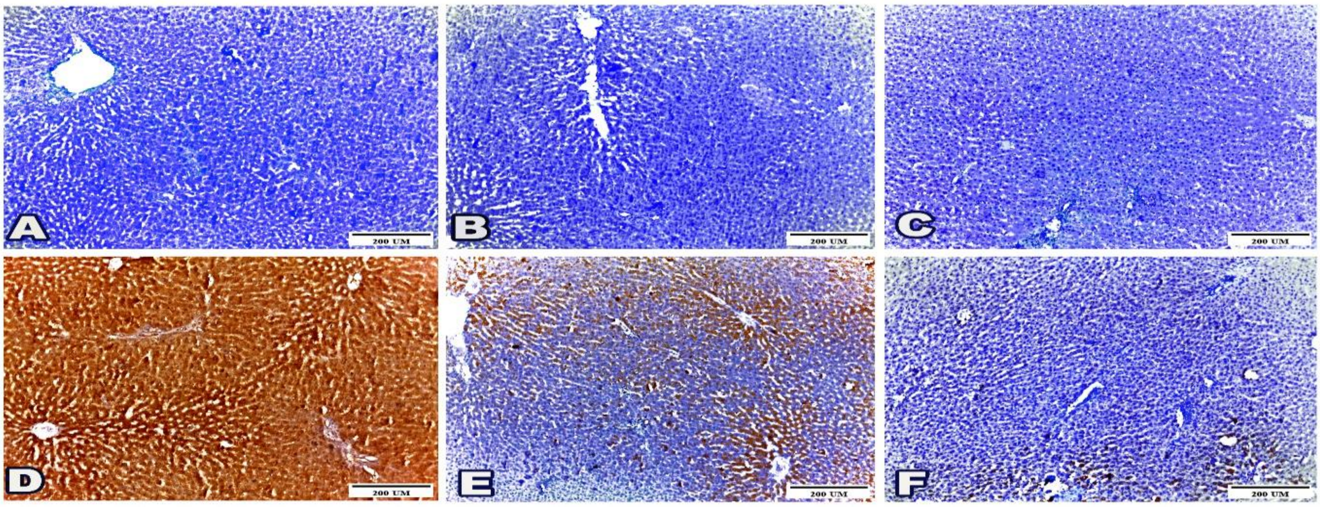

Figure SI8. Photomicrographs of kidney sections stained immunohistochemically with anti-NF-kB antibody in inspected '**4h**' compound groups: No expression of anti- NF-kB was pictured in G1 **(A)**, G4 **(B),** and G5 **(C).** Excessive positive reactivity populated in all renal tissue from G8 **(D)** and G11 **(G).** Notice the moderate responsiveness in renal tissue from G9 **(E)** and G12 **(H),** as well the little expression reported from G10 **(F)** and G13 **(I)** sections. (Anti-NF-kB, 100x Magnification, Scale bar = 200μm).

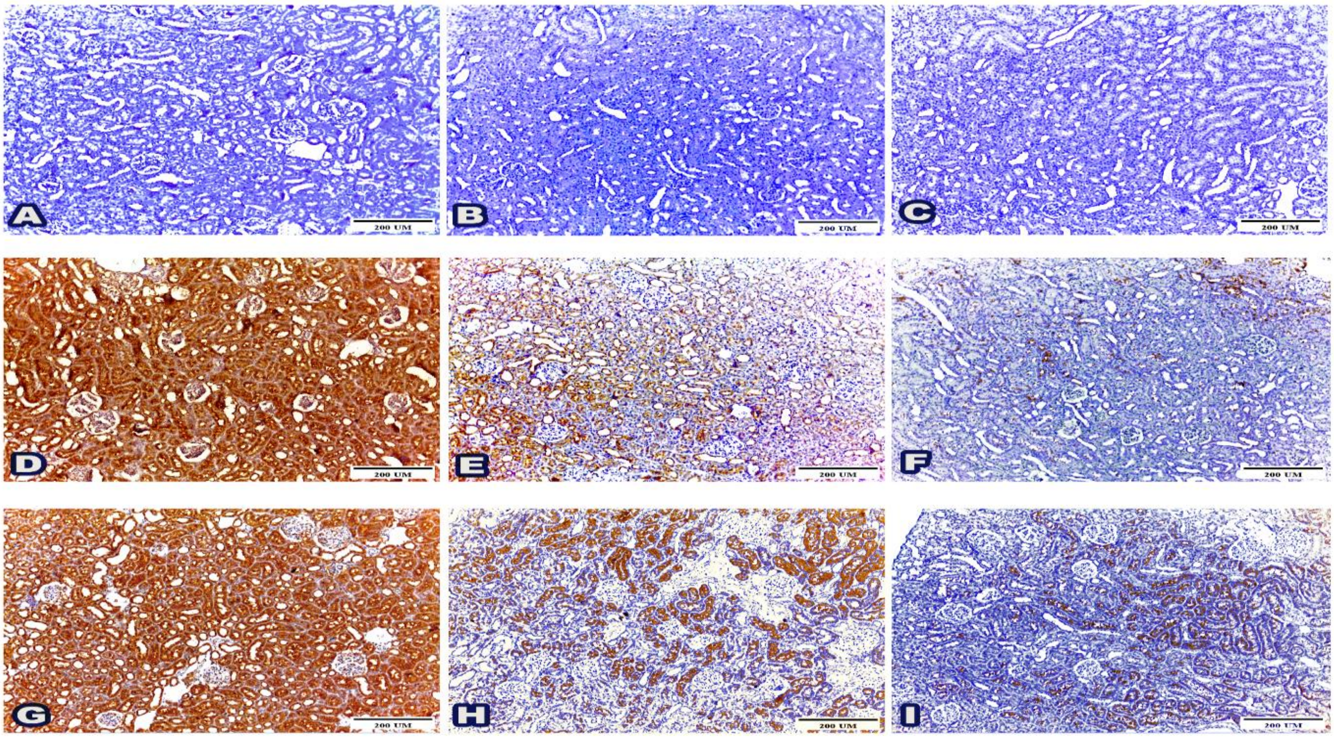

Figure SI9. Photomicrographs of kidney sections represented the immunohistochemical reactivity of anti-NF-kB antibody in tried '**4i**' compound groups: Negative expression of anti-NF-kB was detected in G1 **(A)**, G6 **(B),** and G7 **(C)** sections. Intense positive reactivity was emphasized in renal tissue in G11 **(D).** Notice the moderate and low expression in renal tissue from G14 **(E)** and G15 **(F),** respectively. (Anti-NF-kB, 100x Magnification, Scale bar = 200μm).

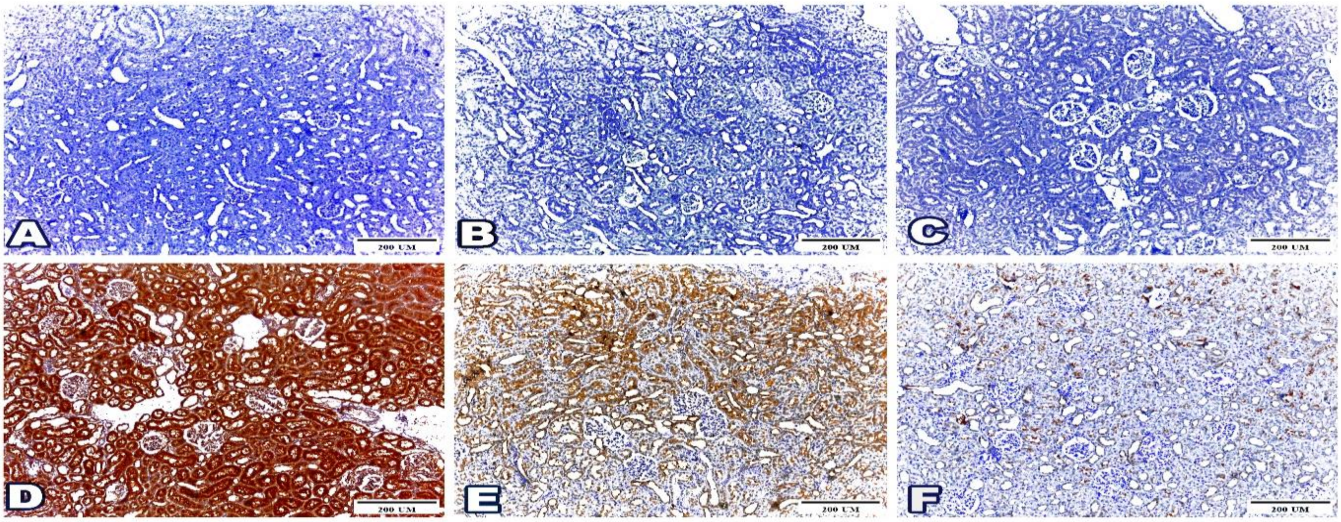

Table SI1. The diverse derivatives of the investigated synthesized compounds, Their MIC values (µM), and their predicted WLogP.

Compound ID	-R	EF	A A	AS	AM	P A	SA	ST	EC	CA	Average MIC
**4a**	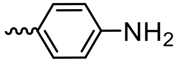	52.15	104.60	52.15	104.60	52.15	26.11	52.15	52.15	26.11	58.02
**4b**	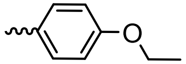	380.90	190.50	47.54	380.90	23.80	95.39	23.80	23.80	95.39	140.22
**4c**	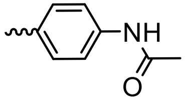	45.73	91.75	45.73	183.20	22.89	45.73	183.20	22.89	91.75	81.43
**4d**	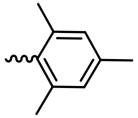	95.97	191.60	191.60	383.20	95.97	47.83	95.97	47.83	191.60	149.06
**4e**	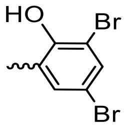	137.10	34.22	17.13	137.10	68.65	34.22	68.65	17.13	137.10	72.37
**4f**	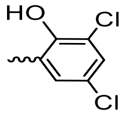	42.39	42.39	21.22	85.05	85.05	169.80	85.05	42.39	85.05	73.15
**4g**	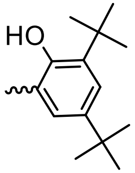	18.95	75.93	37.84	151.60	75.93	151.60	37.84	75.93	37.84	73.72
**4h**	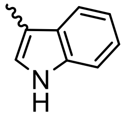	96.87	48.28	24.17	193.40	193.40	5.88	12.07	48.28	386.80	112.13
**4i**	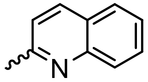	93.40	11.64	46.55	373.00	186.50	46.55	93.40	23.30	23.30	99.74
**4j**	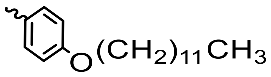	16.68	16.68	33.31	133.50	33.31	66.84	33.31	66.84	266.90	74.15

EF; E. faecalis, A A; Acientobacter ATCC 19606, AS; Acientobacter sensitive, AM; Aciento MDR, PA; P.auriginosa, SA; S. aureusST; S. Typhi EC; E. coli, CA; C. albicans.

Table SI2. The average MIC values (µM), predicted logP, and docking scores of the investigated compounds for multiple linear regression construction using excel.

Compound ID	Average MIC	Predicted Log P	Docking scores
**4a**	58.02	2.06	-6.67
**4b**	140.22	2.87	-7.21
**4c**	81.43	2.24	-7.29
**4d**	149.06	3.39	-7.21
**4e**	72.37	3.70	-7.25
**4f**	73.15	3.48	-7.02
**4g**	73.72	4.77	-8.48
**4h**	112.13	2.95	-6.65
**4i**	99.74	3.02	-7.07
**4j**	74.15	6.77	-9.47

SI1. IR, ^{1}H NMR, ^{13}C NMR and Mass spectroscopy data for the synthesized compounds **4a-4j**.

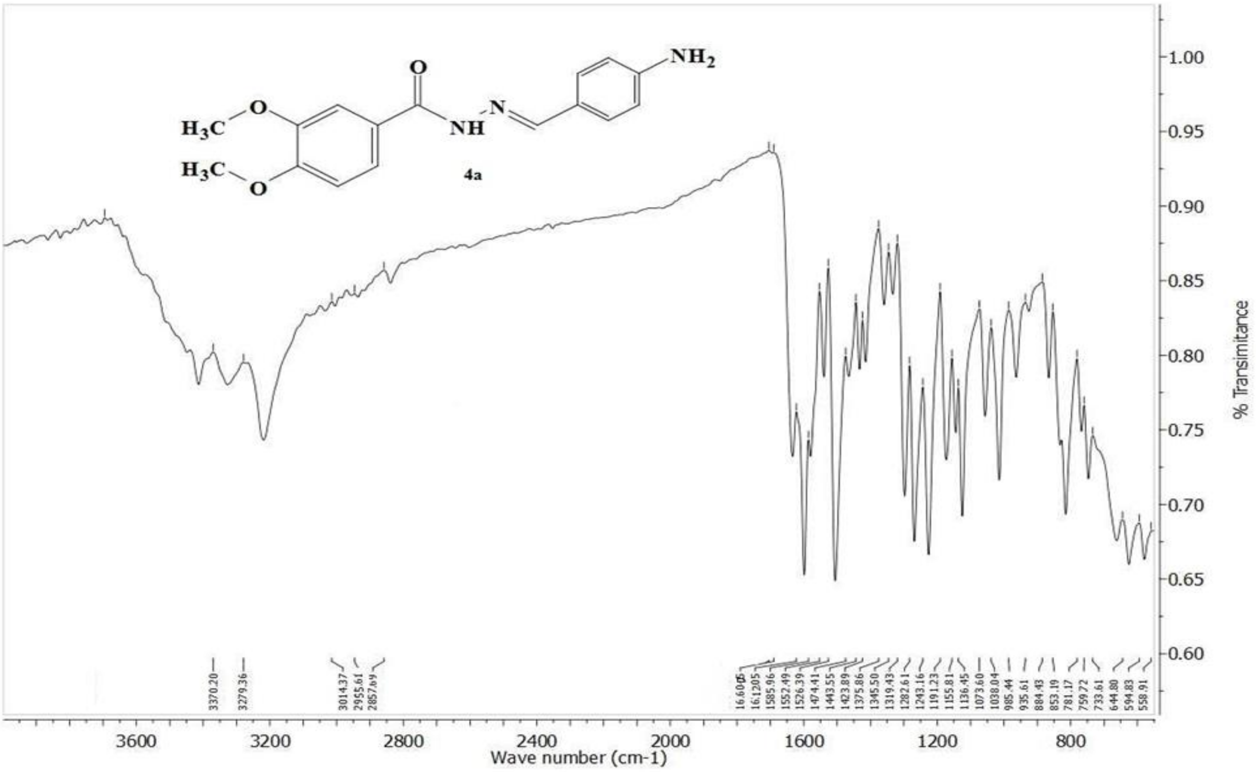

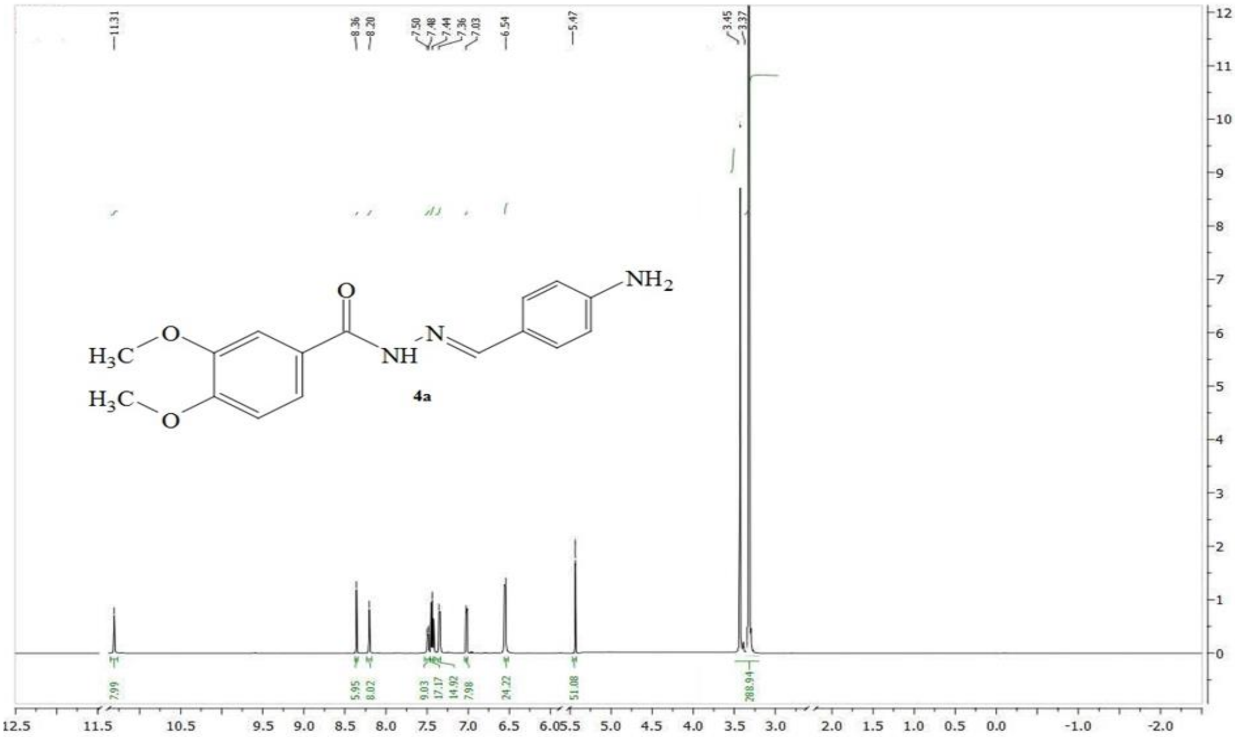

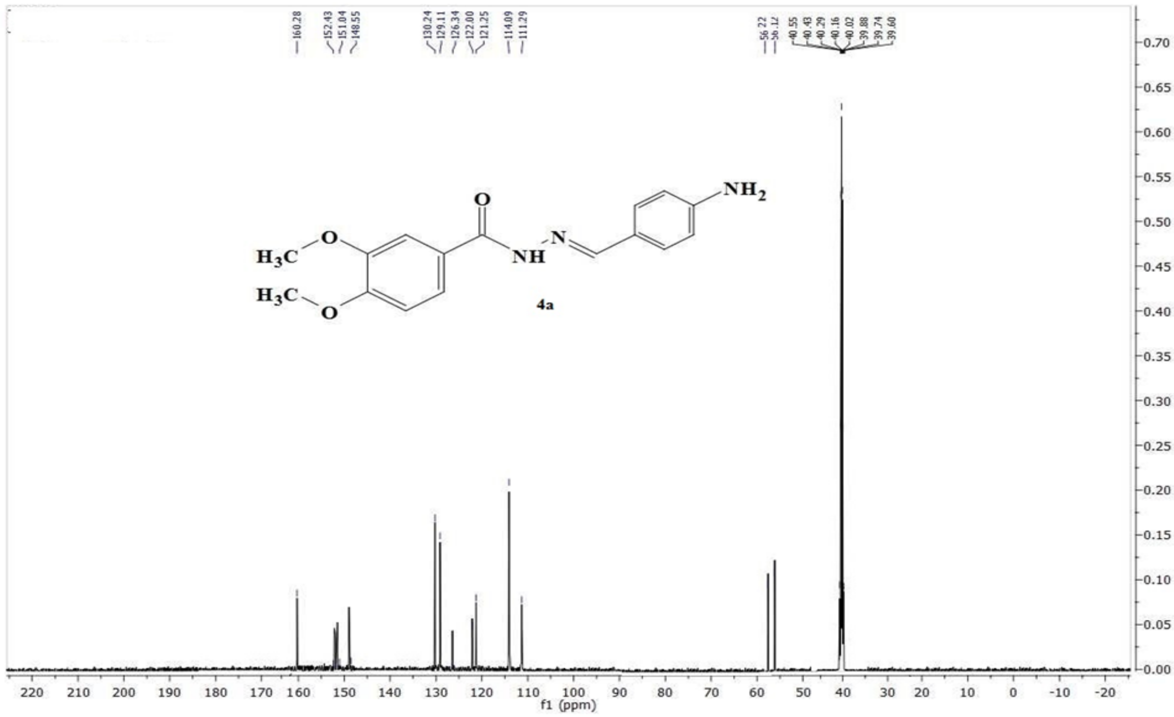

4a
f1 (ppm)

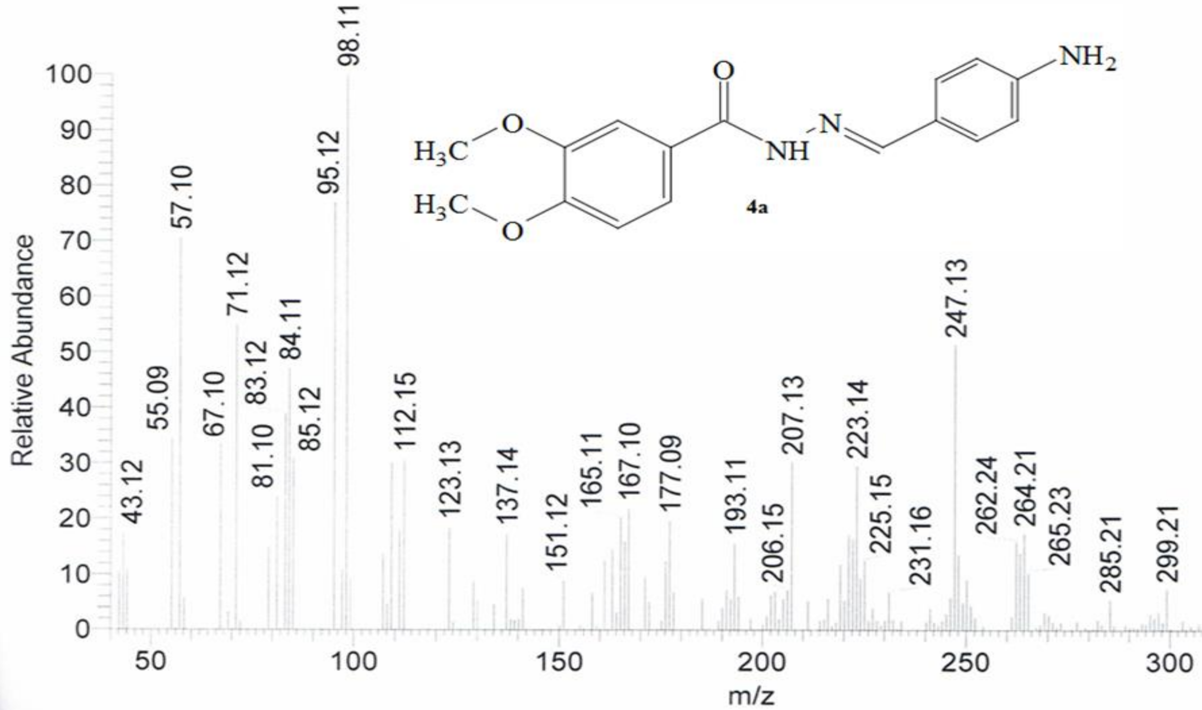

Relative Abundance
m/z
4a

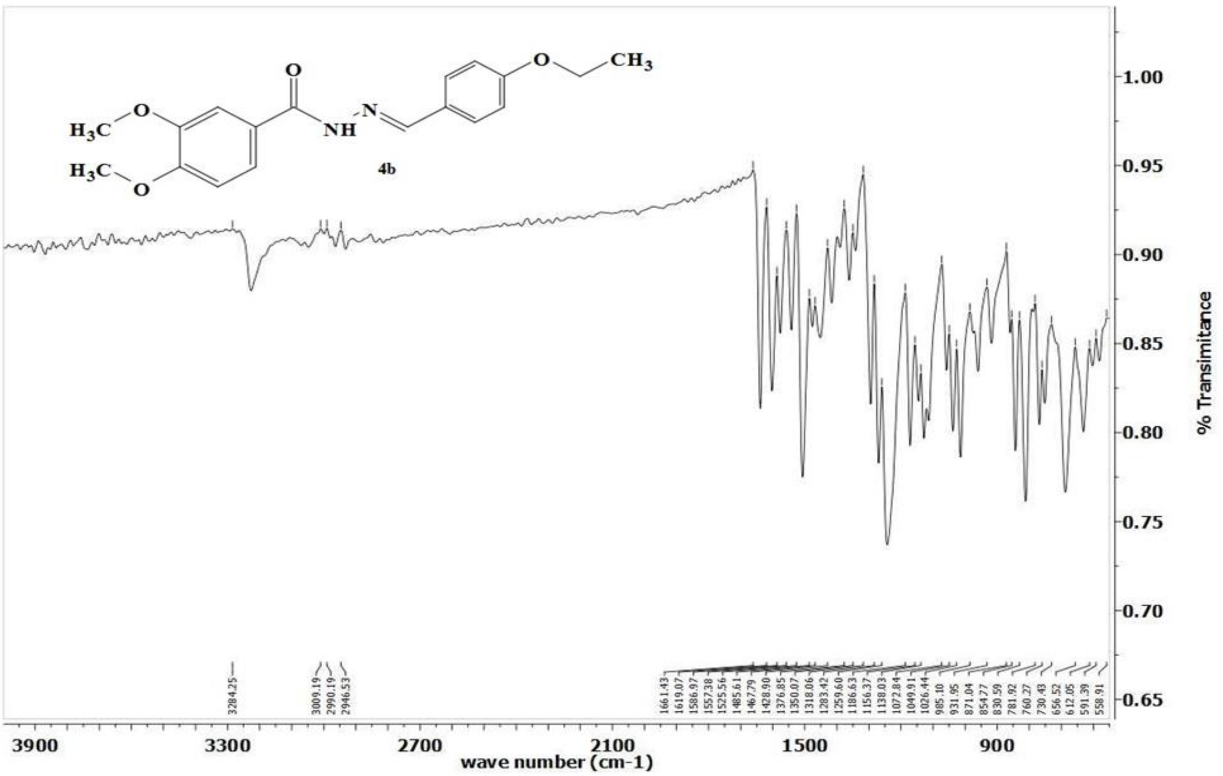

O
H_3C O
H_3C O
NH N
O CH3
4b
3284.25
3009.19 2990.19 2946.53
1661.43 1619.07 1586.97 1557.38 1525.56 1485.61 1467.79 1428.90 1376.85 1350.07 1318.06 1283.42 1259.60 1186.63 1156.37 1138.03 1072.84 1049.91 1026.44 985.10 931.95 871.04 854.77 830.59 781.92 760.27 730.43 656.52 612.05 591.39 558.91
1.00 0.95 0.90 0.85 0.80 0.75 0.70 0.65
% Transimitance
3900 3300 2700 2100 1500 900
wave number (cm-1)

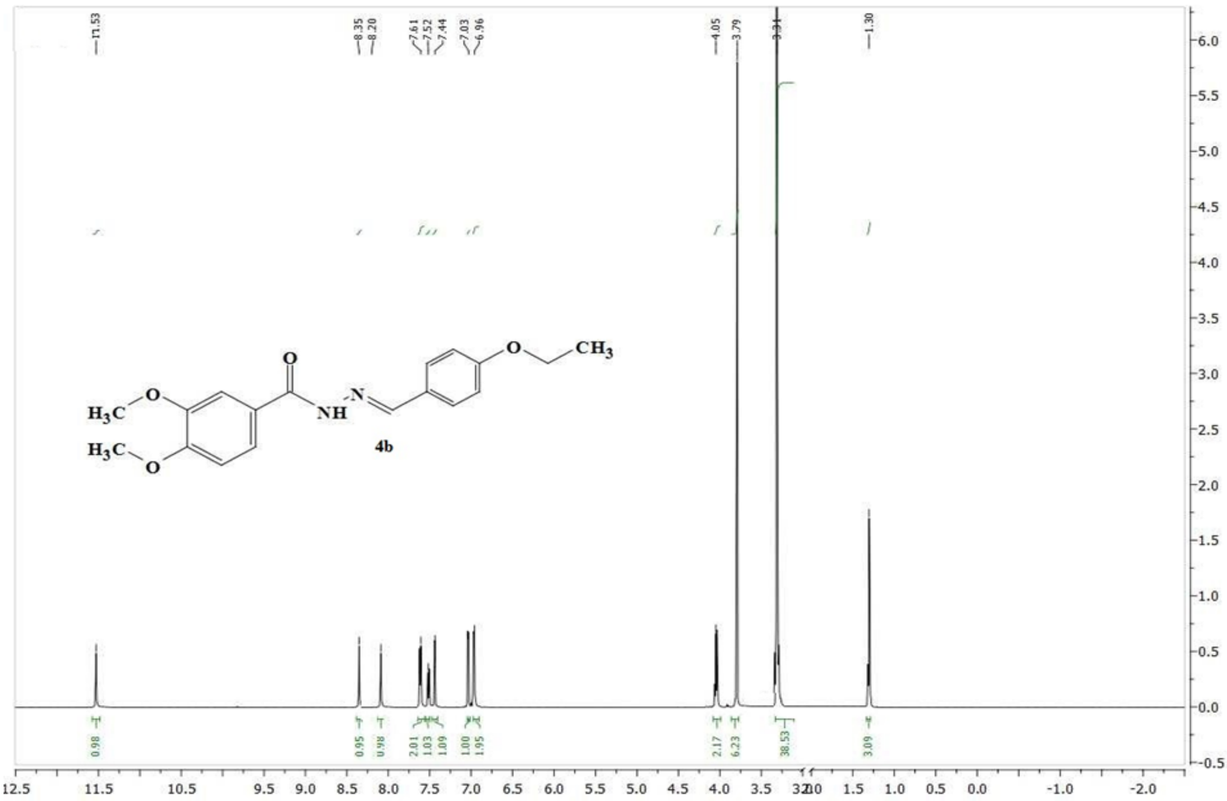

11.53
8.35 8.20
7.61 7.52 7.44 7.03 6.96
4.05 3.79 3.31 1.30
O
H_3C O
H_3C O
NH N
O CH3
4b
0.98 0.95 0.98 2.01 1.03 1.09 1.00 1.95 2.17 6.23 38.53 3.09
6.0 5.5 5.0 4.5 4.0 3.5 3.0 2.5 2.0 1.5 1.0 0.5 0.0 -0.5
12.5 11.5 10.5 9.5 9.0 8.5 8.0 7.5 7.0 6.5 6.0 5.5 5.0 4.5 4.0 3.5 3.2 2.0 1.5 1.0 0.5 0.0 -1.0 -2.0

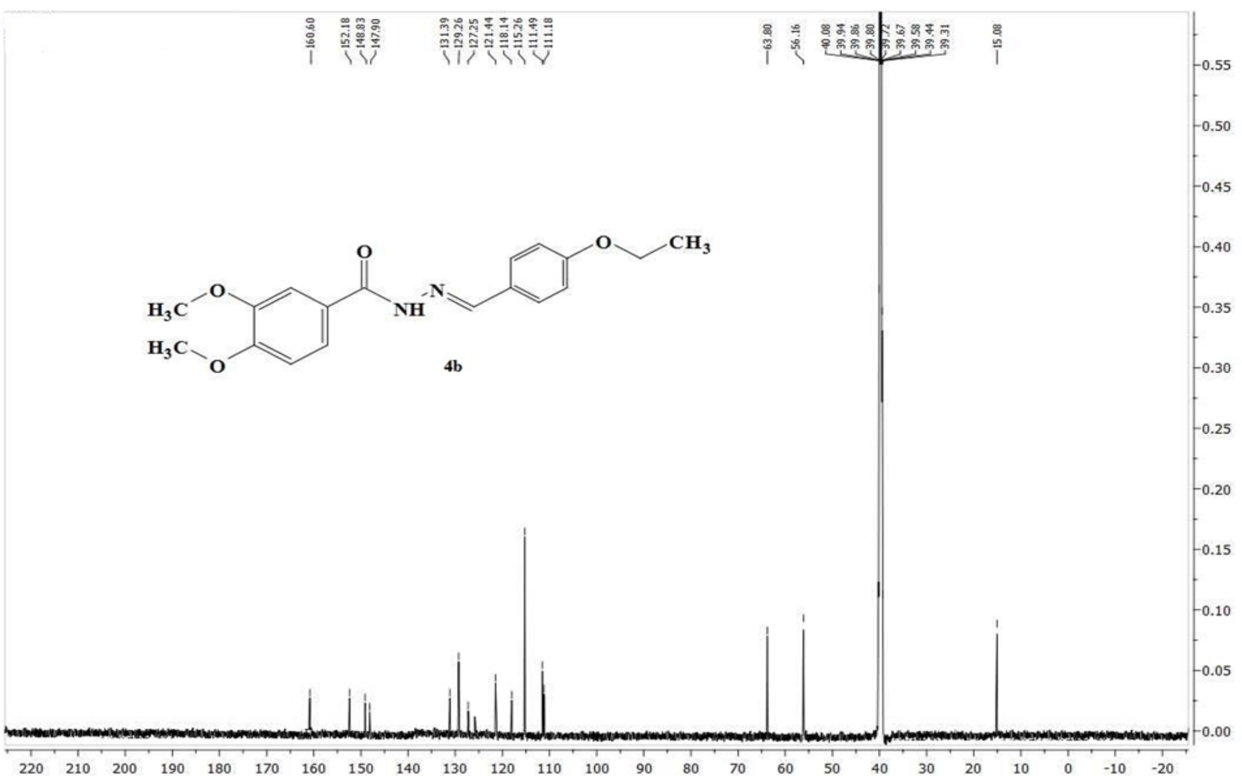

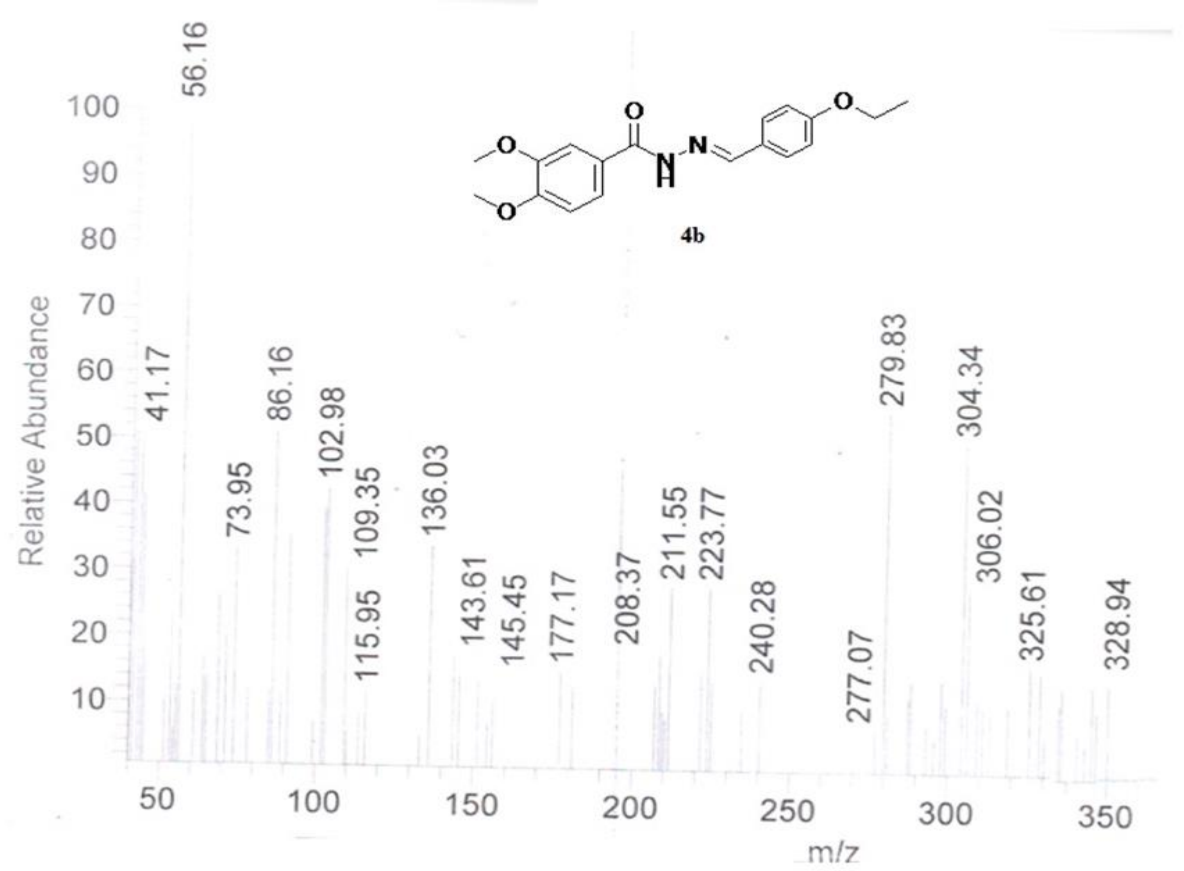

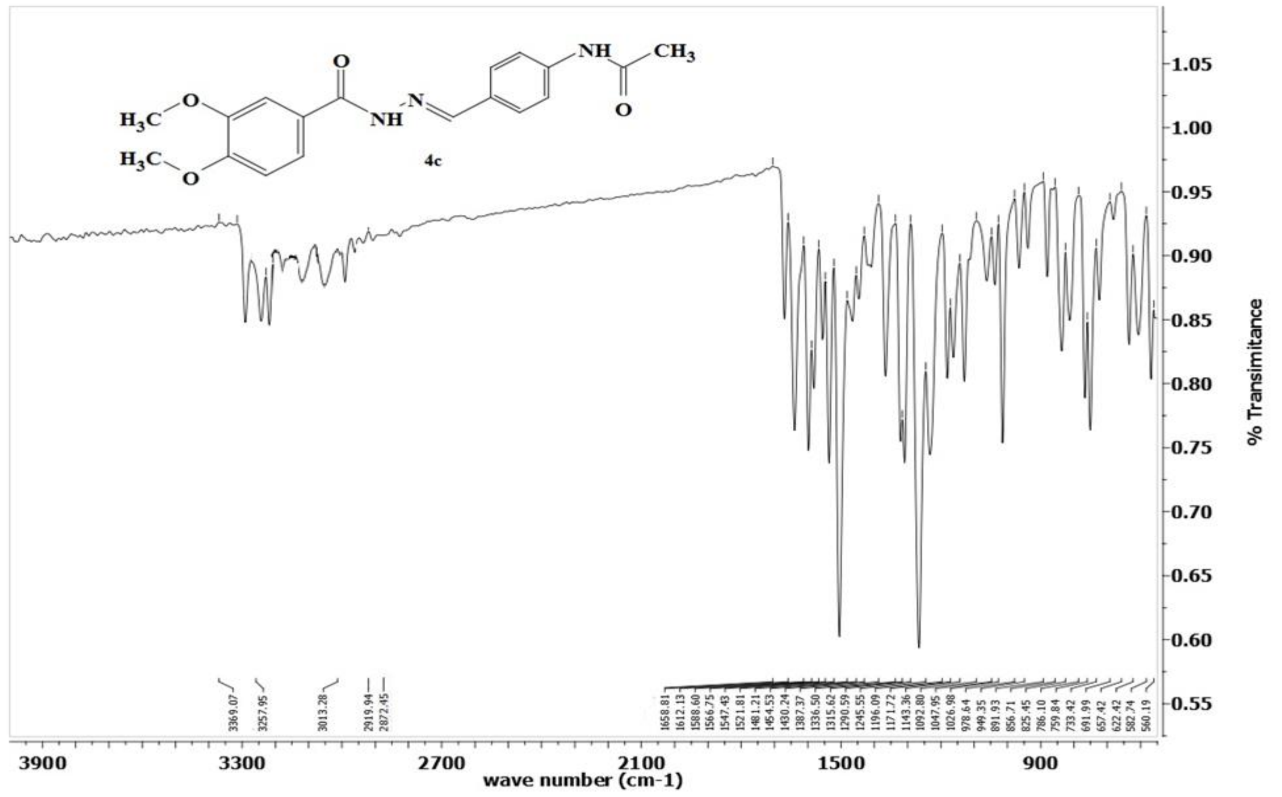

4c
% Transimitance
wave number (cm-1)
3369.07
3257.95
3013.28
2919.94
2872.45

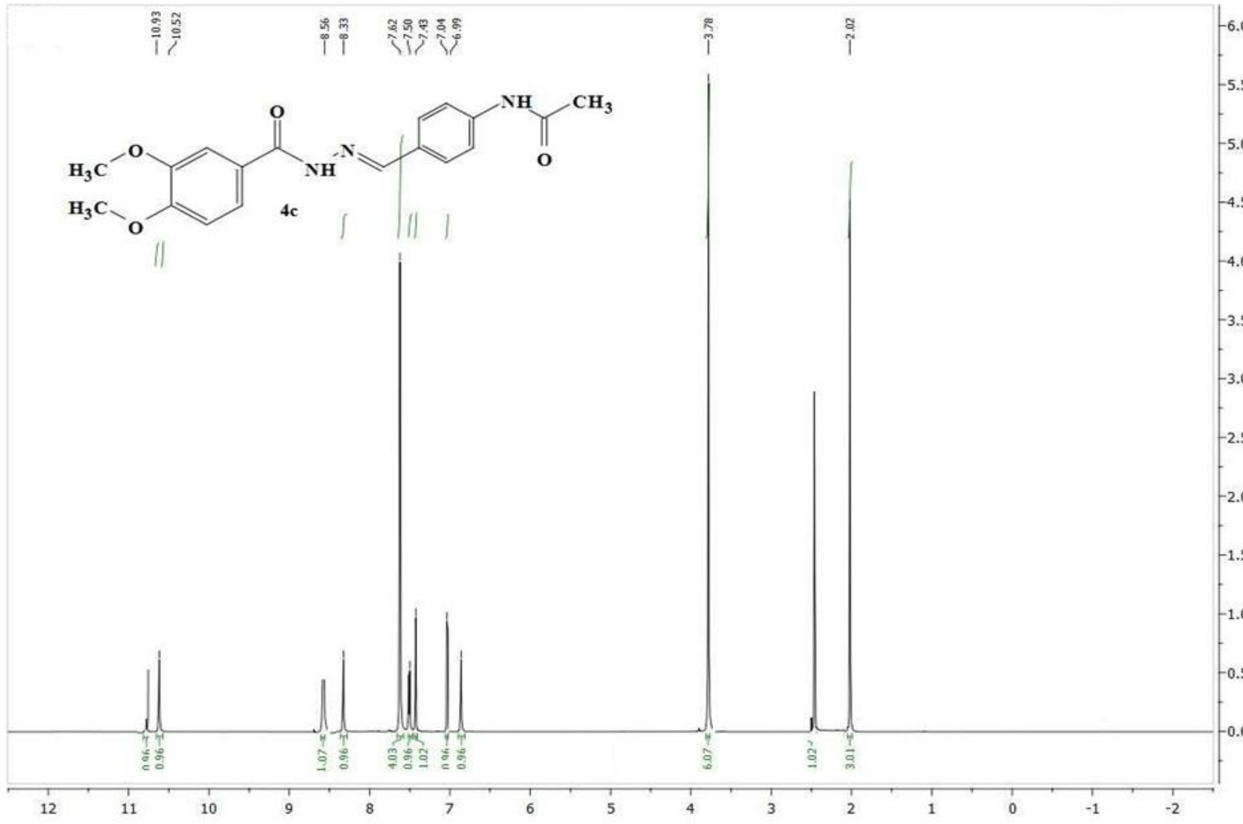

4c
10.93
10.52
8.56
8.33
7.62
7.50
7.43
7.04
6.99
3.78
2.02

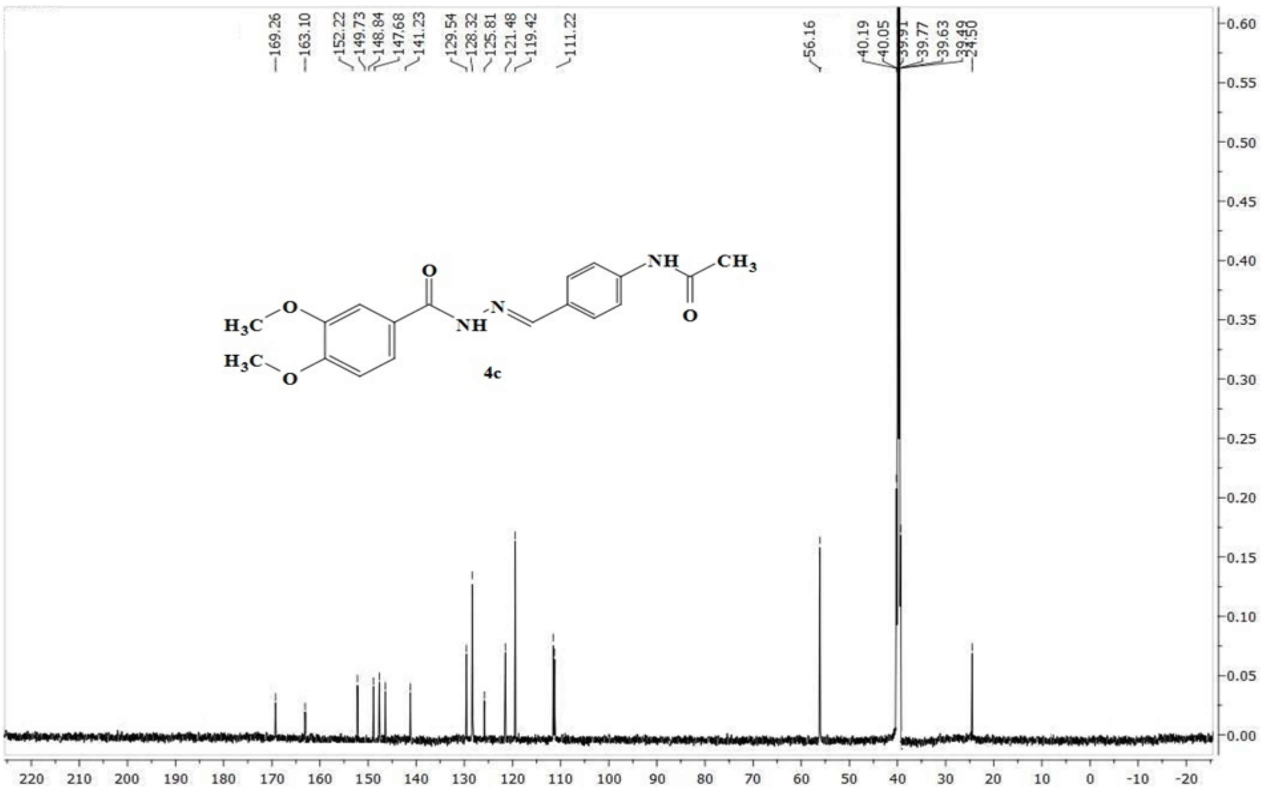

169.26
163.10
152.22
149.73
148.84
147.68
141.23
129.54
128.32
125.81
121.48
119.42
111.22
56.16
40.19
40.05
39.91
39.77
39.63
39.49
24.50
H_3C
H_3C
O
NH
N
NH
CH_3
4c

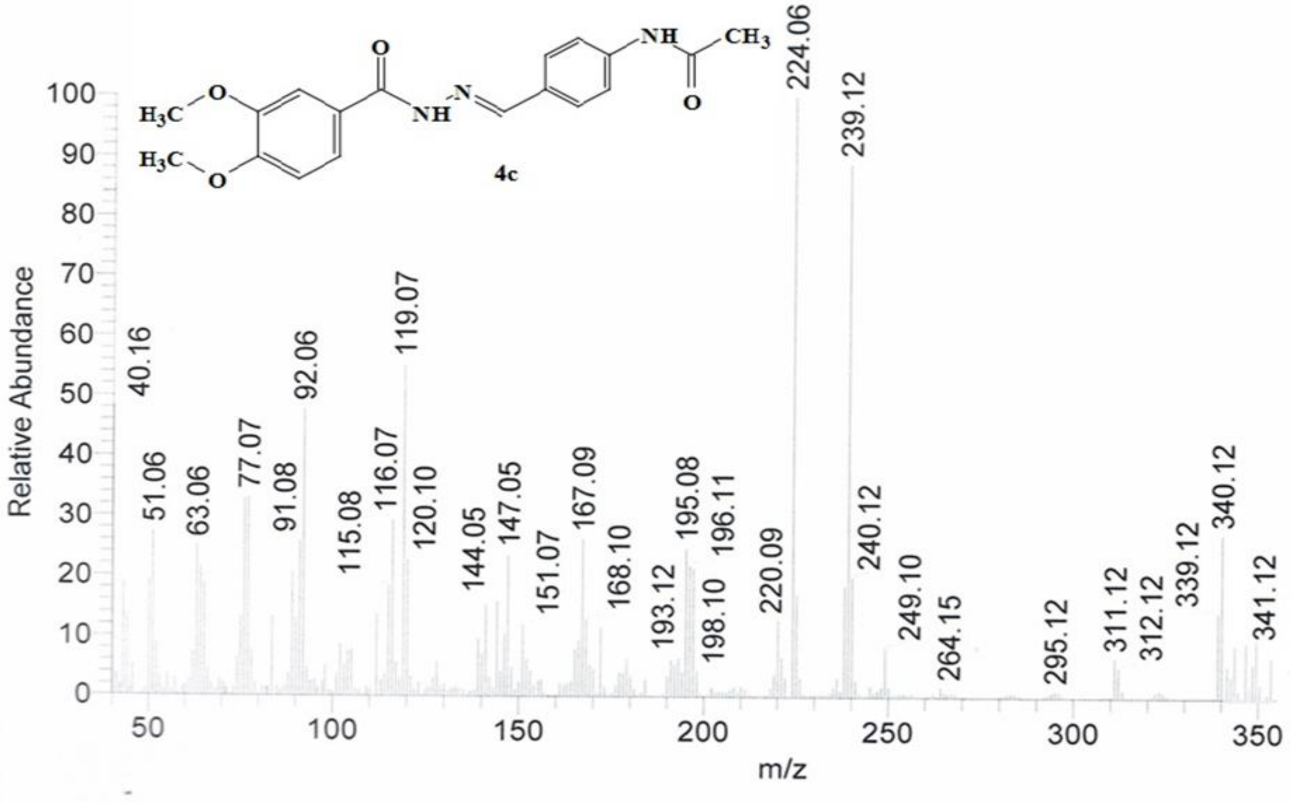

Relative Abundance
m/z
H_3C
H_3C
O
NH
N
NH
CH_3
4c
40.16
51.06
63.06
77.07
91.08
92.06
115.08
116.07
119.07
120.10
144.05
147.05
151.07
167.09
168.10
193.12
195.08
196.11
198.10
220.09
224.06
239.12
240.12
249.10
264.15
295.12
311.12
312.12
339.12
340.12
341.12

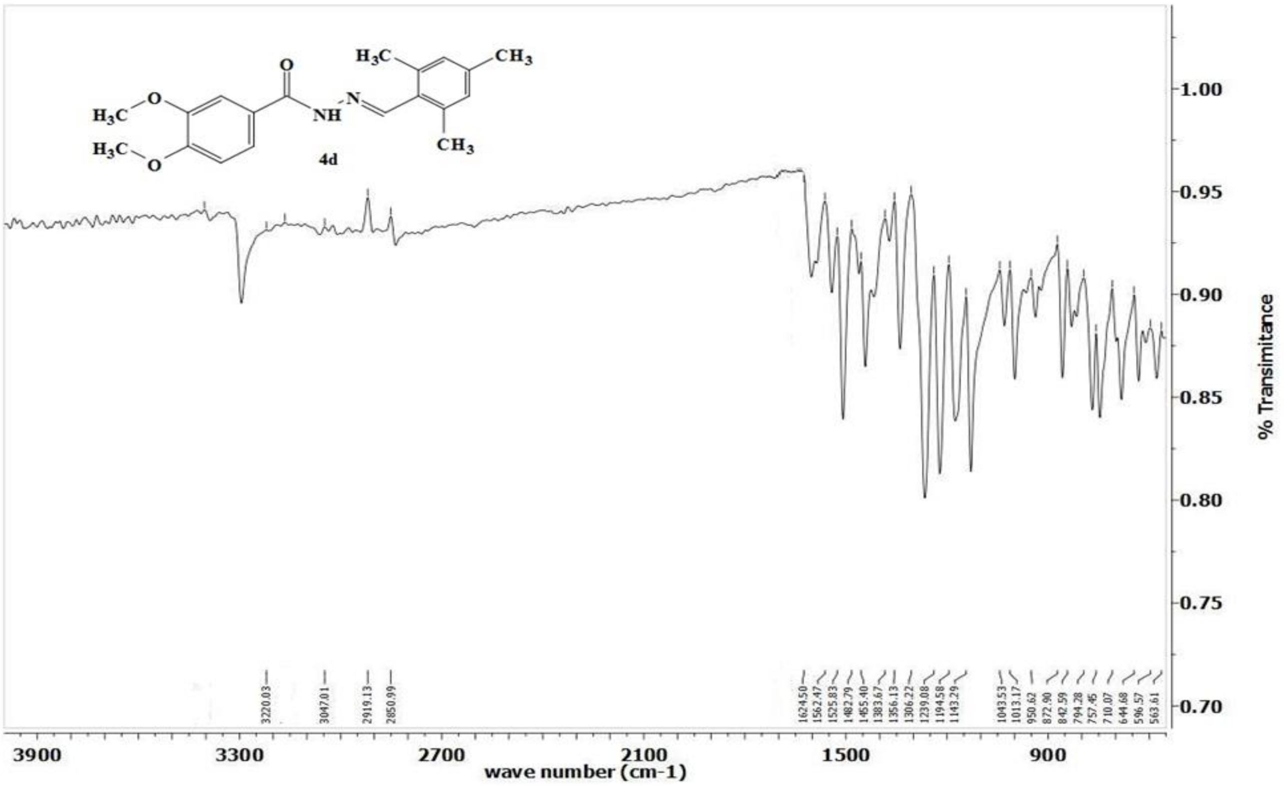

H3C
O
O
NH
N
H3C
CH3
CH3
H3C
4d
3220.03
3047.01
2919.13
2850.99
1624.50
1562.47
1525.83
1482.79
1455.40
1383.67
1356.13
1306.22
1239.08
1194.58
1143.29
1043.53
1013.17
950.62
872.90
842.59
794.28
757.45
710.07
644.68
596.57
563.61
1.00
0.95
0.90
0.85
0.80
0.75
0.70
% Transimitance
3900
3300
2700
2100
1500
900
wave number (cm-1)

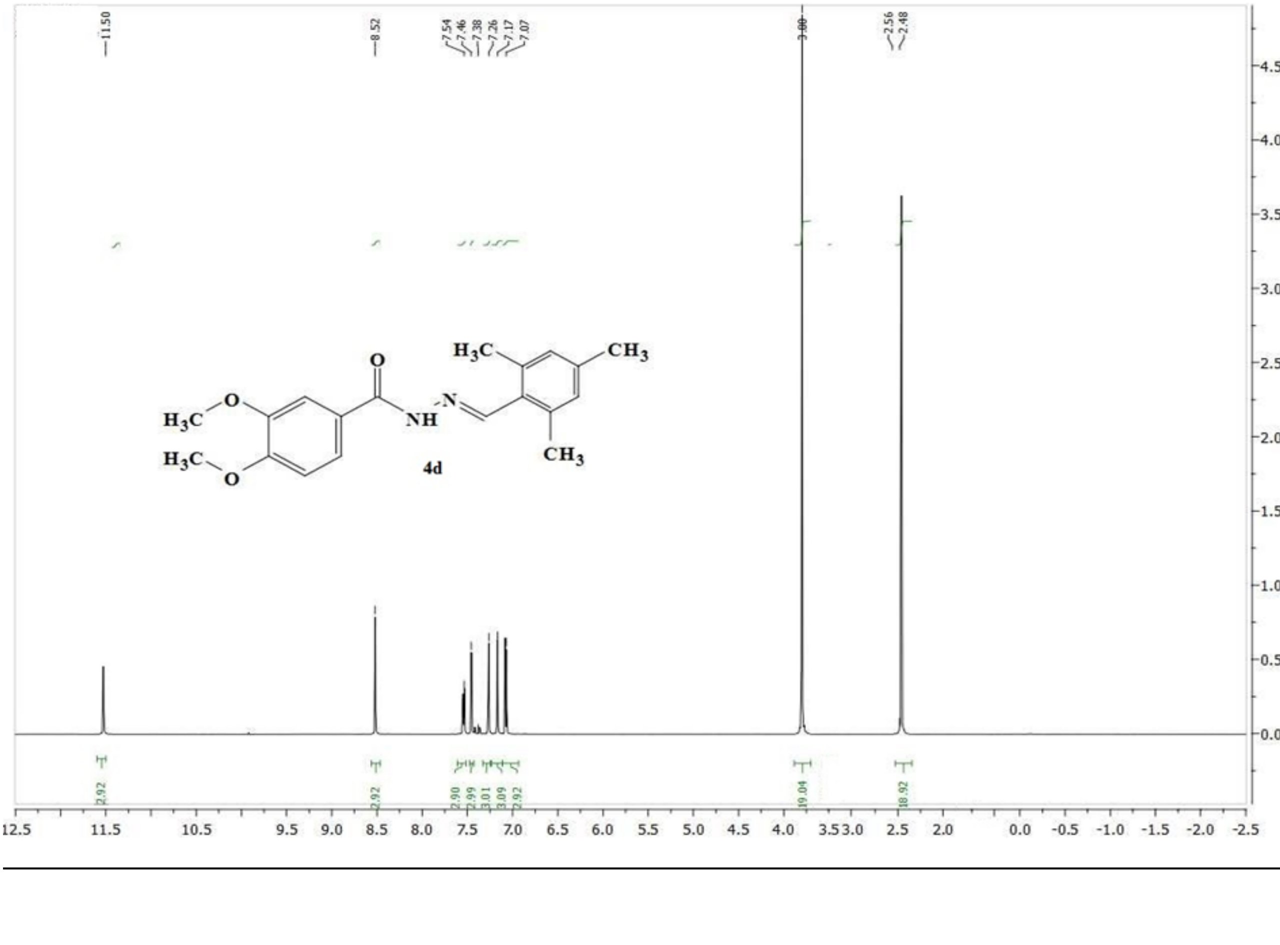

11.50
8.52
7.54
7.46
7.38
7.26
7.17
7.07
3.80
2.56
2.48
H3C
O
O
NH
N
H3C
CH3
CH3
H3C
4d
2.92
2.92
2.90
2.99
3.01
3.09
2.92
19.04
18.92
12.5
11.5
10.5
9.5
9.0
8.5
8.0
7.5
7.0
6.5
6.0
5.5
5.0
4.5
4.0
3.5
3.0
2.5
2.0
0.0
-0.5
-1.0
-1.5
-2.0
-2.5

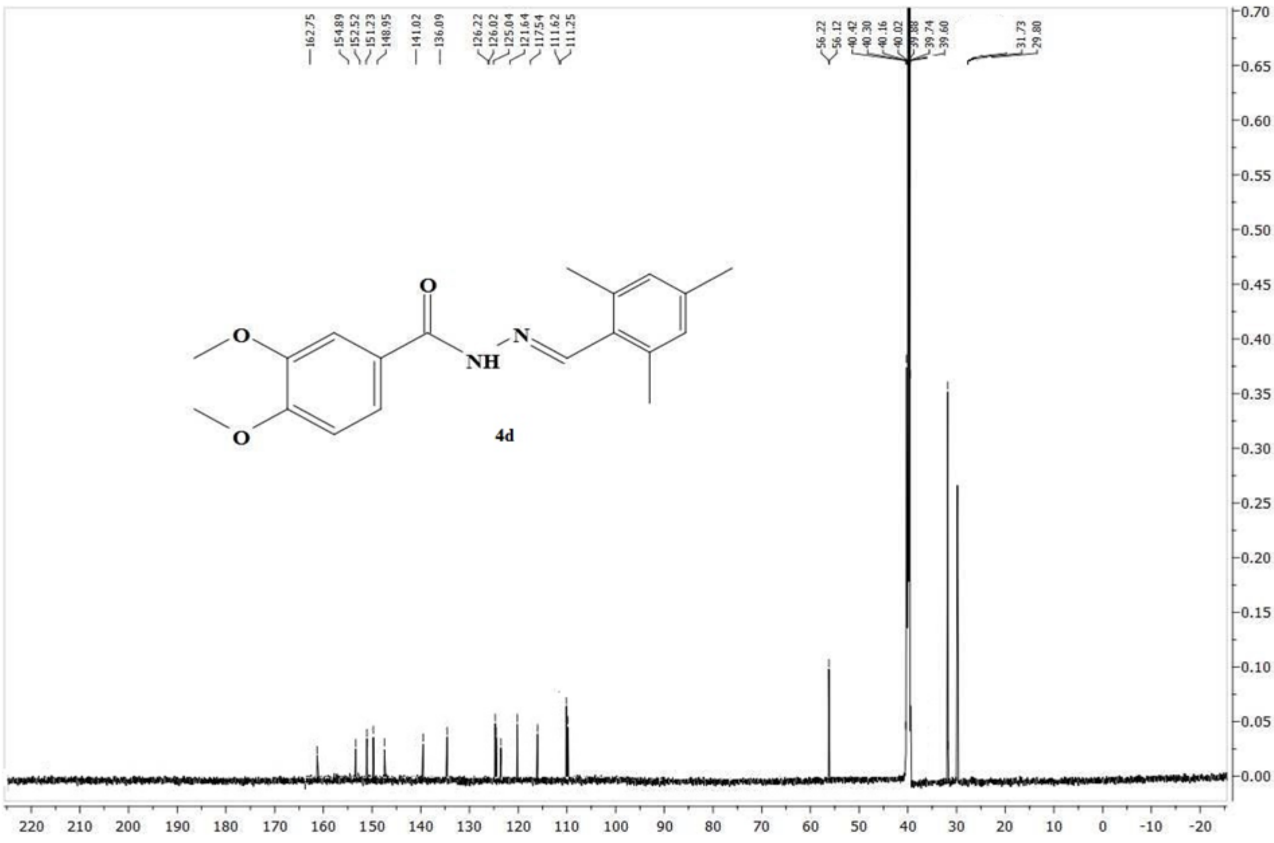
162.75
154.89
152.52
151.23
148.95
141.02
136.09
126.22
126.02
125.04
121.64
117.54
111.62
111.25
56.22
56.12
40.42
40.30
40.16
40.02
39.88
39.74
39.60
31.73
29.80
O
O
O
NH
N
4d
220 210 200 190 180 170 160 150 140 130 120 110 100 90 80 70 60 50 40 30 20 10 0 -10 -20
0.70 0.65 0.60 0.55 0.50 0.45 0.40 0.35 0.30 0.25 0.20 0.15 0.10 0.05 0.00

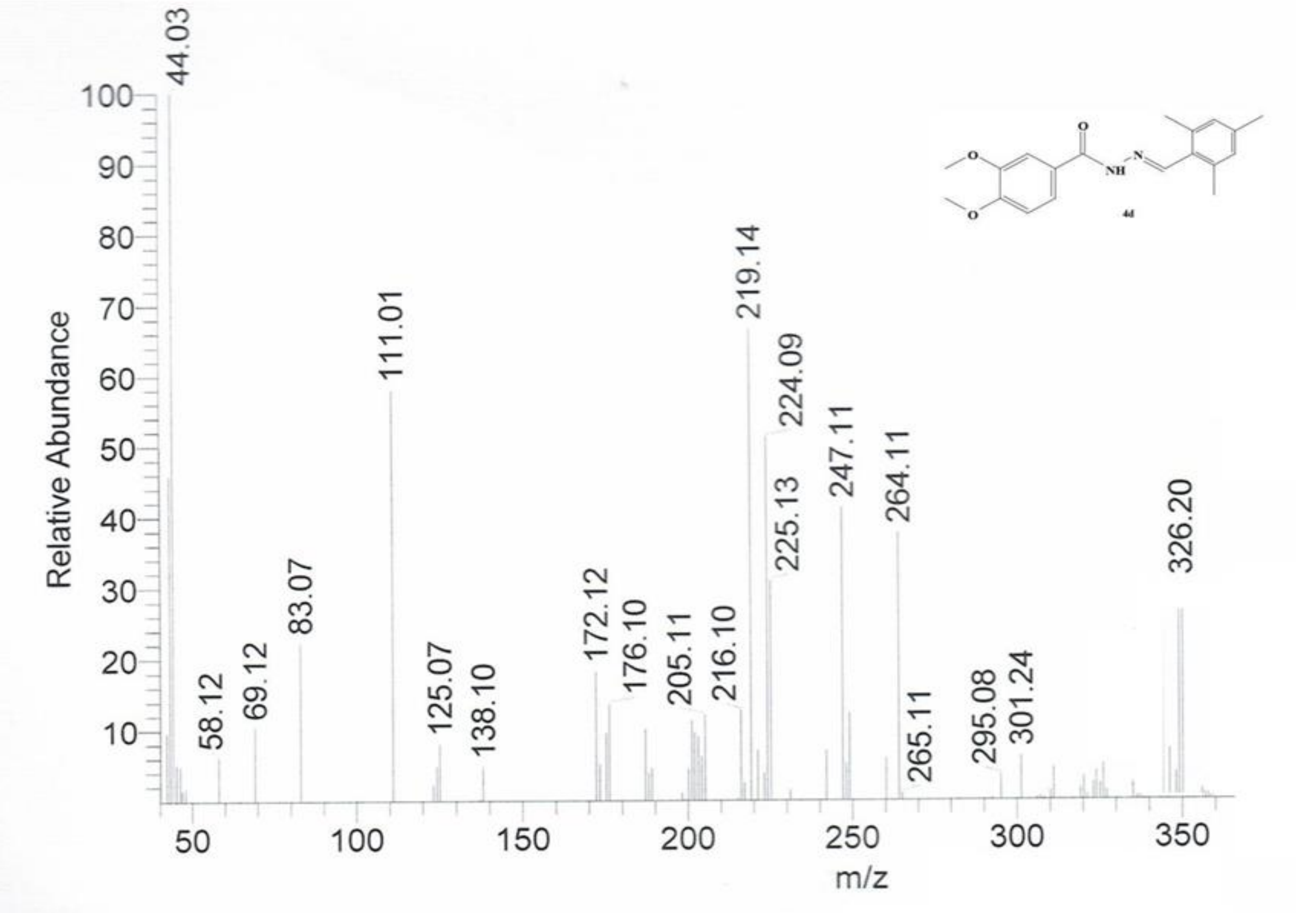
Relative Abundance
m/z
44.03
58.12
69.12
83.07
111.01
125.07
138.10
172.12
176.10
205.11
216.10
219.14
224.09
225.13
247.11
264.11
265.11
295.08
301.24
326.20
4d
100 90 80 70 60 50 40 30 20 10
50 100 150 200 250 300 350

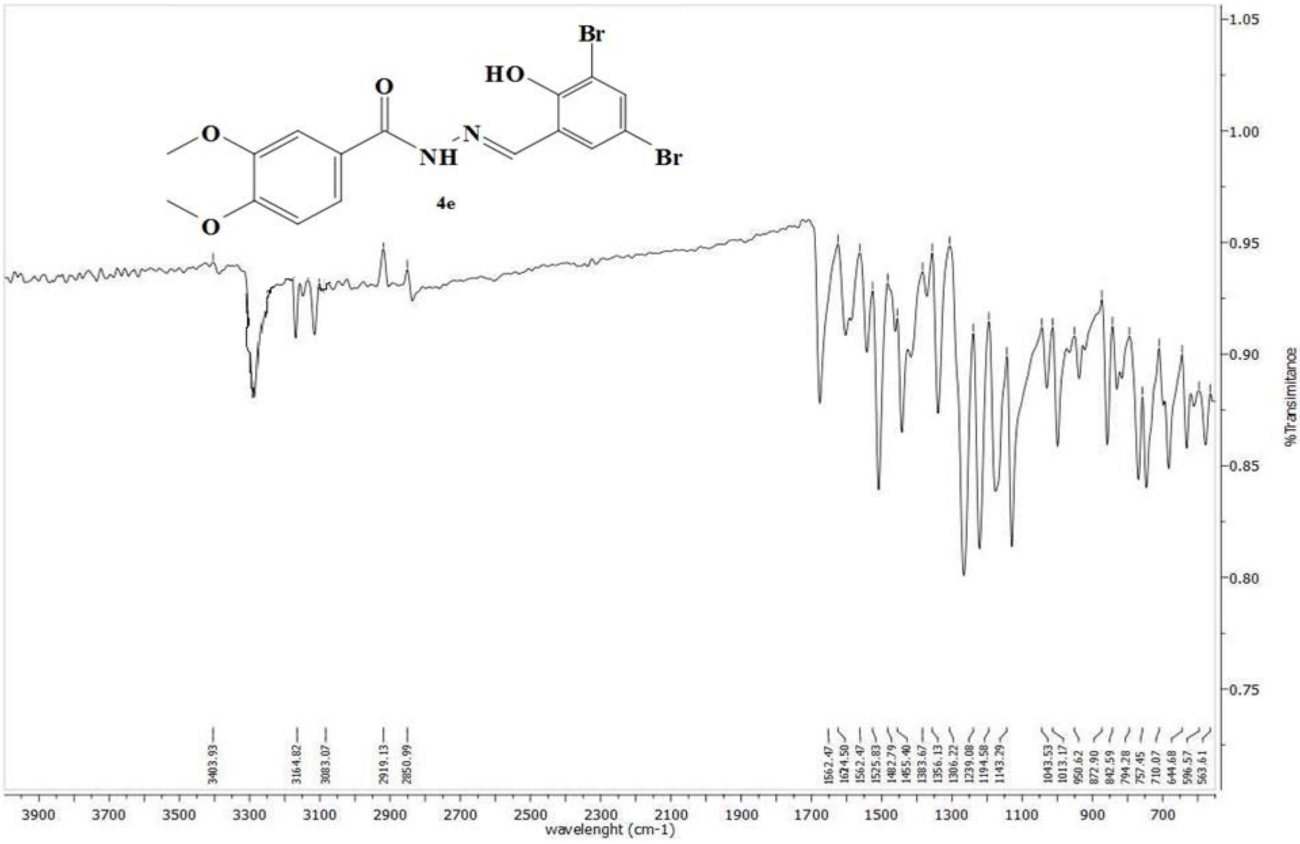

Br
HO
O
N
NH
Br
O
O
4e
%Transimitance
wavelenght (cm-1)

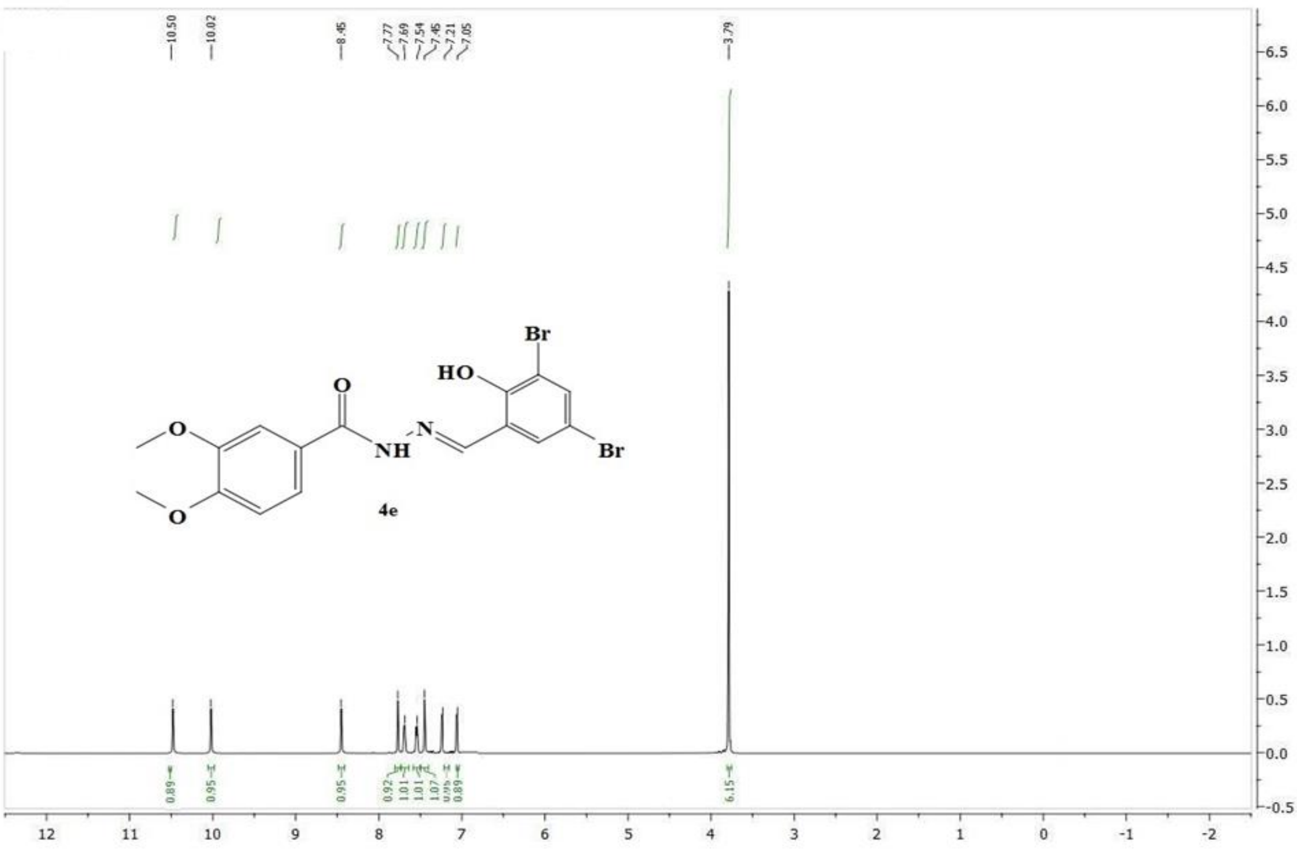

Br
HO
O
N
NH
Br
O
O
4e

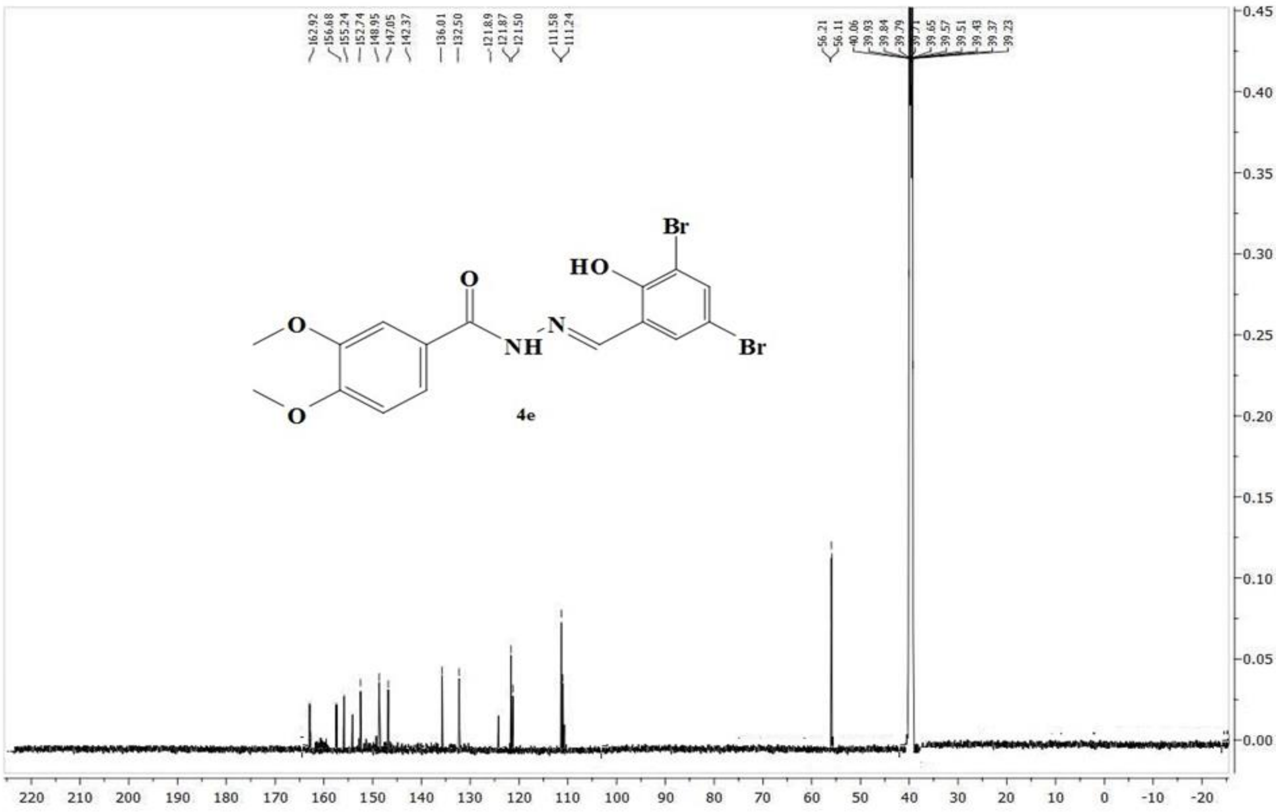

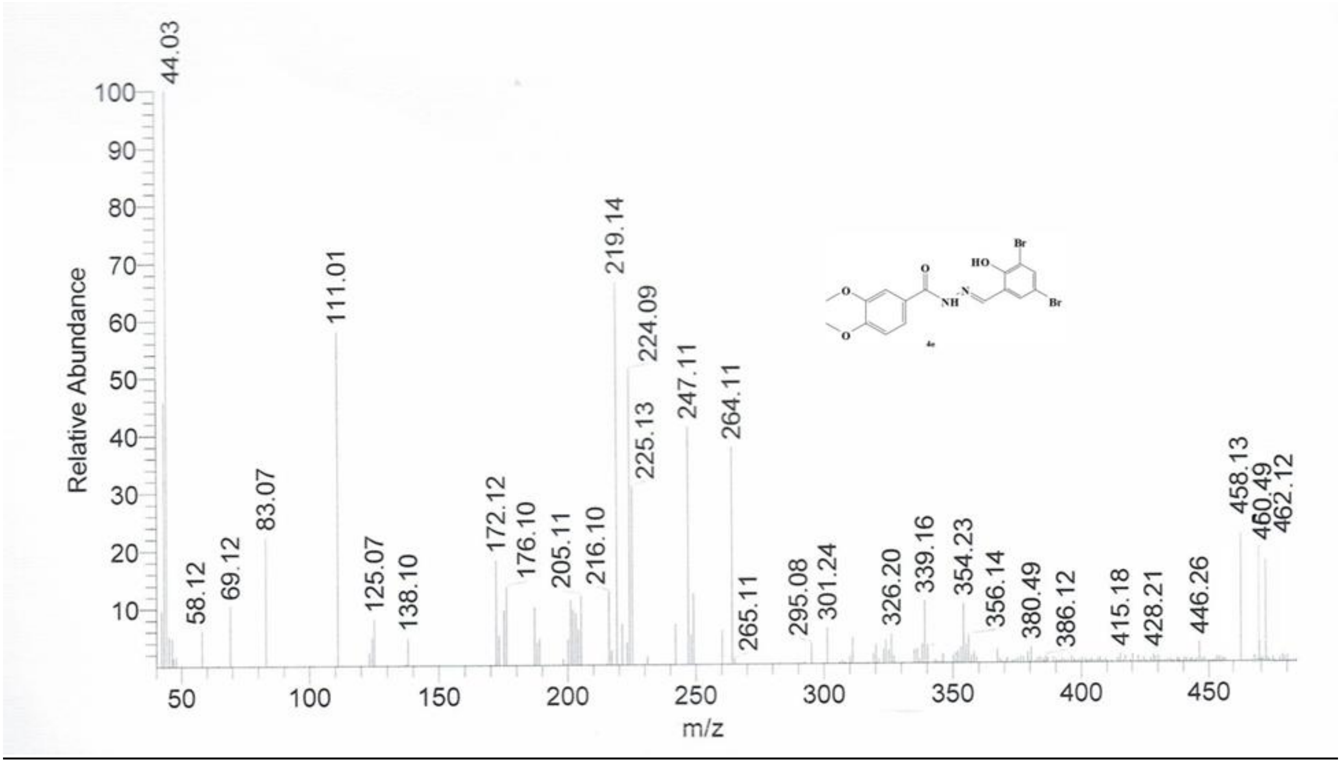
Relative Abundance
m/z
44.03
111.01
219.14
224.09
247.11
264.11
458.13
460.49
462.12

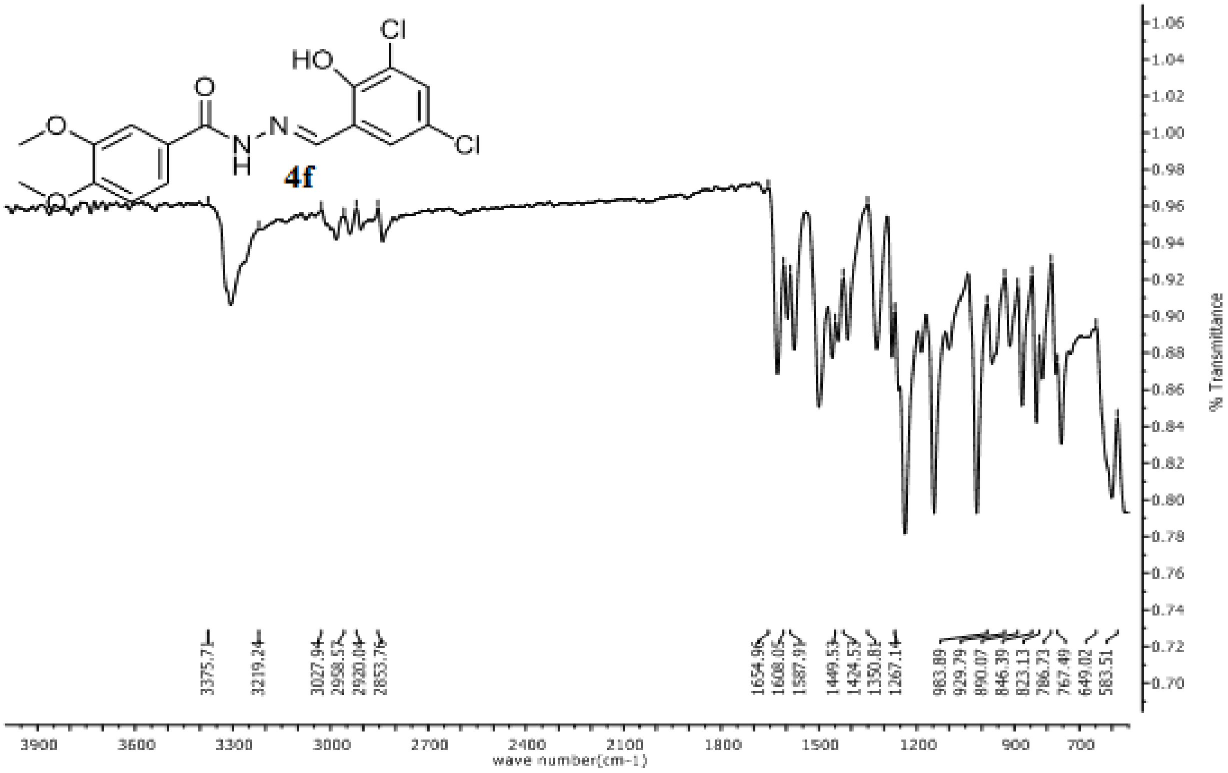
4f
% Transmittance
wave number(cm-1)
3375.71
3219.24
3027.94
2958.57
2920.04
2853.76
1654.96
1608.05
1587.91
1449.53
1424.57
1350.81
1267.14
983.89
929.79
890.07
846.39
823.13
786.73
767.40
649.02
583.51

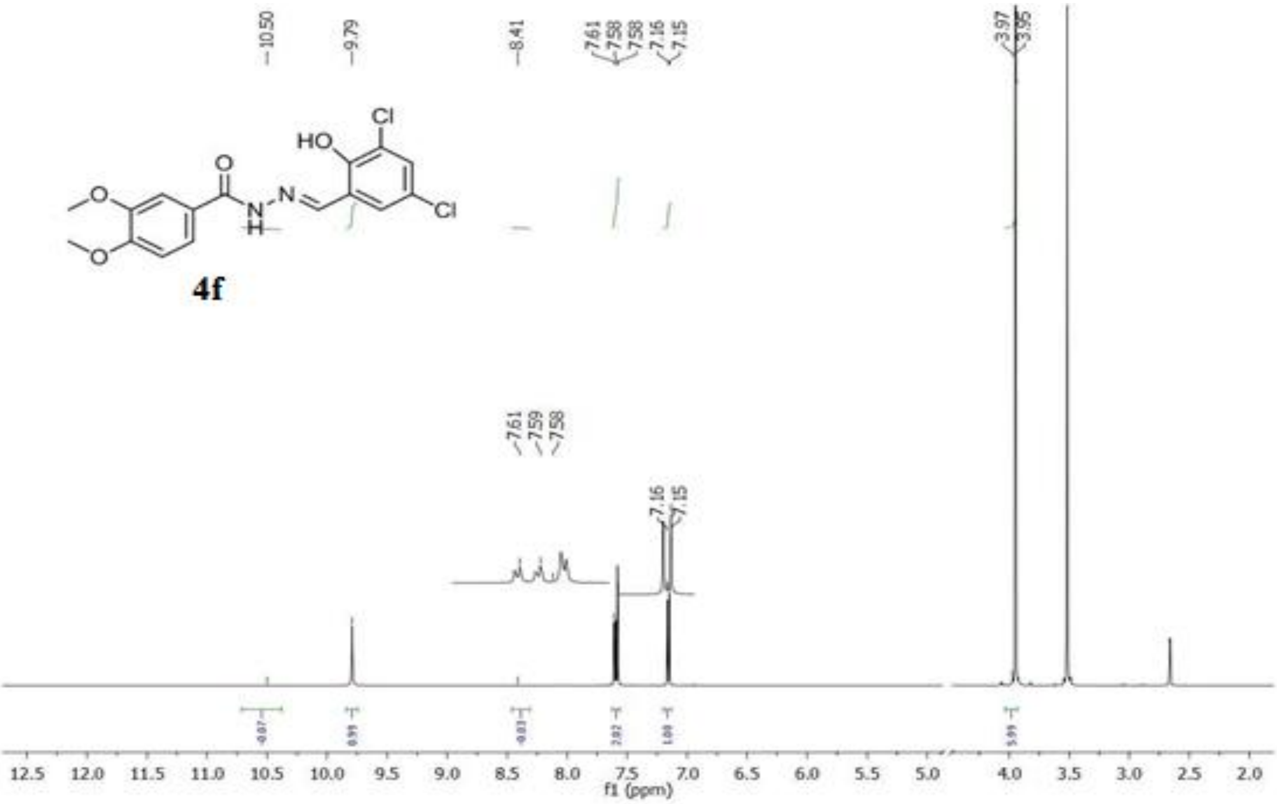
4f
10.50
9.79
8.41
7.61
7.58
7.58
7.16
7.15
3.97
3.95
f1 (ppm)

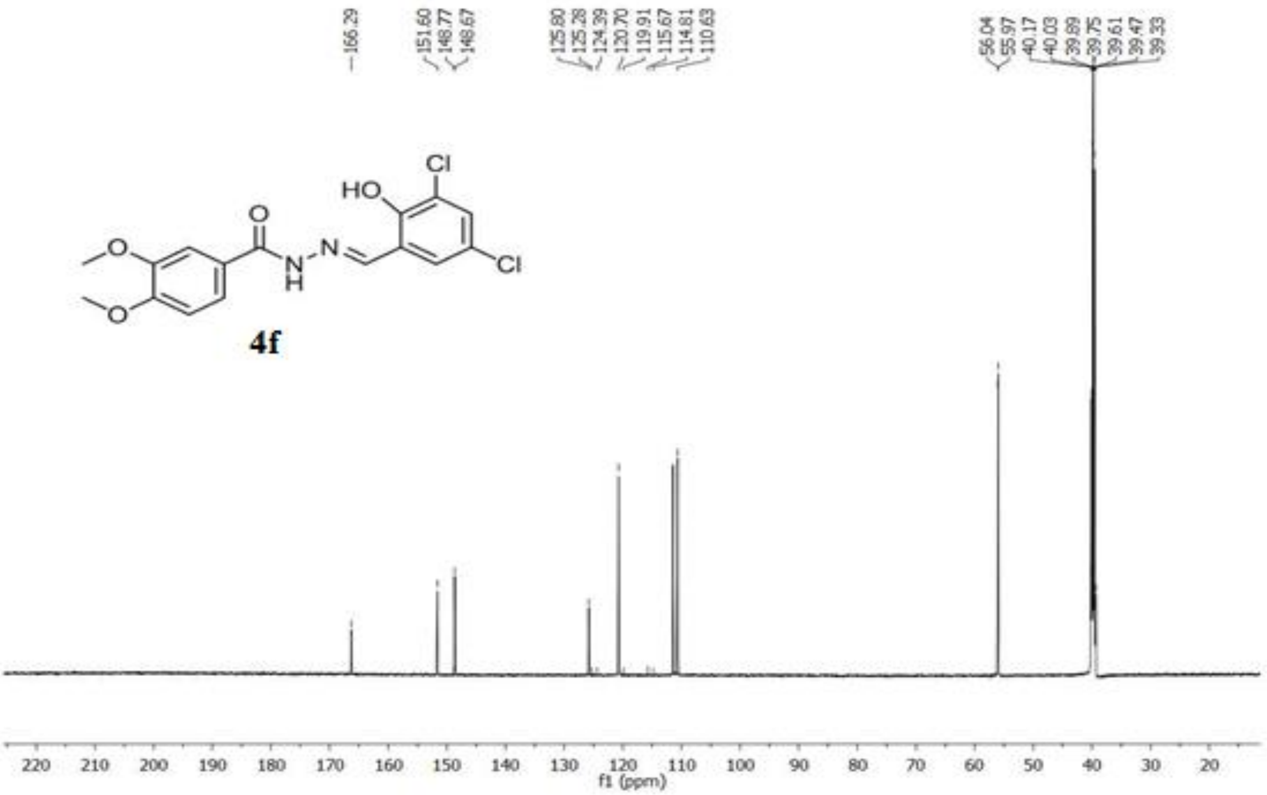
166.29
151.60
148.77
148.67
125.80
125.28
124.39
120.70
119.91
115.67
114.81
110.63
56.04
55.97
40.17
40.03
39.89
39.75
39.61
39.47
39.33
HO
Cl
O
N
H
4f
f1 (ppm)

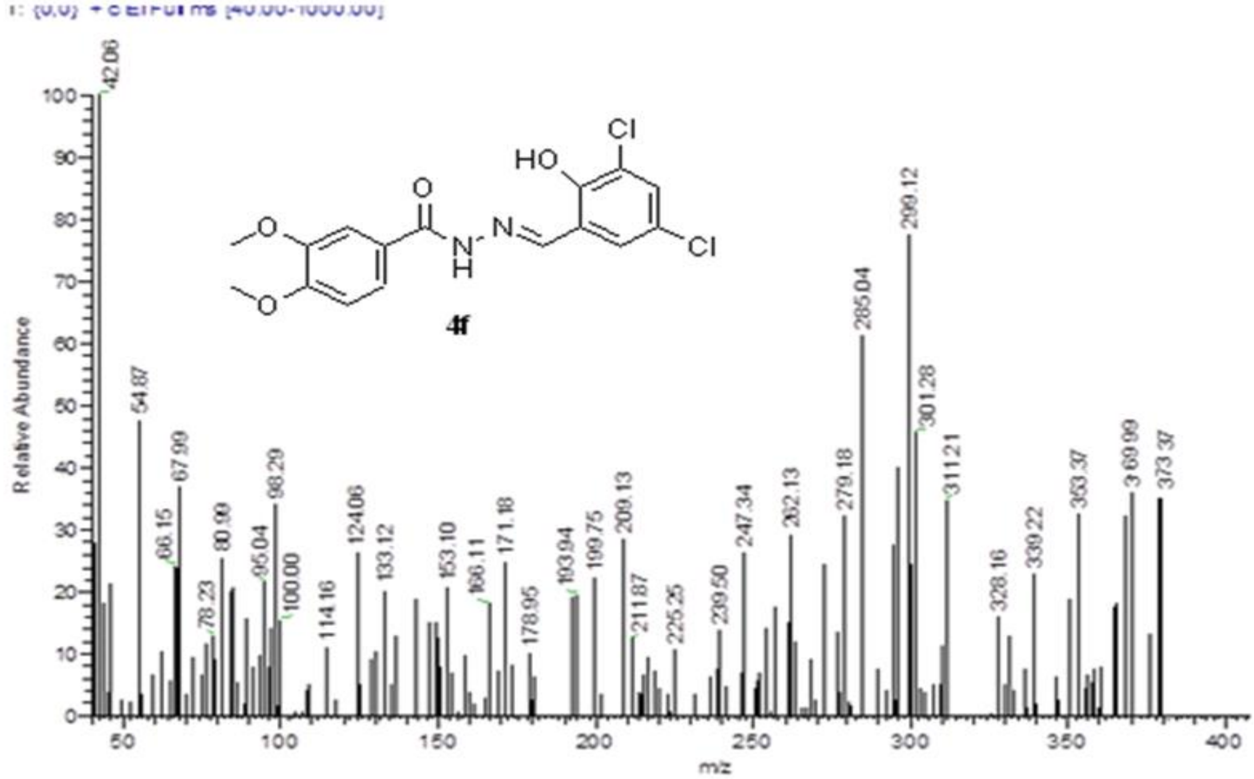
Relative Abundance
m/z
4f
42.06
54.87
67.99
80.99
98.29
124.06
133.12
153.10
171.18
193.94
199.75
209.13
247.34
262.13
279.18
285.04
299.12
301.28
311.21
328.16
339.22
353.37
369.99
373.37

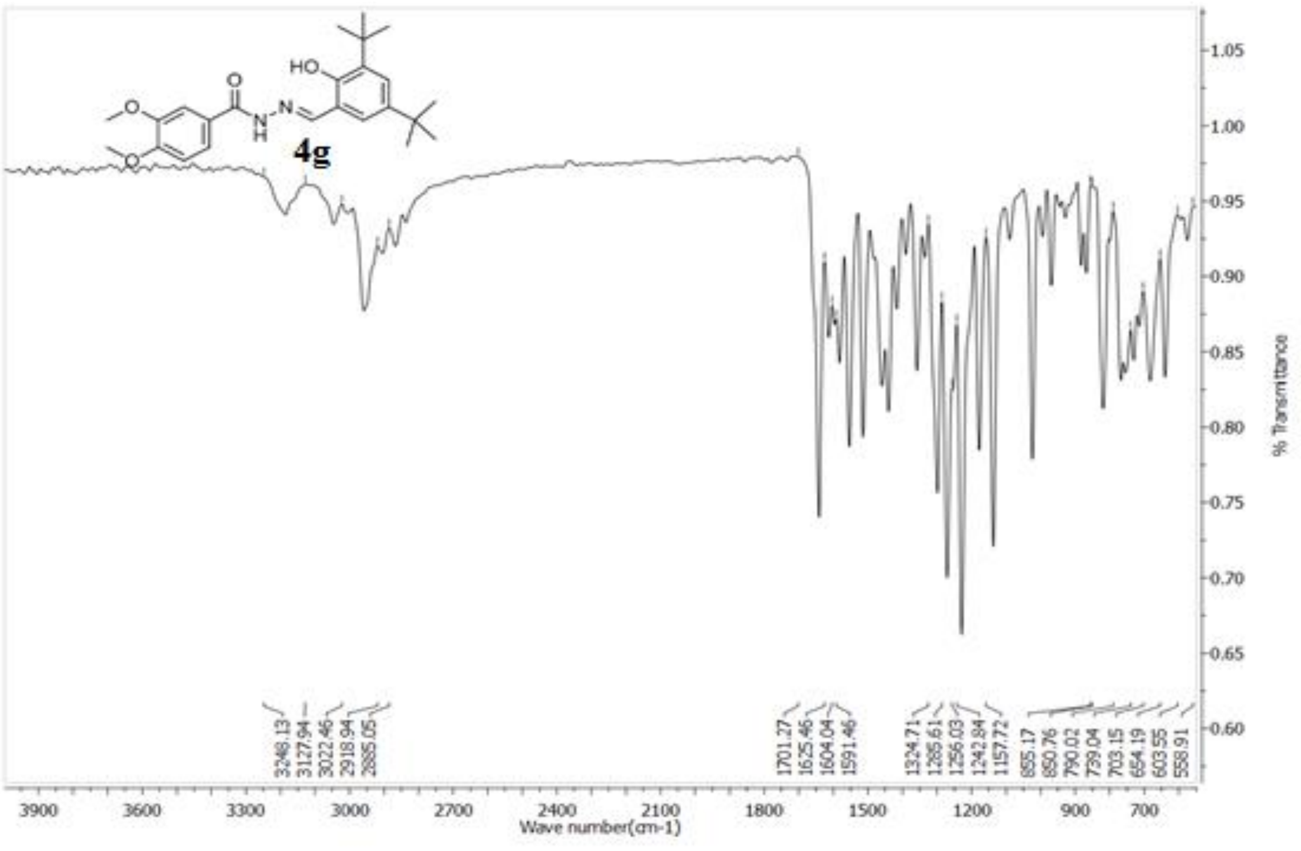
4g
HO
O
N
H
3248.13
3127.94
3022.46
2918.94
2885.05
1701.27
1625.46
1604.04
1591.46
1324.71
1285.61
1256.03
1242.84
1157.72
855.17
850.76
790.02
739.04
703.15
654.19
603.55
558.91
% Transmittance
1.05
1.00
0.95
0.90
0.85
0.80
0.75
0.70
0.65
0.60
3900
3600
3300
3000
2700
2400
2100
1800
1500
1200
900
700
Wave number(cm-1)

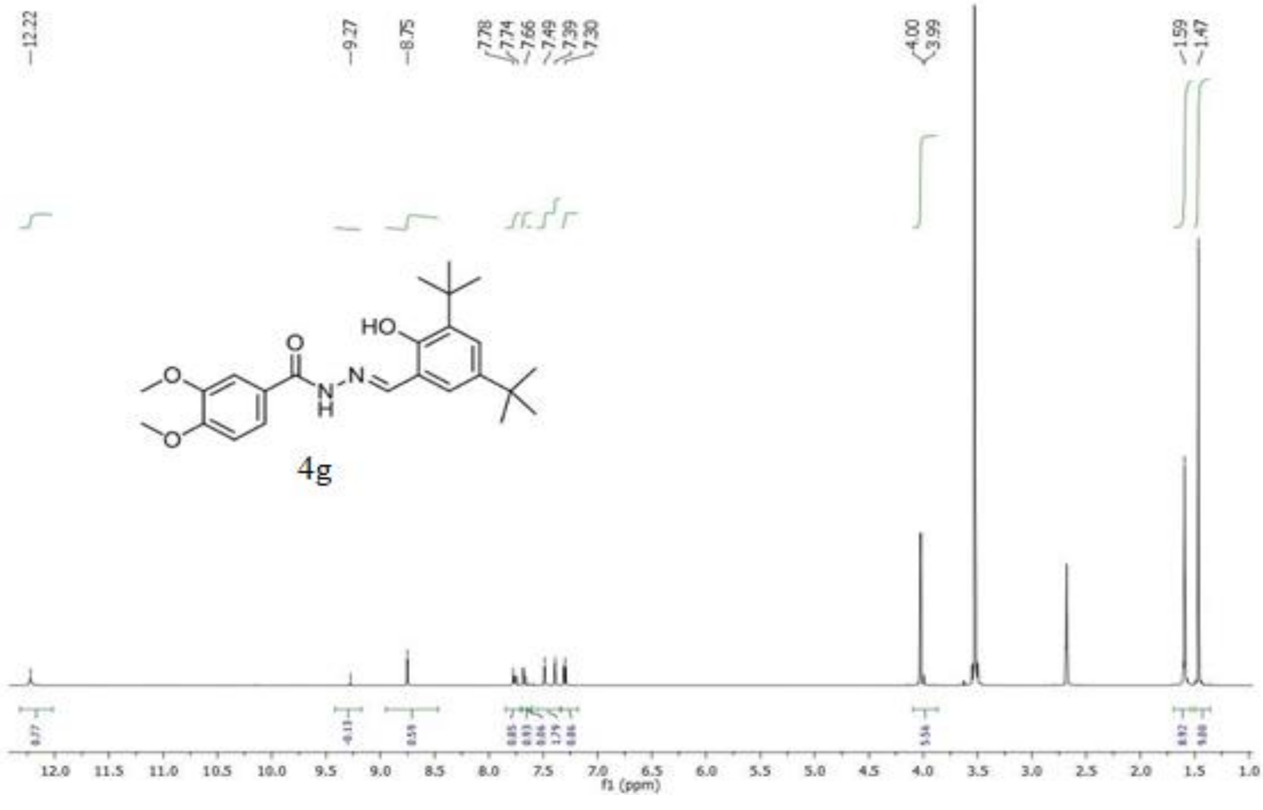
12.22
9.27
8.75
7.78
7.74
7.66
7.49
7.39
7.30
4.00
3.99
1.59
1.47
HO
O
N
H
4g
12.0
11.5
11.0
10.5
10.0
9.5
9.0
8.5
8.0
7.5
7.0
6.5
6.0
5.5
5.0
4.5
4.0
3.5
3.0
2.5
2.0
1.5
1.0
f1 (ppm)

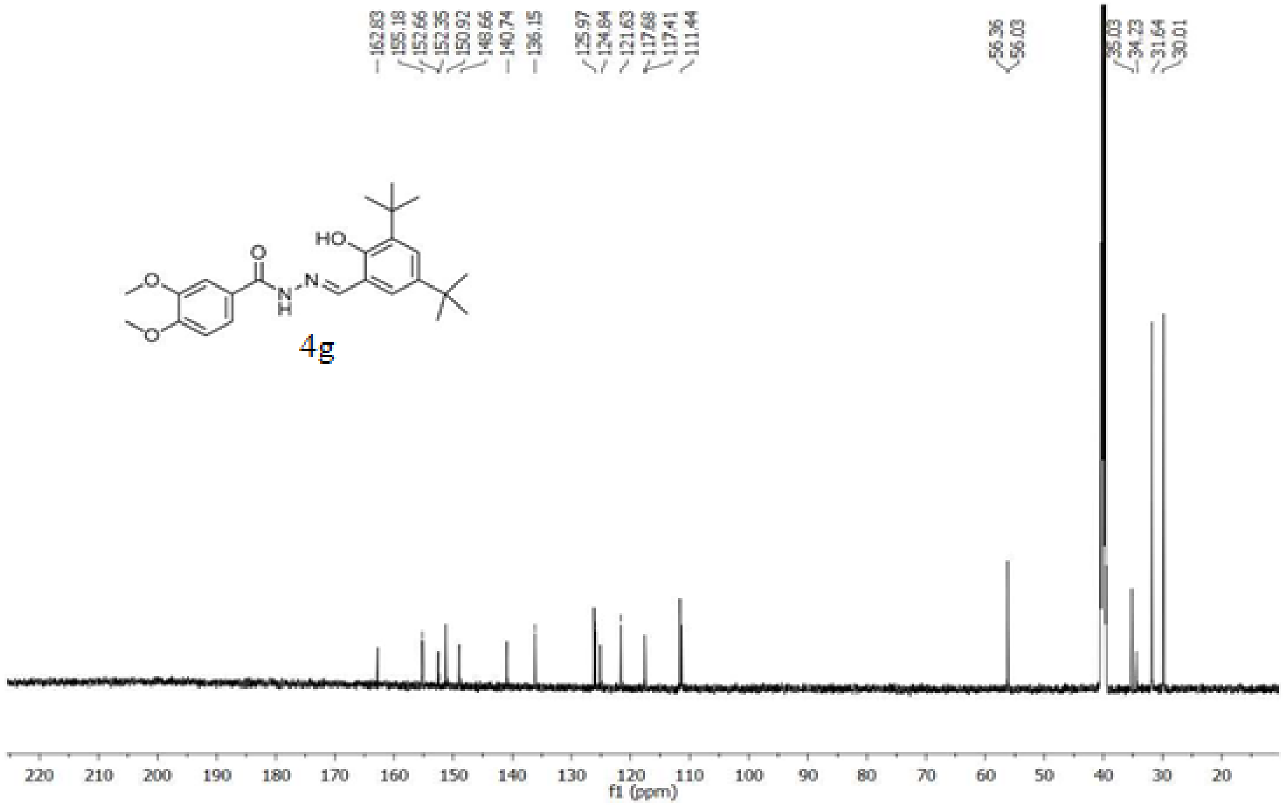
162.83
155.18
152.66
152.35
150.92
148.66
140.74
136.15
125.97
124.84
121.63
117.68
117.41
111.44
56.36
56.03
35.03
34.73
31.64
30.01
HO
O
N
N
H
4g
f1 (ppm)

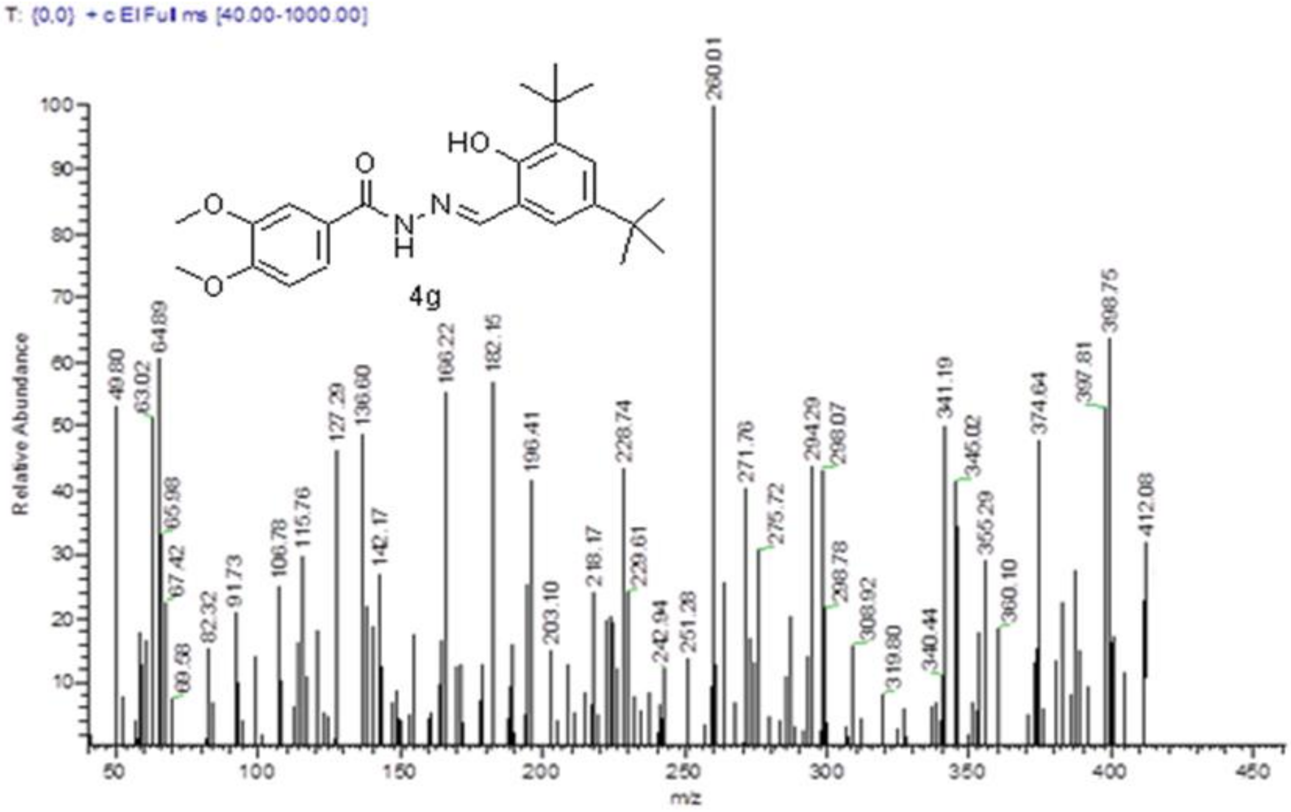
T: {0,0} + c EI Full ms [40.00-1000.00]
Relative Abundance
m/z
HO
O
N
N
H
4g
260.01
398.75
397.81
341.19
374.64
345.02
412.08
355.29
360.10
294.29
298.07
271.76
275.72
228.74
229.61
218.17
182.15
166.22
196.41
203.10
136.60
127.29
142.17
115.76
106.78
91.73
82.32
64.89
63.02
49.80
65.98
67.42
69.58
242.94
251.28
298.78
308.92
319.80
340.44

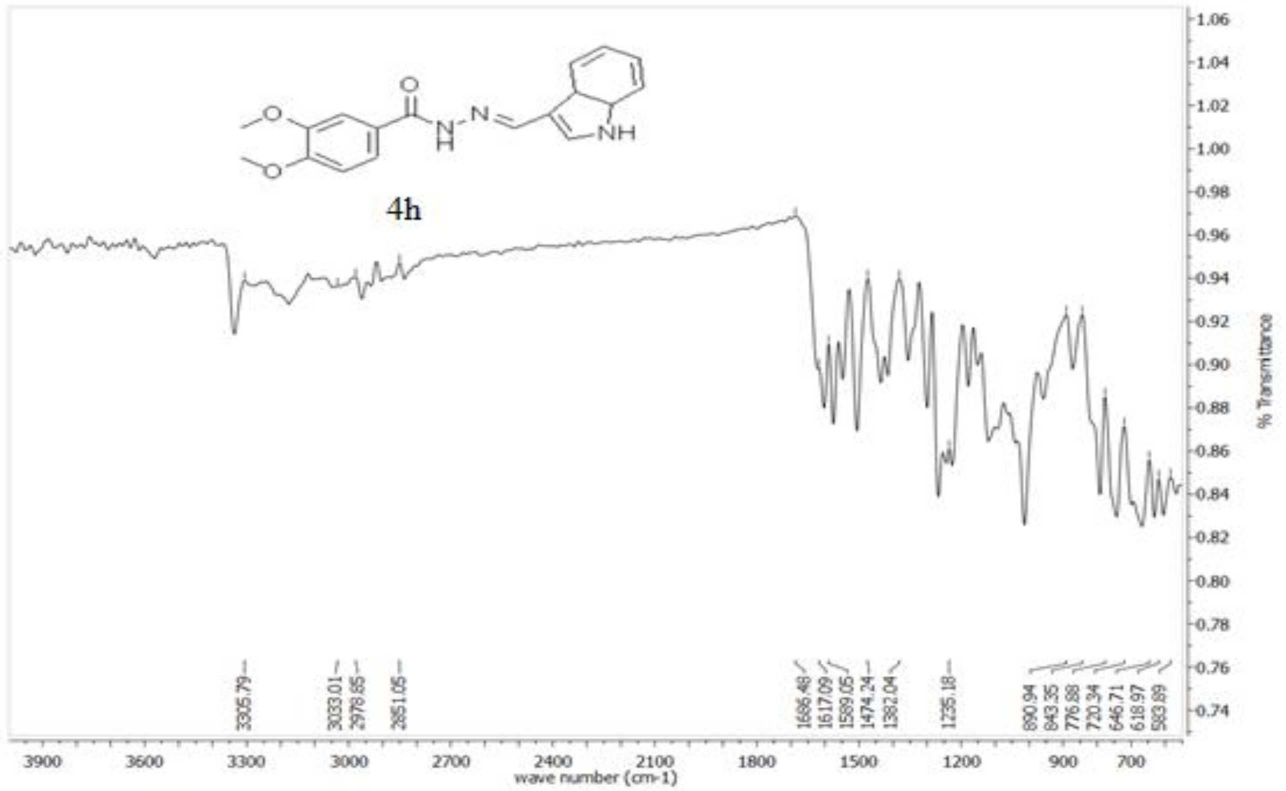
4h
NH
3305.79
3033.01
2978.65
2851.05
1666.48
1617.09
1589.05
1474.24
1382.04
1235.18
890.94
843.35
776.88
720.34
646.71
618.97
583.89
wave number (cm-1)
% Transmittance

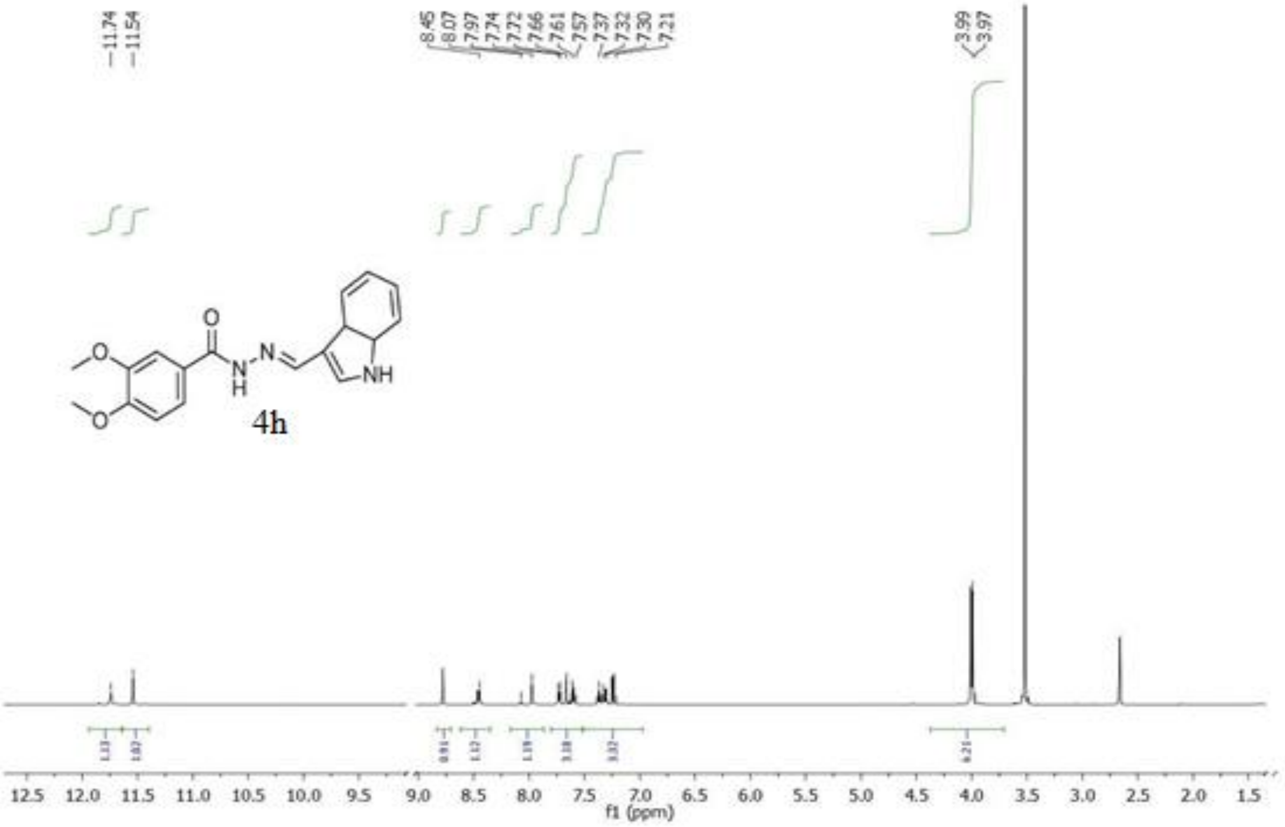
11.74
11.54
8.45
8.07
7.97
7.74
7.72
7.66
7.61
7.57
7.37
7.32
7.30
7.21
3.99
3.97
4h
NH
f1 (ppm)

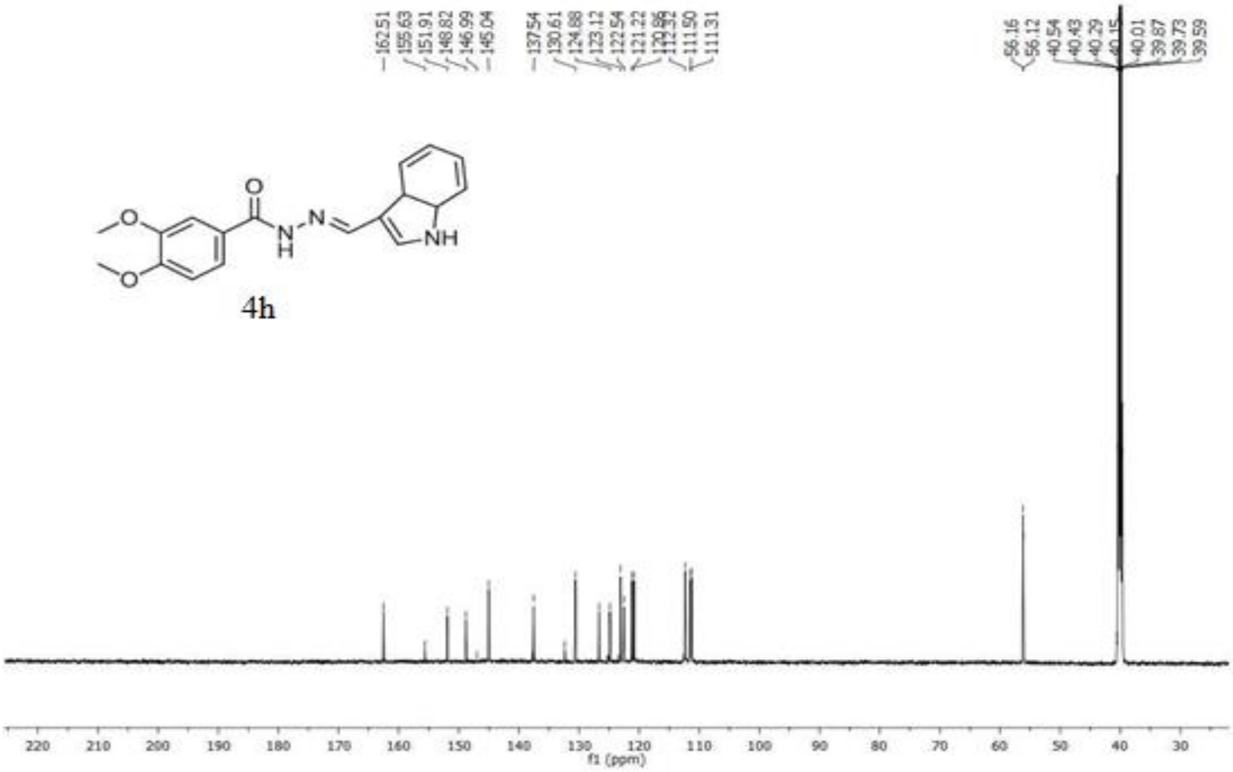

4h

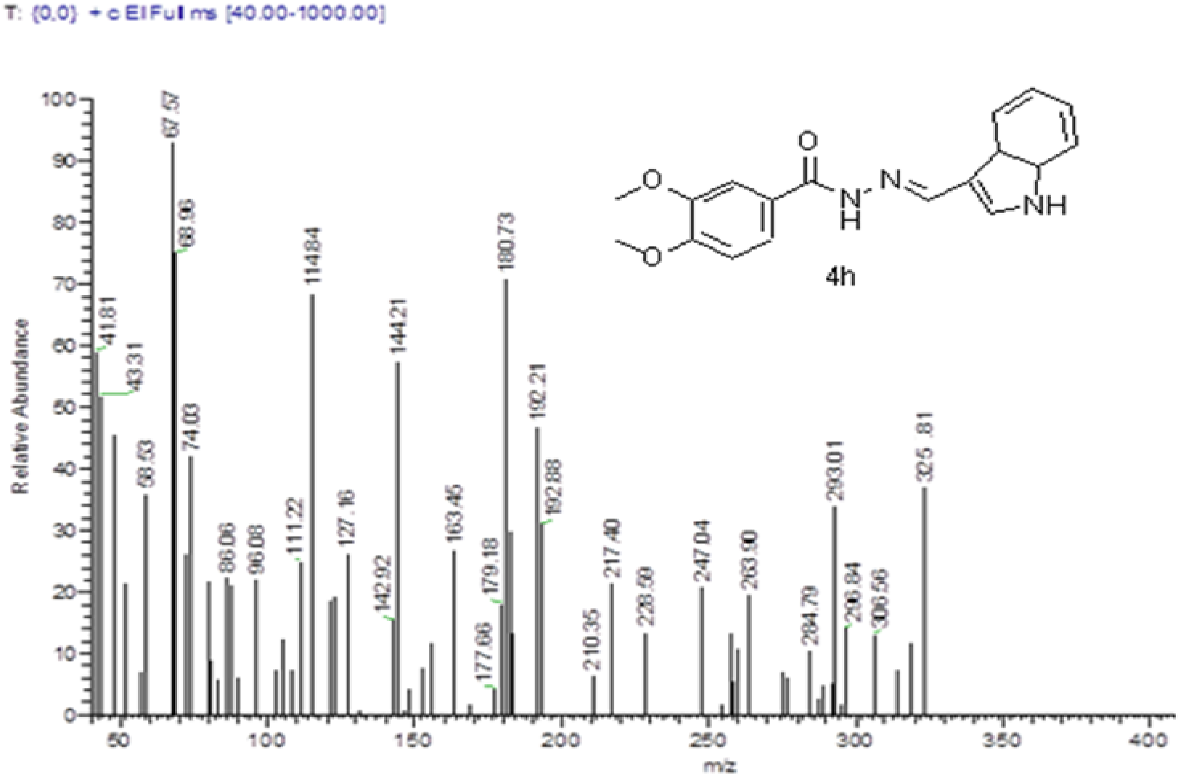

4h

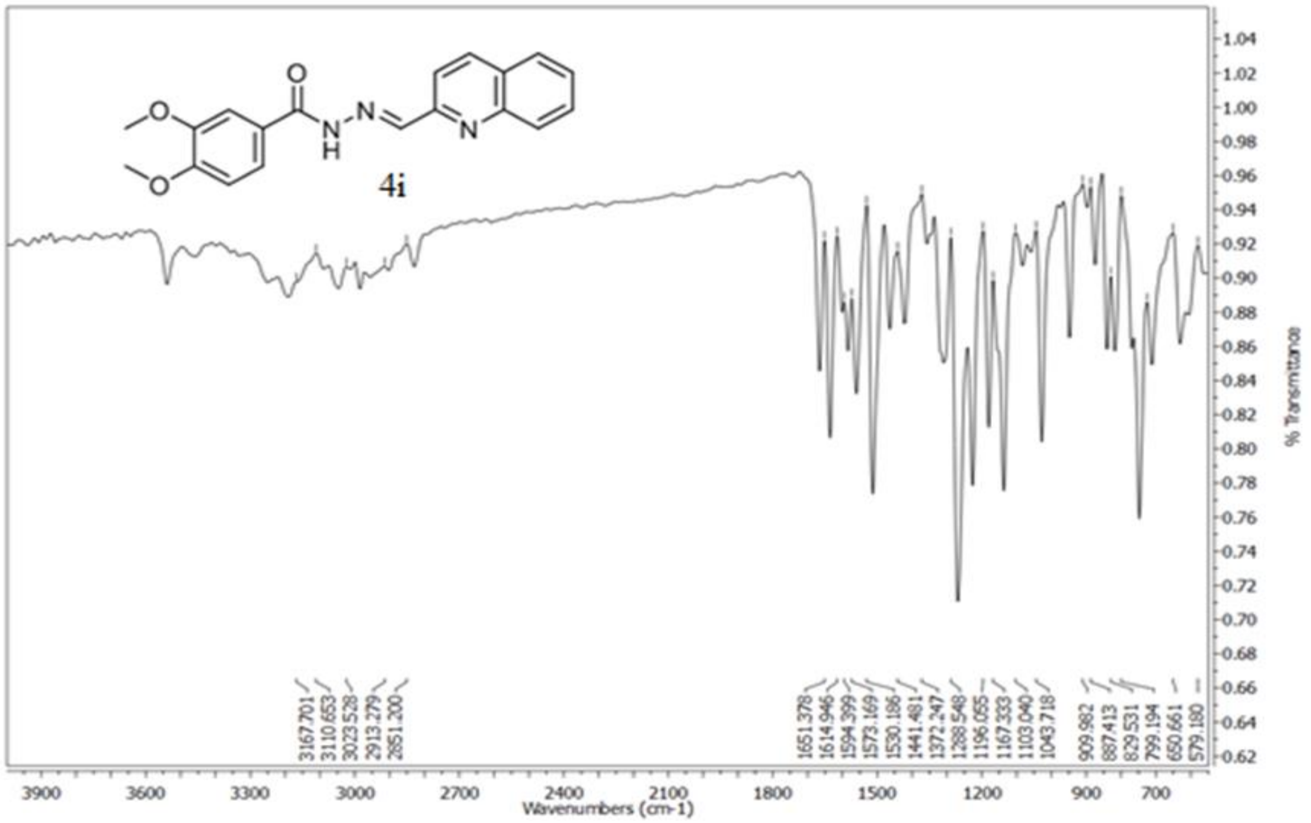
4i
3167.701
3110.653
3023.528
2913.279
2851.200
1651.378
1614.946
1594.399
1573.169
1530.186
1441.481
1372.247
1288.548
1196.055
1167.333
1103.040
1043.718
909.982
887.413
829.531
799.194
650.661
579.180
Wavenumbers (cm-1)
% Transmittance

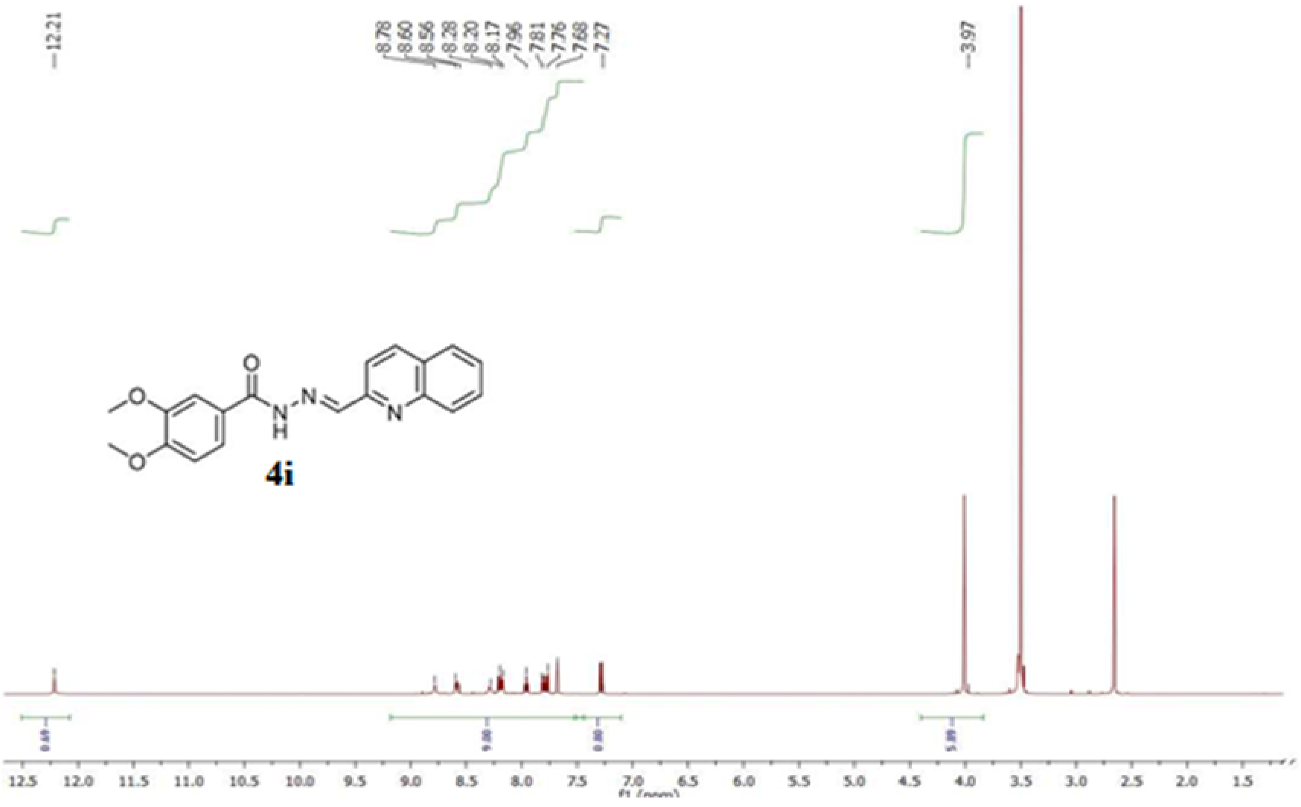
12.21
8.78
8.60
8.56
8.28
8.20
8.17
7.96
7.81
7.76
7.68
7.27
3.97
4i
0.69
9.00
0.80
5.89
f1 (ppm)

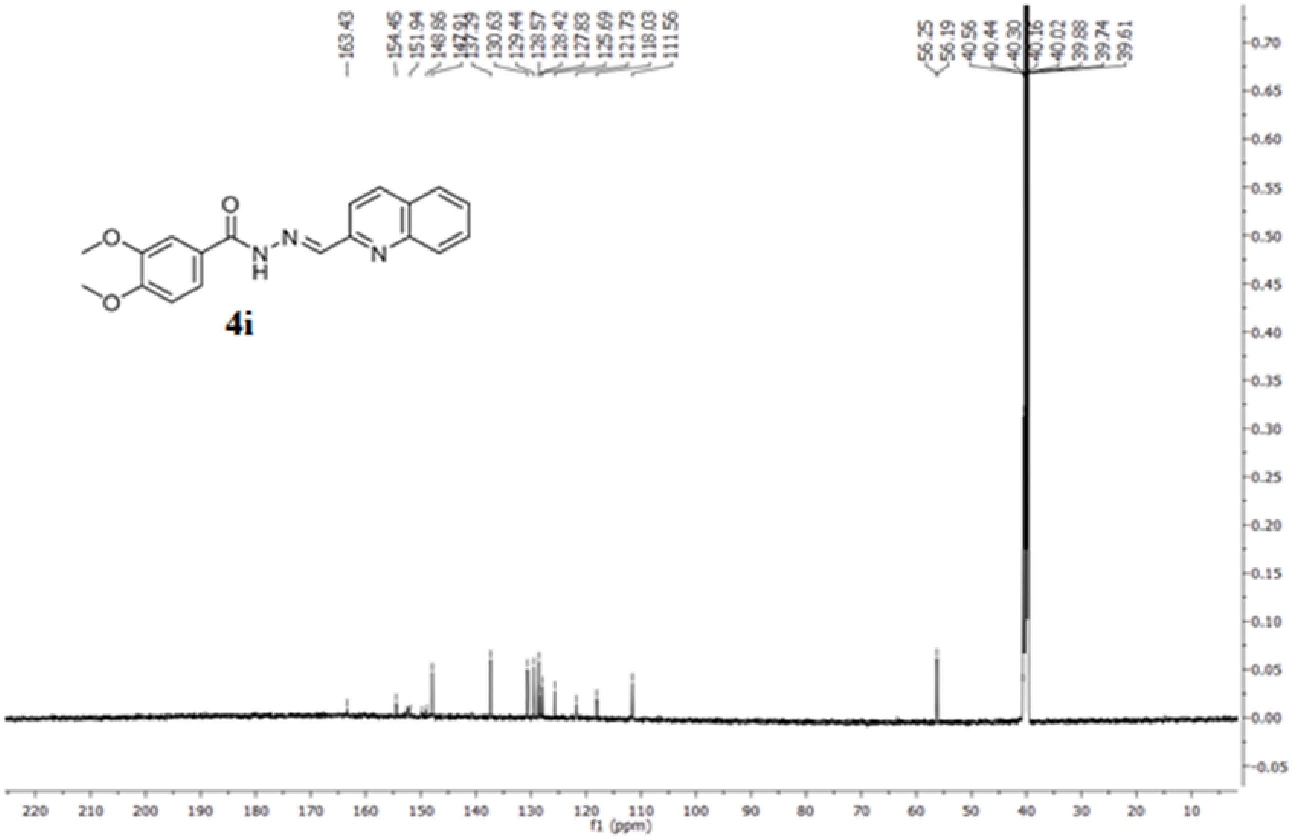
4i
f1 (ppm)

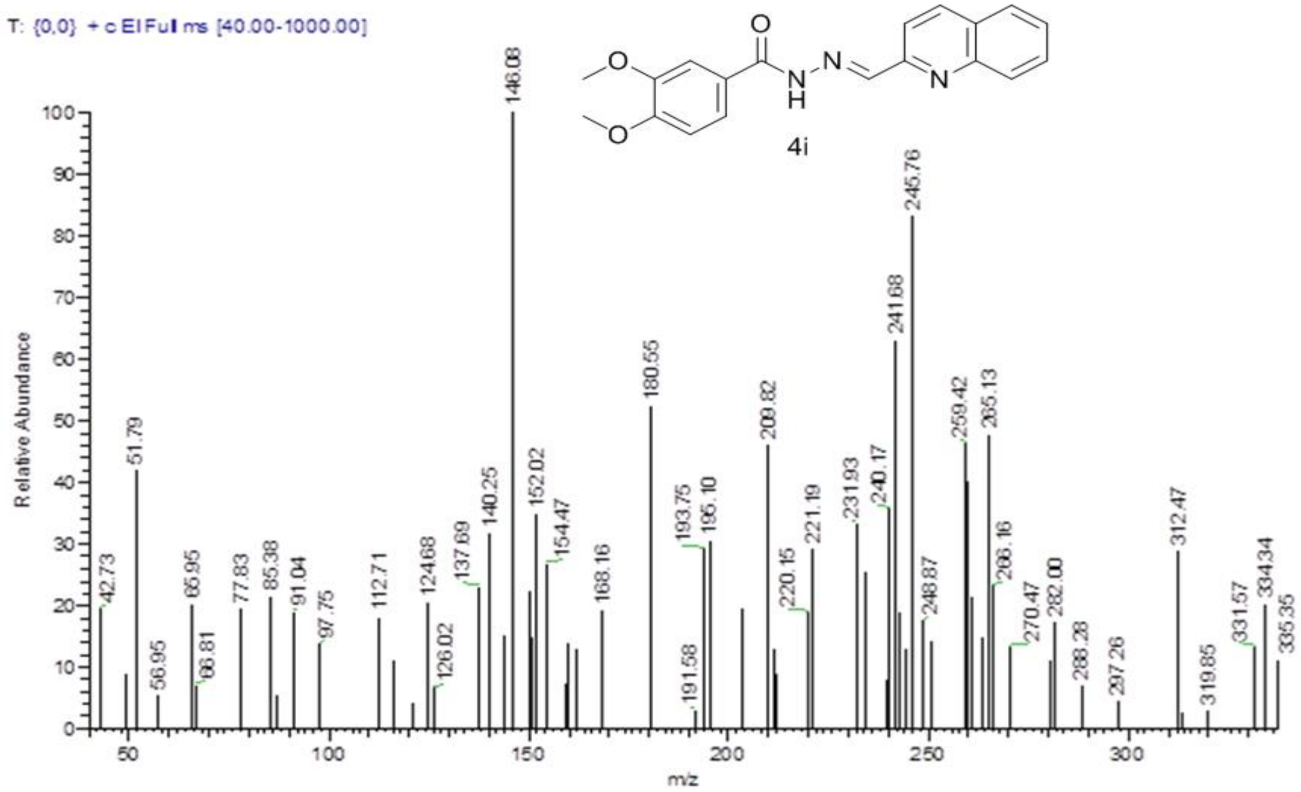
T: {0,0} + c EI Full ms [40.00-1000.00]
4i
Relative Abundance
m/z
146.08
245.76
241.68
180.55
209.82
259.42
265.13
51.79
152.02
140.25
312.47
231.93
240.17
195.10
193.75
221.19
154.47
137.69
124.68
168.16
266.16
334.34
220.15
248.87
65.95
77.83
85.38
91.04
112.71
42.73
270.47
282.00
335.35
331.57
97.75
126.02
66.81
56.95
191.58
288.28
297.26
319.85

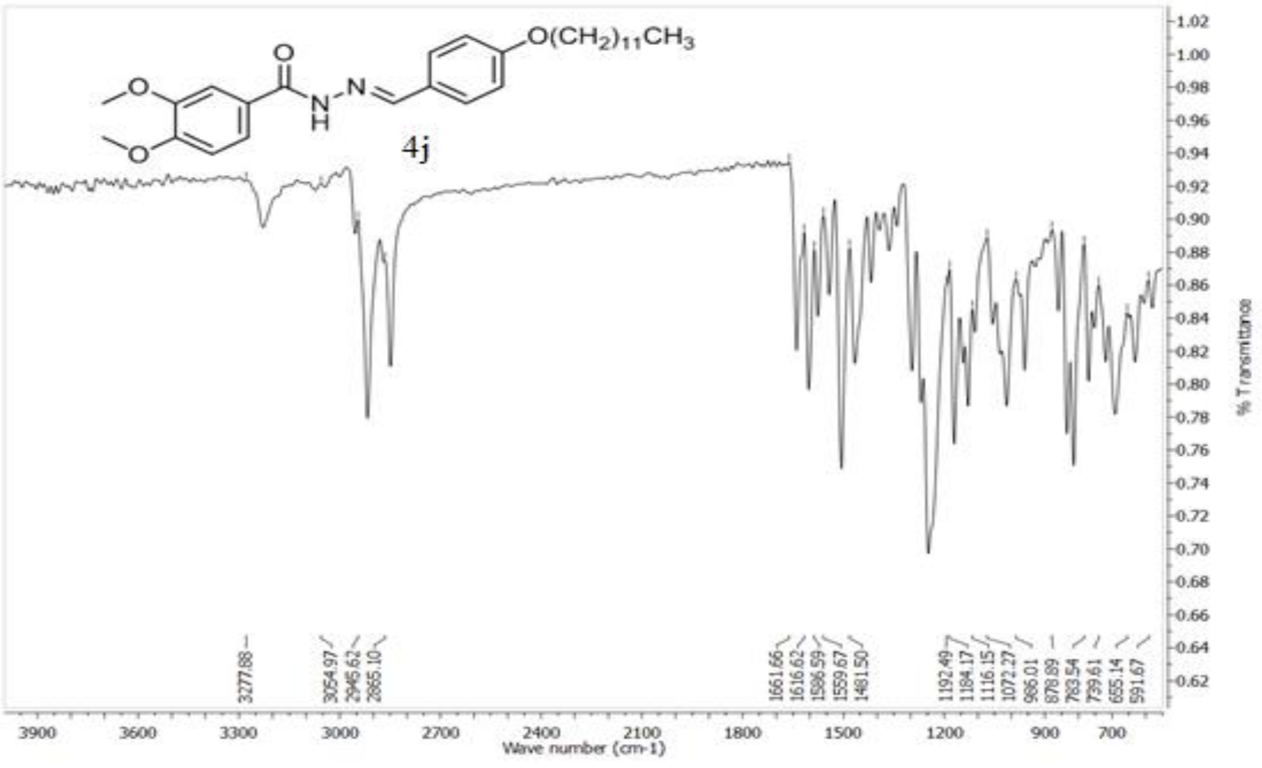
O(CH2)11CH3
4j
Wave number (cm-1)
% Transmittance

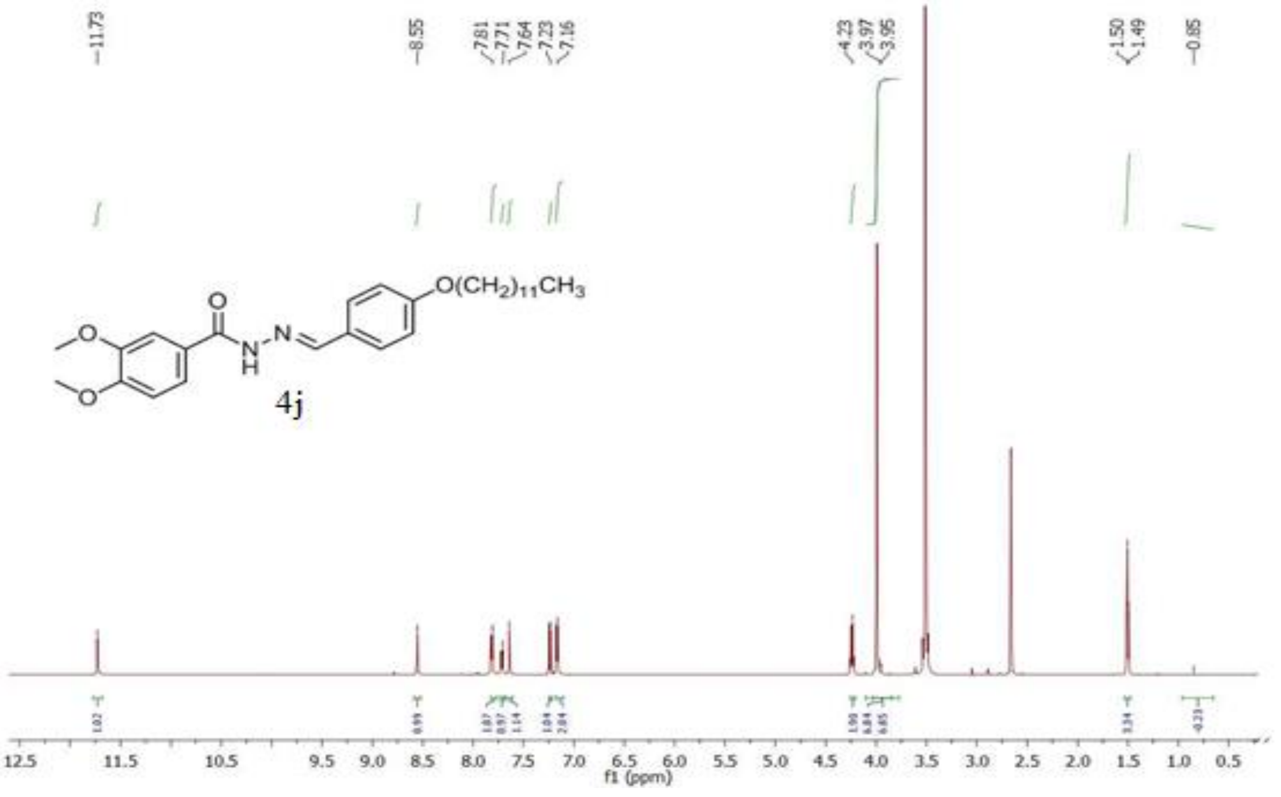
O(CH2)11CH3
4j
f1 (ppm)

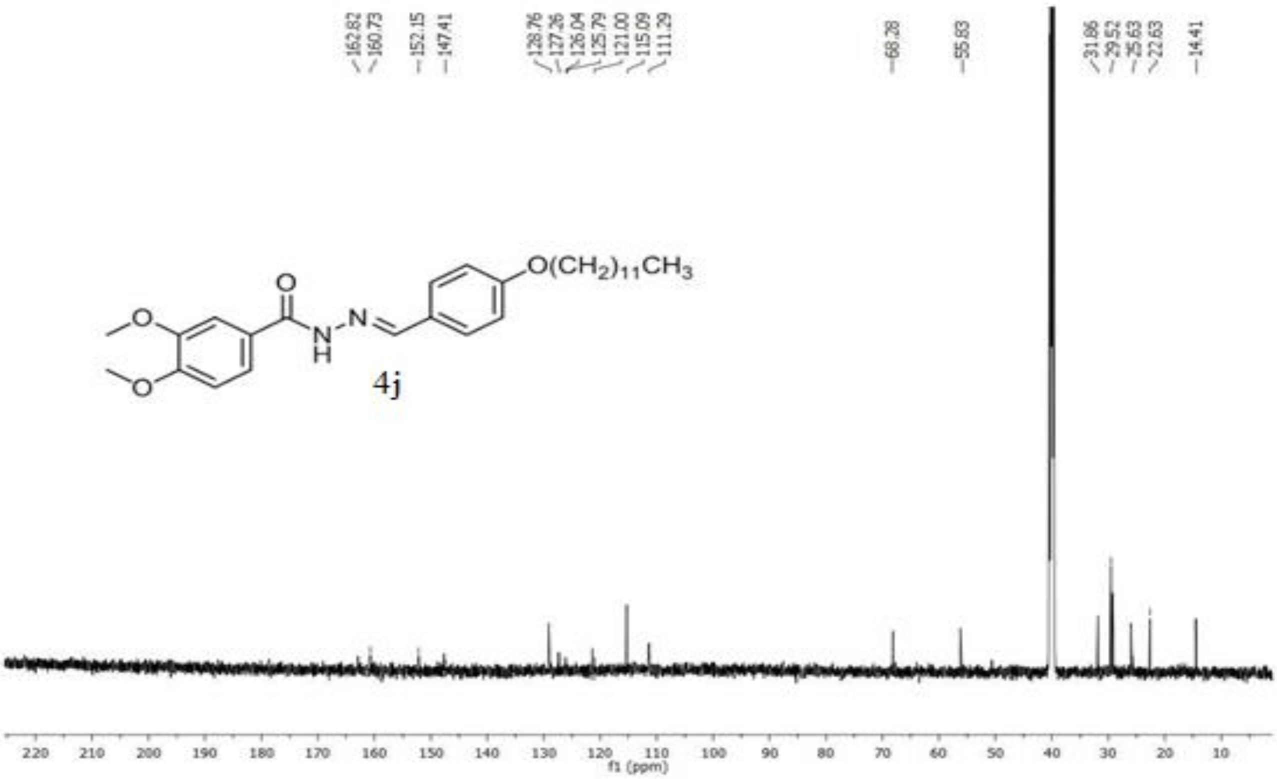

162.82
160.73
152.15
147.41
128.76
127.26
126.04
125.79
121.00
115.09
111.29
68.28
55.83
31.86
29.52
25.63
22.63
14.41
O(CH2)11CH3
4j
f1 (ppm)

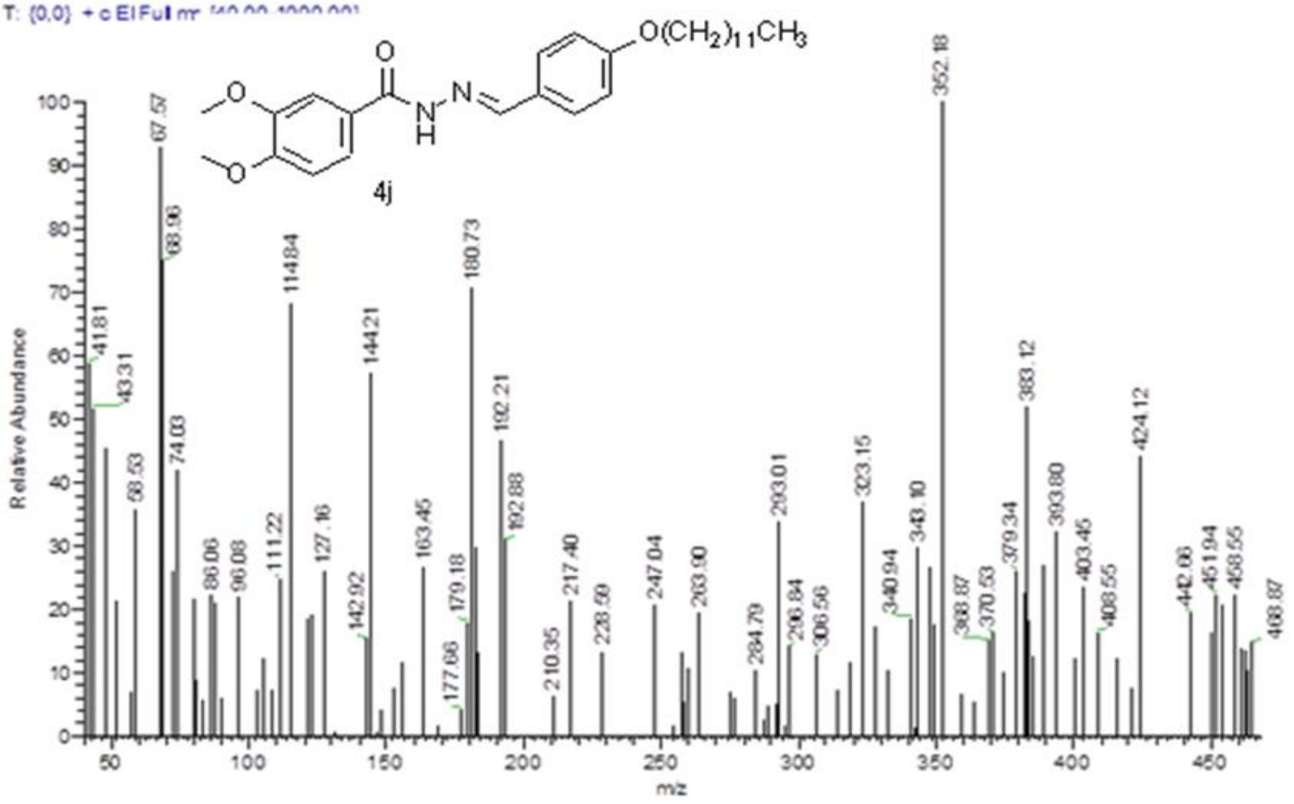

O(CH2)11CH3
4j
Relative Abundance
m/z
352.18
67.57
68.96
180.73
114.84
144.21
41.81
43.31
383.12
424.12
192.21

SI2. Graph (1): Area percentage of **Caspase 3** in liver and kidney of **4h** and **4i** treated groups.

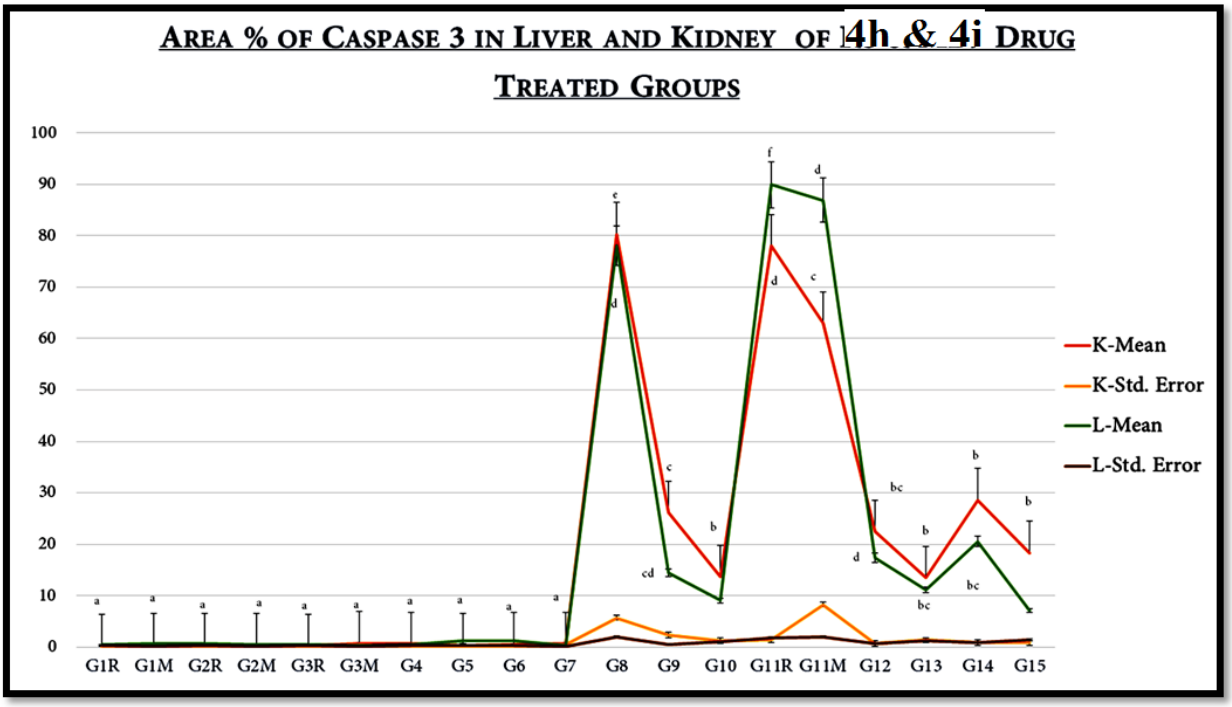

SI3. Graph (2): Area percentage of **NF-kB** in liver and kidney of **4h** and **4i** treated groups.

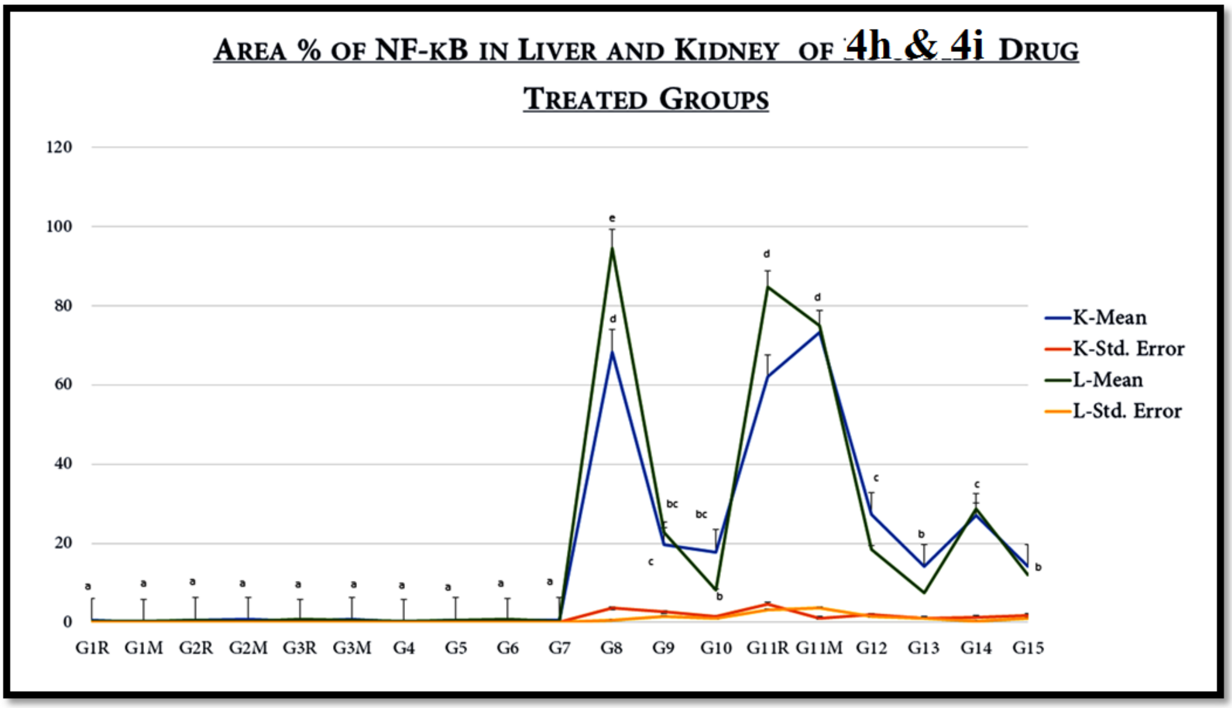

Supplement: Supplemental Material [file IENZ_A_2063282_SM3563.pdf]
